# Integrated analysis of Helicobacter pylori-related prognostic gene modification patterns in the tumour microenvironment of gastric cancer

**DOI:** 10.3389/fsurg.2022.964203

**Published:** 2022-09-30

**Authors:** Kaitian Zheng, Ye Wang, Jiancheng Wang, Congjun Wang, Junqiang Chen

**Affiliations:** ^1^ Department of Gastrointestinal Surgery, The First Affiliated Hospital of Guangxi Medical University, Nanning, China; ^2^ Guangxi Key Laboratory of Enhanced Recovery After Surgery for Gastrointestinal Cancer, The First Affiliated Hospital of Guangxi Medical University, Nanning, China; ^3^ Guangxi Clinical Research Center for Enhanced Recovery After Surgery, The First Affiliated Hospital of Guangxi Medical University, Nanning, China; ^4^ Guangxi Zhuang Autonomous Region Engineering Research Center for Artificial Intelligence Analysis of Multimodal Tumor Images, The First Affiliated Hospital of Guangxi Medical University, Nanning, China

**Keywords:** *Helicobacter pylori*, gastric cancer, tumour microenvironment, cell stemness, prognostic model

## Abstract

**Background:**

*Helicobacter pylori* (HP) infection is one of the leading causes of gastric cancer (GC). However, the interaction between HP and the TME, and its carcinogenic mechanism remains unknown.

**Methods:**

The HP-related prognostic genes were identified based on HP infection-related gene markers and HP infection sample datasets by risk method and NMF algorithm. Principal component analysis (PCA) algorithm was used to constructed the HPscore system. The “limma” R package was employed to determine differentially expressed genes. In addition, the R packages, such as “xCell” and “GSVA”, was used to analyze the relationship between the HPscore and tumor microenvironment. Finally, quantitative real-time polymerase chain reaction (qRT-PCR) was conducted to verify the expression levels of 28 HP-related prognostic genes in tissues.

**Results:**

We successfully identified 28 HP-related prognostic genes that accurately classified the GC population. There are significant differences in survival between different subgroups (high-, low-risk and cluster_1,2). Thereafter, the HPscore system was constructed to evaluate the signatures of the 28 HP-related prognostic genes. The overall survival rate in the high-HPscore group was poor and immunological surveillance was reduced, whereas the low-HPscore group had a survival advantage and was related to the inflammatory response. HPscore was also strongly correlated with the tumour stage, TME cell infiltration and stemness. The qRT-PCR results showed that DOCK4 expression level of 28 HP-related prognostic genes was higher in gastric cancer tissues than in adjacent tissues.

**Conclusions:**

HP signatures play a crucial role in the TME and tumourigenesis. HPscore evaluation of a single tumour sample can help identify the TME characteristics and the carcinogenic mechanism of GC patients infected with HP, based on which personalized treatment can be administered.

## Introduction

Gastric cancer (GC) is the fifth-largest type of malignant tumor globally, and its high mortality makes it the third leading cause of cancer-related death (1). It is closely associated with Helicobacter pylori (HP) infection. The World Health Organisation has listed HP as the first group of carcinogens causing gastric adenocarcinoma (2). Because HP is not an intracellular pathogen, continuous inflammation does not effectively eliminate HP but leads to epithelial cell damage. Further, the constant production of reactive oxygen species continues to cause DNA damage, which initiates the cascading reactions that lead to cancer development (3). A study showed that HP eradication therapy reduces the risk of GC in patients with first-degree relatives who have a family history of GC (4). Unfortunately, most patients are prone to drug resistance against HP and the infection cannot be eradicated. A recent observational study confirmed that HP infection can be completely eradicated in only 35% of patients who receive the follow-up treatment for this infection (2). In addition, the understanding of the carcinogenic mechanism of HP is still not comprehensive. Increasing evidence has suggested that the accumulation of bone marrow-derived dendritic cells (BMDCs) induced by HP is one of the origins of GC stem cells. Chronic HP infection leads to chronic inflammation and subsequent gastric epithelial mucosal damage, leading to the recruitment of BMDCs (5). BMDCs exhibit the phenotype and characteristics of cancer stem cells (CSCs) and obtain the ability to differentiate into gastric epithelial cells possibly through cell fusion (6, 7). This mechanism involves the secretion of various cytokines by infected epithelial cells, of which tumour necrosis factor-α (TNF- α) plays a significant role mainly through the NF-kB-dependent pathway (8). HP has been known to activate the typical NF-kB signal in gastric epithelial cells, and its mechanism depends on the type IV secretory system (T4SS) encoded by the CagA pathogenicity island of HP (9). Simultaneously, the inflammatory response caused by HP makes the tumour microenvironment (TME) more complex. With the transition from acute inflammation to chronic inflammation, the virulence factors released by HP prevent the differentiation of immune killer cells and promote the accumulation of immunosuppressive cells (9). In addition, HP activates tumour-associated fibroblasts by activating the IL-17 pathway to assist tumour cells in immune escape (10, 11). Further, the accumulation of a large number of fibroblasts makes it difficult for immune cells to enter the tumour core and provides the necessary conditions for angiogenesis. Therefore, identifying the characteristics of HP-mediated gastric epithelial cell infiltration can help in strengthening our understanding of the complex and changeable TME.

In this study, we identified the prognostic gene markers associated with HP infection in patients with GC. These genes showed a strong correlation with tumour immune-infiltrating cells, and to some extent, participated in the signal pathway of tumour stem cells and then affected tumour progression. We constructed an HPscore system by using HP-related prognostic genes to comprehensively evaluate the TME modification patterns in patients with GC. Elucidation of the overall mechanism of HP infection can help us understand its carcinogenic nature and develop effective treatment strategies.

## Materials and methods

### Collection and preprocessing of datasets

**Figure 1:**
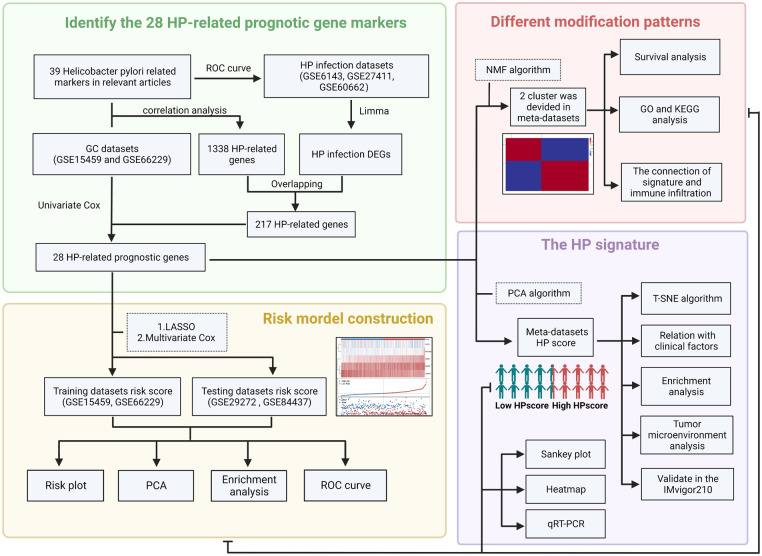
The flow chart of the present study (created with BioRender.com).

The flowchart (Figure 1) and mechanism diagram were plotted in the BioRender (https://app.biorender.com). First, we retrieved HP-related studies published in the past 3 years from the NCBI and Web of Science to verify the HP-infected related gene markers (the following unified abbreviated as HP). To investigate the relationship between HP infection and GC, we collected relevant datasets from the GEO and TCGA databases. In summary, 4 HP infection-related datasets (i.e. GSE6143, GSE5081, GSE27411, and GSE60662), 5 GC datasets (i.e., GSE66229, GSE29272, GSE84437, GSE15459, and TCGA-STAD) with OS data, three drug treatment datasets (i.e., PD-L1/IMvigor210). The ROC curve and the AUC value were used to evaluate the diagnostic efficacy of HP-related genes. The “survival” and “survminer” R packages were used to draw the survival curve of the GC datasets. To eliminate the batch effects of different datasets, we used the “combat” algorithm of the “SVA” R package to merge the datasets (i.e., GSE66229 and GSE15459). The “FactoMineR” and “Factoextra” R packages were used to demonstrate the fit effect of the meta-dataset. The “Corrplot” R package was used to identify the potential HP regulatory genes in the meta-datasets. We used the “limma” R package to determine the differential genes between Hp-positive and Hp-negative patients in the GEO dataset (i.e. GSE6143 and GSE60662). The “upsetR” and “VennDiagram” R packages were employed to identify the overlapping genes. Then, the univariate Cox regression analysis was performed to identify HP-related prognostic genes, and the HR values of these genes were visualised using the “forestplot” R package.

### Evaluation of the clinical value of HP-related prognostic genes

Based on the HP-related prognostic genes, lasso regression and multivariate Cox regression were used to establish the prognostic risk model with the “survival” and “glmnet” R packages. Then, the samples were classified into high- and low-risk groups according to the median risk score. The “pheatmap”, “survival”, and “survminer” R packages were employed to demonstrate the difference in the prognosis between the high- and low-risk groups. The “scatterplot3d” R package was applied to investigate the distribution of patients with a different risk score.

### Nonnegative matrix factorisation

To evaluate the modification differences among the GC samples, we used the Nonnegative Matrix Factorisation (NMF) method to classify 482 GC patients from the meta-datasets based on the presence of HP-related prognostic genes. When the decreasing trend of the cophenetic correlation coefficient was most obvious, the k value was regarded as the best cluster number. The “NMF” R package was employed to plot the heatmap, basis components, and the connectivity matrix of NMF in different clusters.

### PPI network and functional pathway enrichment analysis

The protein–protein interaction network was constructed using the Search Tool for the Retrieval of Interacting Genes database (STRING, https://string-db.org/). Cytoscape software with the MCODE plugin was employed for the optical network and to identify the most significant module. The GO function annotation and the KEGG pathway enrichment analysis were performed using the “clusterProfiler” R package and DAVID (https://david-d.ncifcrf.gov/). The signal pathway gene sets were downloaded from MSigDB (https://www.gsea-msigdb.org/gsea/msigdb). Gene enrichment analysis was also performed using GSEA software (version 4.0).

### Generation of HP-related prognostic gene signature

To quantify the HP-related prognostic gene modification patterns in each sample, we defined the HPscore, a scoring system for evaluating individual GC patients. The principal component analysis (PCA) was performed to construct the HPscore. Similar to that described in previous studies (12, 13), we added PC1 and PC2 as the final gene signature scores. The HPscore was represented as

$$\mathrm{HPscore}=\sum_{i}^{j}(PC1i+PC2i)\;$$

The samples were categorised as high- and low-HPscore groups, with the optimal cutoff value. In addition, the distribution of patients with the HPscores was visualised using the t-distributed random neighbour embedding (T-SNE) method (“Rtsne” R package).

### Estimation of TME and stemness feature

The “xCell” R package was used to calculate the microenvironment score for the meta-dataset. In addition, the ESTIMATE was used to calculate tumour purity and the immune infiltration levels (14). Thus, a comprehensive microenvironment score that reflected tumour purity and immune cell infiltration in the tumour samples was constructed. According to the markers of immune cells obtain from the Charoentong's research (15), the single-sample gene-set enrichment analysis (ssGSEA) algorithm was employed to quantify the relative abundance of each immune cell infiltration in the GC tumour microenvironment by using the “GSVA” R package, and each immune cell infiltration score was standardised for further analyses. We also used the biological pathways constructed by Mariathasan et al. (16) to evaluate the association between the HPscore and biological processes, including (1) immune checkpoint; (2) antigen processing machinery (APM); (3) epithelial–mesenchymal transition (EMT) markers such as the EMT1, EMT2, and EMT3; (4) angiogenesis signature; (5) pan fibroblast TGF-b response signature (Pan-FTBRS); and (6) CD8+ T-effector signature. All the gene sets used in the study are listed in the Supplementary Table S6.

### Tissue samples and quantitative real-time polymerase chain reaction

A total of 24 tumor tissue and 20 normal adjacent tissue were collected from patients with GC. Following are the inclusion criteria for tissue specimens: (1) Diagnosis of GC from a pathological perspective; (2) Except for GC without other malignancies; (3) Surgical procedures are not preceded by radiotherapy or chemotherapy. The study was approved by the Ethics Committee and informed written consent was obtained from all patients. The specific experimental protocol for qRT-PCR referred to our previous research methods.

### Statistical analysis

All the data were processed using R 4.0.1 software. We obtained mutation data of the GC samples from the TCGA database. The “maftools” R package was employed to visualise mutation data. Independent prognostic factors were identified through the Cox analysis. The “limma” R package was employed to determine differentially expressed genes (DEGs) between the subgroups with fold change = 1, and the volcano map was used for visualisation. Survival curves were generated using the Kaplan–Meier method, and log-rank tests were performed to calculate the differences. The Sankey diagram was developed using the “networkD3” R package. The “ggplot2”, “ggpubr”, and “pheatmap” R packages were used to visualise the results. Pearson correlation coefficient among the data were calculated through the “Corrplot” R package and visualised using the “PerformanceAnalytics”, “Hmisc”, and “ggstatsplot” R packages. All statistical *P*-values were two-sided, and a *P* value of <0.05 was considered to be statistically significant. *P*-values: ns, *P* > 0.05; *P < 0.05; **P < 0.01; ***P < 0.001; ****P < 0.0001.

## Results

### The landscape of HP-related genes in GC

**Figure 2:**
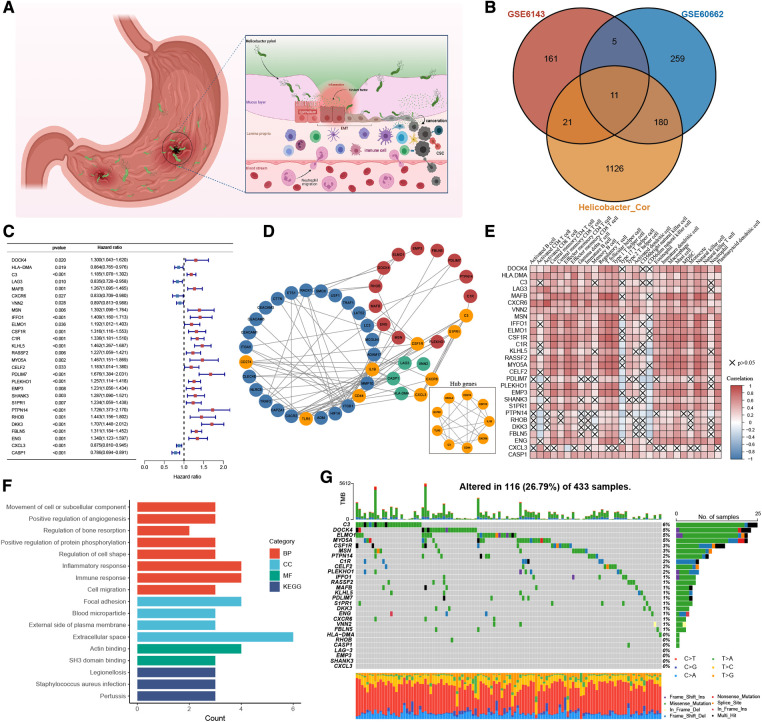
The landscape of HP-related prognostic genes in gastric cancer. (**A**) Pathogenesis of Helicobacter pylori. (**B**) Venny diagram displaying the overlap of differential gene expression profiles. (**C**) Blue and red represented the hazard ratio by the univariate cox regression model. (**D**) Protein-protein interaction (PPI) network (Blue nodes represent HP-related genes, Green and red are HP-related prognostic genes. Orange represents the hub genes identified by the mcode method). (**E**) Visualization of the correlation analysis results between HP-related prognostic genes and tumor-infiltrating immune cells in the meta-dataset. (**F**) GO and KEGG analysis results of 28 HP-related prognostic genes. (**G**) The mutation information of 28 HP-related prognostic genes was analyzed in the TCGA-STAD cohort.

Through systematic literature screening, a total of 39 genes were considered to be gastric infection HP gene signature (Supplementary Table S1), termed as HP-related genes. ROC results from three different datasets (GSE6143, GSE27411, and GSE60662) suggested that the HP-related gene sets can effectively diagnose HP infection (Supplementary Figure S1A). Figure 2A shows the dynamic carcinogenic process induced by HP infection in the stomach. To determine the best cluster dataset, we first performed the survival analysis to evaluate prognostic differences between the datasets (Supplementary Figure S1B). After excluding the datasets with poor data quality, GSE15459 and GSE66229 were integrated into training datasets (Supplementary Figures S1C,D), and TCGA-STAD and GSE84437 were used as testing datasets. Subsequently, the correlation analysis was performed to determine the correlation between the HP-related genes and meta-dataset, and a total of 1,338 genes were identified (Supplementary Table S2). The Venn plot was used to show the overlapping region between 1,338 genes and the differential genes in GSE6143 and GSE60662. A total of 217 genes were confirmed for follow-up analysis (Figure 2B and Supplementary Figure S2A). To further determine the relationship between HP infection and GC, the univariate Cox regression model showed that 28 genes were associated with GC prognosis (Figure 2C), and these genes were termed as HP-related prognostic genes. A protein interaction network of 21 HP-related prognostic genes and the HP-related genes was constructed. The mcode plugin was used to identify potential hub genes (Figure 2D). The strong correlation among the HP-related prognostic genes is shown in Supplementary Figures S2B. In addition, Spearman correlation analysis showed a robust correlation between HP-related prognostic genes and tumour immune-infiltrating cells (Figure 2E). Type 17 helper T cells and CD56 dim natural killer cells were negatively correlated with poor prognostic genes but positively correlated with favourable prognostic genes. Subsequent KEGG and GO enrichment analyses also confirmed the strong correlation between these prognostic genes and the immune signal pathway. For example, the “inflammatory response” and “immune response” signalling pathways of GO were enriched. In addition, the enrichment of “positive regulation of angiogenesis” and “focal adhesion” and “cell migration” signalling pathways indicated the potential biological function of 28 prognostic genes in GC (Figure 2F). We studied the mutation incidence in TCGA-STAD patients to fully describe the characteristics of HP prognostic genes (Figure 2G). Of the 433 samples, 116 harboured a mutation in HP-related prognostic genes, with a frequency of 26.79%. *C3*, *DOCK4* and *ELMO1* had the highest mutation rate (6%), followed by *MYO5A* (5%). These results suggested that the HP-related prognostic genes are strongly associated with the immune microenvironment of GC and tumour progression.

### Risk stratification of patients with gastric cancer based on HP-related prognostic genes

**Figure 3:**
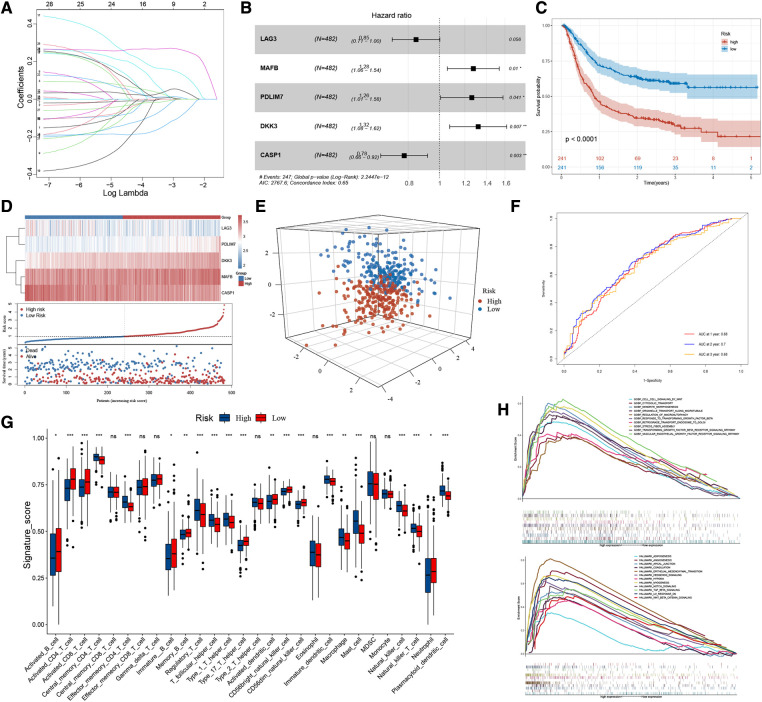
The workflow of risk-score model construction. (**A**) LASSO model coefficients. (**B**) *P < 0.05 in multivariable Cox proportional hazards model. (**C**) Kaplan-Meier survival curve of the OS in the high- and low-risk groups (meta-dataset). (**D**) From top to bottom are five prognostic signature RNAs expression heatmap, the risk score, and patients’ survival status distribution between low- and high-risk groups. (**E**) Principal component analysis (PCA) shows the difference between the high-risk and low-risk groups based on the risk score. (**F**) ROC curves for 1-year, 2-year and 3-year overall survival, with AUC = 0.68 , 0.70 and 0.66 respectively. (**G**) The box plot results suggest that tumor-infiltrating immune cells were significantly differently distributed in the high-risk and low-risk groups. (**H**) GSEA results show the relevant signaling pathways involved in the high-risk group.

We performed lasso regression of 28 genes based on meta-dataset, and the results suggested that *LAG3*, *MAFB*, *PDLIM7*, *DKK3*, and *CASP1* can be used to establish risk models (Figure 3A). Multivariate Cox analysis was then used to calculate the risk score of each sample in the meta-dataset. The forest plot showed the relationship between the five genes and cancer prognosis (Figure 3B). Then, GC patients were divided into high- and low-risk groups, with the median risk score as the threshold. Survival analysis showed that the survival time of the high-risk group was significantly lower than that of the low-risk group (Figure 3C, *P* < 0.0001). The heatmap result showed that *MAFB*, *PDLIM7*, and *DKK3* were highly expressed in the high-risk group but *LAG3* and *CASP1* were not expressed. With the increase in the risk score, the proportion of death in patients increased significantly (Figure 3D). Principal component analysis showed significant differences in the high- and low-risk cohorts (Figure 3E). The area under the curve (AUC) of the meta-datasets at 1, 2, and 3years were 0.68, 0.70, and 0.68 (Figure 3F). The datasets GSE29272 and GSE84437 were used to evaluate the actual value of the risk model of the high- and low-risk cohorts, respectively. These results suggested that our model can well stratify the risk of GC, and a significant difference was observed in the survival of GC patients between the high- and low-risk groups (Supplementary Figure S3A). As shown in Figure 3G, the high-risk group had a high levels of regulatory T cells, T follicular helper cells, type 1 T helper cells, mast cells, and plasmacytoid dendritic cell infiltration in the tumour rather than activated CD4 T cells, activated CD8 T cells, type 17 helper T cells, and CD56 light/dim natural killer cells. We used GSEA to analyse the enrichment level of the pathways. The hallmark and GO enrichment pathways, including Wnt, autophagy, TGF-B, EMT, Angiogenesis, Hypoxia, Notch, and Hedgehog signalling pathways were of considerable attention (Figure 3H) because the participation and imbalance of these pathways might be the reasons for the poor prognosis in the high-risk group. Significant differences were found in HP-related gene expression between high- and low-risk groups (Supplementary Figure S3B).

To summarise, the risk model based on 28 HP-related prognostic genes can be used as an essential index to evaluate the prognosis of GC. At the same time, multiple tumour-related signal pathways were enriched. Significant differences were found in the expression of HP-related genes and the distribution of infiltrating immune cells among the two risk groups.

### Different modification patterns of HP-related prognostic genes

**Figure 4:**
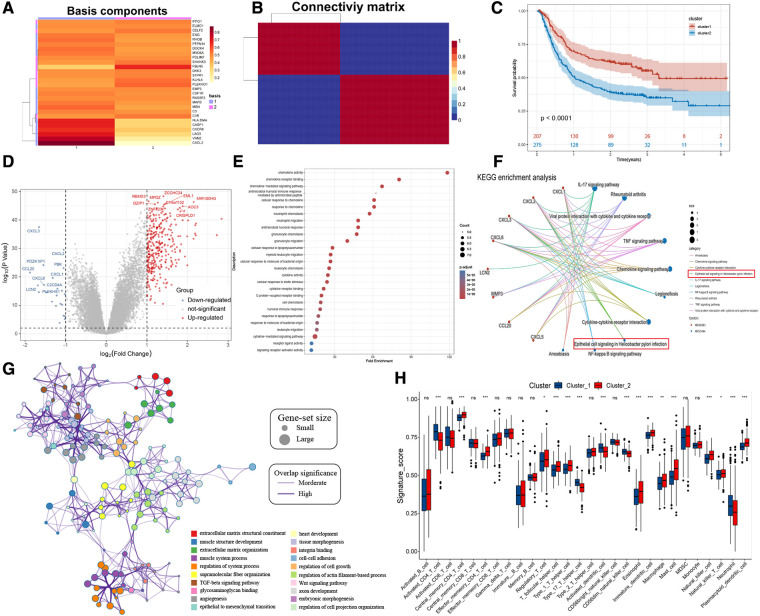
NMF for HP-related prognostic genes modification patterns, biological processes, and immune cell infiltration analysis. (**A**) NMF heatmap of basic components of HP-related prognostic genes expression in the two clusters. (**B**) Connectivity matrix for patients with gastric cancer in the meta-dataset by NMF. (**C**) Kaplan-Meier curves showing survival differences between two clusters. (**D**) Volcano plot shows the different genes between cluster 1 and cluster 2. (**E,F**) Bubble plot and gene-concerpt network shows up-regulated genes in cluster 1 involved signaling pathways. (**G**) Cytoscape and enrichment maps are used to visualize the function enrichment analysis results of up-regulated genes in cluster 2 involved signaling pathways. (**H**) Box plot results show differences in the distribution of infiltrating immune cells in different clusters.

The risk stratification of the population was successfully performed by building the risk model. We then classified the patients based on meta-dataset by using the NMF method, calculated the NMF symbiotic correlation coefficient, and selected *k* = 2 as the best grouping value (Supplementary Figures S4A,B). We successfully obtained two different modification patterns of HP-related prognostic genes in patients with GC, termed cluster_1 and cluster_2 (Figures 4A,B). Significant differences were observed in the survival between cluster_1 and cluster_2 (Figure 4C, *P* < 0.0001). A total of 338 DEGs were identified in the two HP-related prognostic gene modification groups (Figure 4D and Supplementary Table S3). The clusterProfiler R package was used to identify the function and signalling pathways of differential genes. The results showed that bacterial invasion- and inflammation-related pathways were enriched in cluster_1, indicating favourable prognosis (Figures 4E,F). The enrichment pathways of cluster_2 were mainly extracellular matrix- and membrane protein receptor-related signaling pathways (Figure 4G). The distribution of infiltrating immune cells in cluster_1 and cluster_2 was different (Figure 4H). Specifically, cluster_1 was rich in the infiltrated cells involved in inflammatory stress, such as activated CD4 T cells, type 17 helper T cells, activated dendritic cells, CD56 dim natural killer cells, and neutrophils. In contrast, cluster_2 showed infiltration of adaptive immune cells including regulatory T cells, T follicular helper cells, type 1 T helper cells, macrophages, mast cells, and plasmacytoid dendritic cells. Importantly, the epithelial cell signalling pathway in HP infection was enriched in cluster_1. Subsequently, we verified the expression of HP-related genes between cluster_1 and cluster_2 (Supplementary Figure S4C). These results directly confirm the reliability of the HP-related prognostic gene. A total of 28 HP-related prognostic genes can accurately classify the population of GC patients, and a significant difference was observed in tumour progression between cluster_1 and cluster_2.

### Generation of a HP-related prognostic gene signature

**Figure 5:**
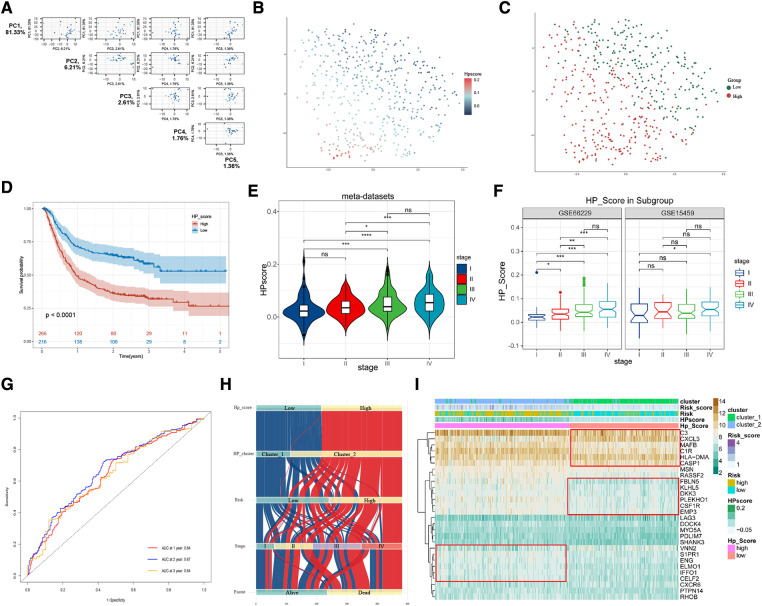
Construction of HPscore system and verification of system stability. (**A**) Pairs plot showing the results of principal component analysis (PCA) base on 28 HP-related prognostic genes. (**B,C**) The T-SNE of HPscore and Hp_Score groups for all samples. (**D**) Overall survival analysis verified HPscore system in meta-dataset. (**E,F**) The differences of HPscore in gastric cancer stages were observed in the meta-dataset and internal datasets, respectively. (**G**) ROC curves for 1-year, 2-year and 3-year overall survival, with AUC = 0.64, 0.67 and 0.64 respectively. (**H**) The Sankey map of samples in HP_score group, risk group, clusters group, stages, and survival outcome. (**I**) Heatmap of the expression levels of 28 HP-related prognostic genes in different groups.

To further explore the biological differences in HP-related prognostic genes among individual GC samples, we constructed an HPscore system by employing the PCA method based on meta-datasets (Figure 5A and Supplementary Figure S5A). The T-SNE algorithm was used to visualise the sample HPscore (Figure 5B,C), and the results showed an apparent distance gradient among the GC samples with the increase in the HPscore. The meta-dataset was divided into two groups based on the optimal cutoff value: the high Hp_Score group (*n* = 212) and the low Hp_Score group (*n* = 256). Similar to the risk model, the high Hp_Score group demonstrated a shorter survival time than the low Hp_Score group (Figure 5D, *P* < 0.00001). To assess the stability and expansibility of the scoring system, the HPscores between the internal datasets GSE15459 and GSE66229 were compared, and no significant difference was observed between the two datasets (Supplementary Figure S5B). External datasets GSE29272 (GPL96) and GSE84437 (GPL6947) were used for the verification of the survival analysis, and the results are shown in Supplementary Figure S5C (*P* = 0.004) and S5D (*P* = 0.009). We then summarised the clinical information in meta-datasets to verify the relationship between the HPscore and clinical features (Supplementary Table S4). The results suggested that the HPscore increased with the increase in the TNM stage of cancer (Figure 5E); similar results were obtained through internal grouping (Figure 5F). We then analysed differences in the HPscores between the high- and low-risk groups and between cluster_1 and cluster_2 (Supplementary Figures S5E,F). The area under the curve (AUC) of the meta-datasets at 1, 2, and 3years were 0.64, 0.67, and 0.64 (Figure 5G), respectively. The alluvial diagram shows the flow of modified samples with different HPscores in a risk group, cluster group, stage cluster, and survival cluster (Figure 5H). We performed unsupervised cluster analysis based on 28 HP-related prognostic genes to determine the relationship between different GC subgroups (Figure 5I). The results showed a high degree of consistency between HPscore, Risk, and Cluster, and significant differences in the expression of three gene subgroups between the groups.

### Relationship between the HPscore and immune microenvironment

**Figure 6:**
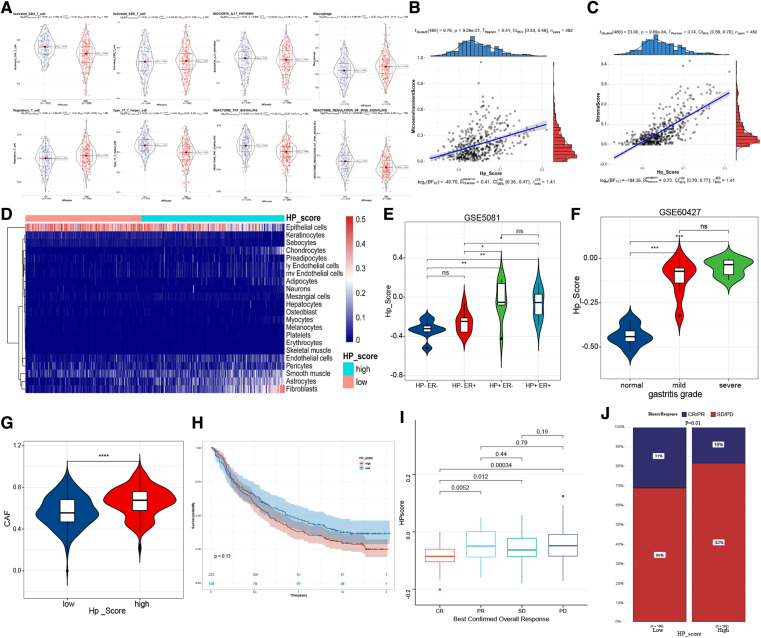
The immune-related characteristics of HPscore. (**A**) Significant differences of immune cell infiltration between high-HPscore and low-HPscore groups. (**B,C**) A scatter plot of the positive relationship between HPscore and Stromastore and Microenvironmentscore. (**D**) The heatmap shows the different distribution of stromal cells between both groups. (**E,F**) The significant differences of HPscore in patients with different levels of HP infection. (**G**) Relative distribution of CAF cells in HPscore high vs. low subgroups. (**H**) Kaplan-Meier curves for high and low HPscore patient groups receiving anti-PDL1 treatments. (**I,J**) The fraction of patients with clinical response to anti-PDL1 immunotherapy in low or high HPscore groups.

To confirm the relationship between HPscore and immune infiltration, we scored the samples by using the ssGSEA and xCell method. Activated CD4 T cells, type 17 T helper cells, TNF signalling, and IFNA signalling were mainly enriched in the low Hp_Score, whereas regulatory T cells and macrophages were significantly increased in the high Hp_Score (Figure 6A). The correlation analysis showed that the HPscore was significantly and positively correlated with the matrix cell score (*r* = 0.74, *P* < 0.0001) (Figure 6B) and microenvironment score (*r* = 0.41, *P* < 0.001) (Figure 6C). The comprehensive landscape of stromal cells in the high- and low-Hp score groups is shown in the heatmap (Figure 6D). The number of epithelial cells in the high Hp_Score group was found to be significantly lower than that in the low Hp_Score group, whereas the number of fibroblasts and endothelial cells increased significantly in the high HP group. To study the relationship between the HPscore and inflammation, we first verified the relationship between the HPscore and Hp infection in the GSE5081 dataset. Regardless of inflammation, the Hp-positive group had a higher HPscore than the Hp-negative group (Figure 6E). In the dataset of inflammation induced by Hp infection (GSE60427), the HPscore better reflected the level of inflammation (Figure 6F, *P* < 0.001). To evaluate the relationship between the HPscore and cancer-related fibroblasts (CAF), we scored meta-dataset samples based on CAF cell characteristic genes. The results showed that the CAF enrichment score in the high Hp_Score group was significantly higher than that in the low Hp_Score group (Figure 6G, *P* < 0.00001).

Immunotherapy is a significant breakthrough in tumour therapy. We further explored the relationship between the HPscore and immunotherapy in the immunotherapy cohorts IMvigor210 (Supplementary Table S5). We found that the survival rate of patients with a high Hp_Score was lower than that of patients with a low Hp_Score, and the response to treatment was worse in the high Hp_Score group (Figures 6H–J).

### Analysis of the relationship between the HPscore and tumour stemness of GC

**Figure 7:**
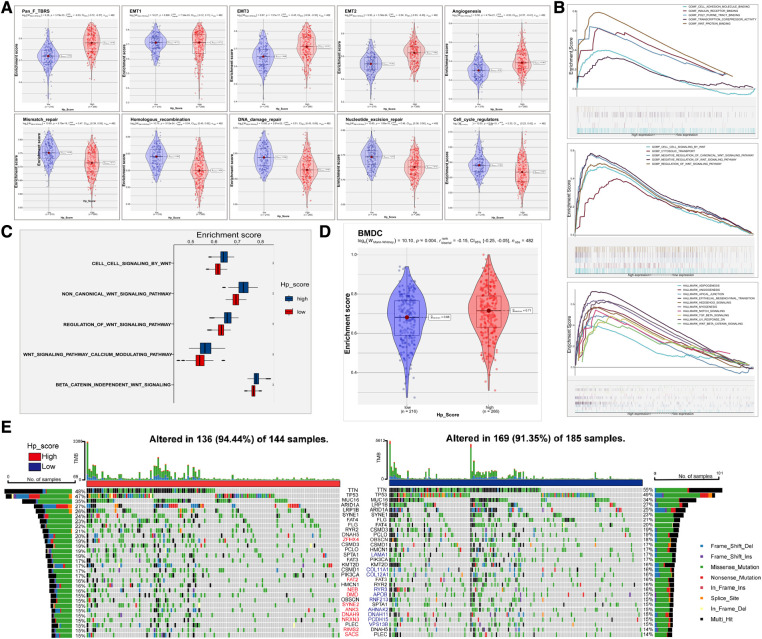
Clinical features and tumor stemness signature of HPscore. (**A**) Boxplot for the significant differences of the current immune-related signatures between two HP_score groups. (**B**) Prediction of HPscore-related pathways and molecular function by enrichment plots from GO analysis and hallmark enrichment pathway. (**C**) Differences enrichment scores of Wnt pathway between two HP_score groups. (**D**) Boxplot for the significant differences of BMDC enrichment scores between the two HP_score groups. (**E**) The landscape of tumor somatic mutation between the two HP_score groups.

The study of the biological processes of tumor progression related to the HPscore showed that the HPscore was positively correlated with EMT2, EMT3, Pan_f_TBRS, and angiogenesis in GC but negatively correlated with DNA damage repair, mismatch repair, homologous recombination, nucleotide excision repair, and cell cycle regulators (Figure 7A). GSEA functional enrichment analysis suggested that EMT, Angiogenesis, cell adhesion, extracellular matrix junction, Wnt, TGF-b, Hedgehog, and Notch pathway were widely enriched in the high Hp_Score group (Figure 7B). Subsequently, we compared the performance of gene sets related to the Wnt pathway in the HPscore subgroup. The enrichment score of the high Hp_Score group was higher than that of the low Hp_Score group (Figure 7C). Similar results were obtained using BMDC enrichment scores (Figure 7D). Finally, the somatic mutation map in the TCGA cohort showed no significant difference in the mutation rates of *TP53*, *TTN*, and other top 30 genes between the high Hp_Score group and the low Hp_Score group (Figure 7E).

### Validation of HP-related prognostic genes

**Figure 8:**
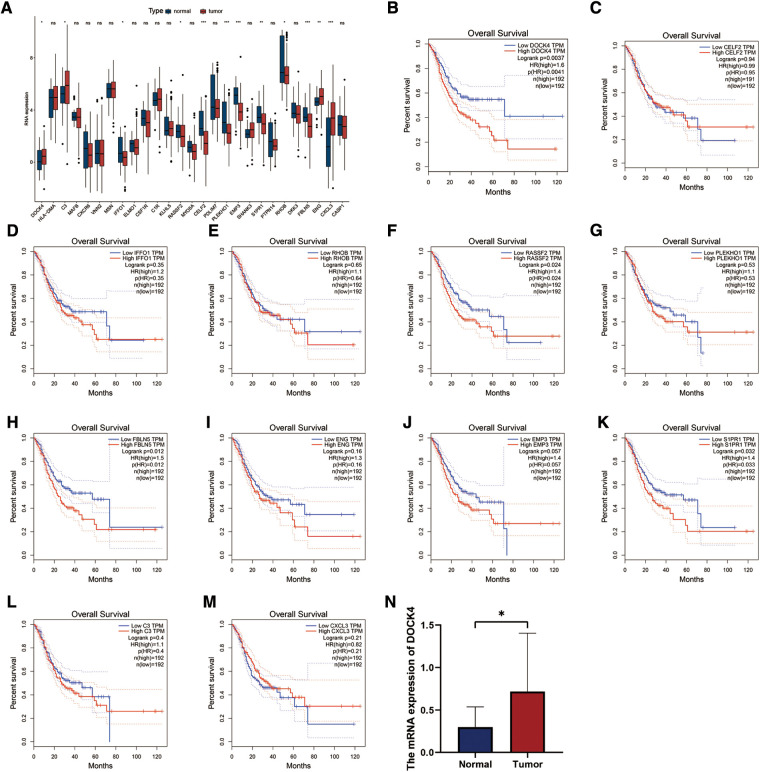
Expression and validation of 28 HP-related prognostic genes. (**A**) The expression patterns of 28 HP-related prognostic genes were analyzed in the TCGA-STAD cohort. (**B–M**) Analysis of overall survival with HP genes high and low expression groups in TCGA-STAD cohort. (**N**) qRT-PCR analysis of the expression of DOCK4 in 29 normal and 24 tumor tissue samples.

The TCGA-STAD dataset was used to verify the expression of 28 HP-related prognostic genes. The results showed that DOCK4, C3, ENG, and CXCL3 were highly expressed in patients with TCGA-STAD tumours, whereas IFFO1, RASSF2, CELF2, PLEKHO1, EMP3, S1PR1, RHOB, FBLN5, and CASP1were highly expressed in healthy individuals (Figure 8A). Survival analysis in TCGA-STAD cohort show that the patients with high expression of DOCK4, RASSF, FBLNF or S1PR1 had poorer overall survival (Figures 8B–M, *P* < 0.05). The results of qRT-PCR indicated that the mRNA level of DOCK4 was higher in carcinoma tissues than those in normal tissues (Figure 8N, *P* = 0.0126).

## Discussion

HP infection is the most common risk factor for GC. The virulence factors produced by HP affect the signal transmission between cells, and chronic infection of gastric mucosa leads to changes in the local microenvironment. Most people infected with HP do not show symptoms related to bacterial virulence, host genetic polymorphism, and environmental factors (1). First, we identified the genetic markers associated with HP infection in patients with gastropathy by searching for HP-related literature. Because of the lack of sample data on large-scale carcinogenesis caused by HP infection, we used the correlation analysis to identify the gene modification phenotype of HP-related genes in GC. By performing systematic analysis, 28 genes were identified for follow-up research. By constructing a prognostic risk model and through NMF grouping, we identified two clinical tags of 28 genes: “prognostic indicators” and “HP infection associated.” Based on the results, we considered 28 genes as HP-related prognostic genes in GC. Then, we created an HPscore system to quantify the modification characteristics of these 28 HP-related prognostic genes in the samples and determined the accuracy and stability of the HPscore system by using external datasets. After stringent verification, we concluded that the HPscore system can accurately reflect the status of HP infection and survival outcome in patients with GC. To confirm our inference, we successfully divided the patients with GC into two subgroups based on differences in the HPscore. Differences in the survival outcomes and HP infection status between subgroups were significant, which increased our confidence in continuing using HPscore to explore the detailed mechanisms of HP pathogenesis.

To the best of our knowledge, this study is the first to determine the relationship between HP infection and the 39 genetic markers identified through the ROC curve analysis. Our results showed that the areas under the ROC curve of the three datasets were >0.7, indicating that the 39 gene sets could accurately reflect the status of HP infection. To further explore the role of HP infection in GC, we performed correlation analysis to identify potential regulatory genes of HP in GC based on the meta-dataset. To improve the association between these genes and HP, we used HP+ and HP− differential gene datasets to screen the regulatory gene sets used in the previous step. Subsequently, the survival data were introduced into the analysis and by using univariate Cox analysis, we identified 28 genes for follow-up research. The results of the protein interaction network suggested that *C3*, *CSFR*, *S1PR1*, *CXCR6*, and *CXCL3* could be primarily involved in HP pathogenesis. HP cytotoxin-associated gene A (CagA) has been reported to relieve the inhibitory effect of *TGF- β* on *CXCL3* and aggravate the inflammatory response (17). Studies have reported that *S1PR1* is associated with the differentiation of memory T cells (18–20) and affects the prognosis of GC by promoting chemotherapy resistance (21, 22). As expected, the 28 genes were strongly associated with tumour immune-infiltrating cells. In addition, *C3* (23), *CSFR* (24–26), *CXCR6* (27–29), and *CXCL3* (30) have been identified as immune-related factors, and their misexpression in GC affects prognosis (24, 29, 31, 32). Our GO and KEGG analysis results also suggested that these genes are involved in immunoregulation and tumour progression pathways. Then, we used the TCGA-STAD dataset to evaluate the expression of these 28 genes in benign and malignant tissues and their mutations in tumour samples. However, the results of this analysis could not provide valuable insights. Therefore, to further explore the relationship between these 28 genes and the prognosis of GC, we selected five of them to construct a prognostic risk model based on multivariate Cox analysis. Risk prediction models based on polygenic characteristics are commonly used to predict survival outcomes of patients with cancer (33–35). Our prognostic model showed that the expressions of *LAG3* and *CASP1* were negatively correlated with poor prognosis in patients with GC. *LAG3* inhibits the growth of GC and promotes the secretion of CD8+ T cells, *IL-12*, and *IFN- γ* (36), and the expression of *LAG-3* on T-cell surface can be used as a reasonable biomarker of anti-PD-1 therapy (37, 38). In addition, *CASP1* has been shown to be activated by HP infection (39, 40). It has both pro-inflammatory and anti-inflammatory effects because of its different substrates (41). The expression of the other three genes, namely *MAFB*, *DKK3*, and *PDLIM7*, was positively correlated with the poor prognosis of patients with GC. Our results suggested that the risk model based on 28 genes can separate the population and exhibits a superior performance in predicting the prognosis of patients with GC. While analysing the difference in infiltrating immune cells between the high- and low-risk groups, our results suggested that the activated CD4+ T cells and CD8+ T cells, rather than regulatory T cells, are highly enriched in the low-risk groups, which is consistent with the molecular function of *LAG3* and *CASP1*. To understand the underlying mechanism of poor prognosis in high-risk populations, the GSEA was used to identify significantly enriched signalling pathways in the high-risk populations. The results suggested that the high-risk group were enriched in the angiogenesis, hypoxia, macrophage autophagy, and tumour stem cell-related signalling pathway. A report showed that the expression of *MAFB* oncoprotein is regulated by the cytolethal distending toxin of enterohepatic HP (42), and *MAFB* is specifically expressed in tumour-associated macrophages to induce angiogenesis (43). In addition, studies on osteosarcoma have reported that *MAFB* increases the expression of stem cell regulatory factor *SOX9* at the transcriptional level (44). Overall, the activation of carcinogenic pathways induced by misexpression of 28 genes is the cause of poor prognosis in the high-risk group. Here, we identified the first clinical tag of 28 genes: prognostic indicators.

To observe differences among the samples with different modified states of 28 genes, we further divided the patients with GC into two by using the NMF method, namely cluster_1 and cluster_2. The survival analysis suggested a significant difference in survival between the two groups of patients with GC. The difference analysis showed that cluster_1 with favourable prognosis had a higher expression of immune-related factors, such as *CCL20*, *CXCL2*, and *CXCL3*, and is supported by the signalling pathway analysis. Cytokines, chemokines, and inflammatory response-related signalling pathways were widely enriched in cluster_1. In addition, the enrichment of HP signalling pathway was observed in cluster_1, suggesting that cluster_1 is closer to the state of inflammatory response in the early stage of HP infection. To prove this result, we compared the distribution of infiltrating immune cells between cluster_1 and cluster_2. The results were similar to those observed in the case of high- and low-risk groups. Activated CD4+ T cells, type 17 helper T cells, and neutrophils were highly enriched in cluster_1. This result supported that HP-induced diseases are mainly mediated by Th1 cells and Th1 cytokines (3). In addition, TH17 helper cells fight against the immune response of extracellular bacteria and moulds, and the cytokines released by the helper cells mainly activate neutrophils (45). and are highly consistent with our results. Combined with the aforementioned results, we identified the second clinical tag of 28 genes: HP infection-related feature. Subsequently, we re-verified the difference in the expression of HP prognosis-related genes between cluster_1 and cluster_2, which suggested that *HLA-DMA*, *CASP1*, *CXCR6*, *LAG3*, *VNN2*, and *CXCL3* were highly expressed in cluster_1. *VNN2* is a haematopoietic stem cell marker (46, 47), that participates in inflammation and leukocyte migration (48). However, the role of *VNN2* in GC is unclear. Based on the aforementioned results, we defined these 28 genes as HP-related prognostic genes.

To evaluate the modification patterns of HP-related prognostic genes in a single sample, we established a scoring system based on 28 HP-related prognostic genes and termed it as the HPscore. Comprehensive analysis showed that the HPscore is related to tumour progression and affects tumour prognosis. HP infection leads to the imbalance of DNA methylation in gastric mucosal epithelial cells of the host (49–52). As a result, some proto-oncogenes are activated to induce cancer (53). Microsatellite instability (MSI) in GC also showed specific hypermethylation of DNA (54). Surprisingly, a negative correlation was observed between the HPscore and DNA methylation stemness index and mutation load in TCGA datasets. After optimising the DNA methylation index, the HPscore became unrelated to the DNA methylation level (the results are not shown). In terms of clinical features, MSI was also not related to the HPscore (the results are not provided). The reason may be that in the TCGA-STAD dataset, mDNAsi derived from the one-class logistic regression machine learning algorithm (OCLR) does not sufficiently reflect the methylation level of GC (the high level of tumour cell stemness index in this study is a protective factor for GC prognosis). As reported previously, GC may have multiple stem cell-like genomic characteristics or non-stem phenotypes dominated by hypermethylation (55). Excitingly, we compared the differences in nucleotide_excision_repair, DNA_damage_repair, homologous_recombination, mismatch_repair, and cell_cycle_regulators between the high- and low-score arrays, and the results confirmed our HPscore system. In the high-score group, the ability to repair DNA damage was generally low, suggesting that HP infection impairs the autonomous repair function of cells (56). In addition, the two HPscore groups showed different TME permeation characteristics. The low-score group showed a stronger inflammatory response, whereas the high-score group was accompanied by a large number of stromal cells including fibroblasts and endothelial cells. The subsequent results showed that the enrichment score of CAF markers in the high-HPscore group was higher than that in the low-HPscore group. However, no difference was observed in the HPscores between normal fibroblasts and tumour-associated fibroblasts, suggesting that the modification of HP-related prognostic genes in tumour cells induces the transformation of NF to CAF rather than to fibrous cells. In chronic inflammation and cancer, tissue-resident fibroblasts become the critical cell types that regulate the activation or inhibition of the immune response (11). Fibroblasts assist immune cells to maintain an effective inflammatory environment in chronic inflammation and promote immunosuppression in malignant tumours to assist tumour cells in immune escape (10, 11). In addition, fibroblasts are necessary for the synthesis and remodelling of the extracellular matrix during angiogenesis and germination (57). These new blood vessels bring bone marrow-derived suppressor cells, including BMDCs, into the TME. Chronic HP infection can lead to BMDC recruitment to promote the stemness-like characteristics of GC cells (5, 58). Our results also supported this conclusion. GSEA results suggested that various tumour stem cell-related signalling pathways, such as the Notch signal pathway, Wnt pathway, and Hedgehog pathway, were enriched in the high-score group. BMDCs associated with HP were also significantly enriched in the high-score group. In addition, our study suggested that the Wnt pathway plays a key role in the carcinogenesis induced by HP infection. Studies have reported that HP promotes tumour progression by activating the Wnt/β-catenin pathway (59, 60) and promotes CSC-like characteristics in GC cells (61); these findings are consistent with those of our study. After optimising the tumour stem cell index, we found that the HPscore was positively correlated with the tumour stemness index. Simultaneously, we also proved the positive correlation between the HPscore and EMT and F-TGF-B. These results suggested that HP helps tumour immune escape and angiogenesis by activating fibroblasts and recruits BMDCs to enhance the characteristics of GC stem cells and promote cancer development, in which the Wnt signalling pathway plays a key role.

Combined with the aforementioned evidence, we studied the role of HPscore in treatment. We first evaluated the relationship between the HPscore and PD-L1. Unfortunately, the predictive value of the HPscore in PD1 and anti-PD-L1 immunotherapy is unstable. To date, no detailed report on the relationship between HP infection and PD1/PD-L1 is available. Because of the complexity of the TME, only a few patients benefit from the treatment of immune checkpoint block (62). Our HPscore may not perform well in diseases that are not related to bacterial infections. Hence, more experiments are needed to verify the interaction between HP and PD-L1. We then predicted the therapeutic efficacy of antimicrobials by using the HPscore. Based on the limited data, we found that the HPscore was positively correlated with the degree of gastritis, which can help predict the grade of gastritis. Metronidazole is used to treat various infectious diseases including HP infection. Studies have reported that the sensitivity to metronidazole decreases in patients with HP infection (63, 64). In the vaginitis data set, the HPscore decreased significantly after three weeks of metronidazole treatment. These results showed that the HPscore plays a guiding role in clinical diagnosis and efficacy evaluation. However, our study has many limitations. First, because of the complexity of HP pathogenesis, the existing HP metadata could not fully reflect the status of HP infection. Second, we found a stronger correlation of HP infection with stromal cells than with the infiltrating immune cells. This result indicated that more communication might exist between stromal cells and HP. Finally, the mechanism through which HP recruits BMDCs remains to elucidated experimentally in detail.

## Conclusions

In conclusion, the HPscore can comprehensively evaluate the permeability characteristics of the individual TME and drug efficacy in patients with GC. In this study, we used HP-related gene datasets to derive the characteristics of HP-related prognostic genes for the first time. Based on the HPscore system, we showed the comprehensive view of the TME of the sample shaped by HP-related prognostic gene modification. Clinically, the HPscore can predict the inflammatory grade of patients with gastritis and reflect the therapeutic effect of metronidazole. Our findings provide a basis and framework for better understanding the carcinogenic mechanism in patients infected with HP and develop an efficient tool for personalised and effective treatment strategies.

## Data Availability

The datasets presented in this study can be found in online repositories. The names of the repository/repositories and accession number(s) can be found in the article/**Supplementary Material**.
