# Supplementary material for: Integrated analysis of Helicobacter pylori-related prognostic gene modification patterns in the tumour microenvironment of gastric cancer
Source: Front Surg. 2022 Sep 30;9:964203. doi: 10.3389/fsurg.2022.964203 (PMC9561901; doi:10.3389/fsurg.2022.964203)
Supplement: Supplementary file 1 [file Table1.pdf]

Integrated analysis of Helicobacter pylori-related prognostic gene modification patterns in the tumour microenvironment of gastric cancer

Kaitian Zheng[1#], Ye Wang[1#], Jiancheng Wang[1], Congjun Wang[1], Junqiang Chen[2*]

1. Guangxi Key Laboratory of Enhanced Recovery after Surgery for Gastrointestinal Cancer, Guangxi Medical University, No.22, Shuangyong Road, Qingxiu District, Nanning City, Guangxi Zhuang Autonomous Region, China.
2. Guangxi Key Laboratory of Enhanced Recovery after Surgery for Gastrointestinal Cancer, Department of Gastrointestinal Gland Surgery, The First Affiliated Hospital of Guangxi Medical University, Nanning City, Guangxi Zhuang Autonomous Region, 530021, China.

# Ye Wang and Kaitian Zheng contributed equally to this work.

*** Correspondence:**

Junqiang Chen

chenjunqiang@gxmu.edu.cn

SUMMARY
Table S1. Summary results of HP-related gene markers
Table S2. The 1338 genes were identified between the HP-related genes and meta-dataset.
Table S3. DEGs of HP-related prognostic gene modification groups.
Table S4. The detailed information of HP-related groups for the patients in the meta-cohort.
Table S5. Clinical information and treatment information for patients in IMvigor210
Table S6. Gene Signatures enrolled in this study.

Table.1 Summary results of HP-related gene markers

Year	Genes	Title	Journal	DOI
2019	ATP4A<br>ADAM17<br>α5β1<br>IL-1β	Gastric Parietal Cell Physiology and Helicobacter pylori–Induced Disease	Gastroenterology	10.1053/j.gastro.2019.02.036
2021	HPSE(heparanase)	Helicobacter pylori-Induced Heparanase Promotes Colonization and Gastritis	Frontiers in immunology	10.3389/fimmu.2021.675747
2019	TRPML1	VacA generates a protective intracellular reservoir for Helicobacter pylori that is eliminated by activation of the lysosomal calcium channel TRPML1	Nature microbiology	10.1038/s41564-019-0441-6
2021	CD44<br>LC3	Autophagy induced by Helicobacter pylori infection is necessary for gastric cancer stem cell emergence	Gastric cancer	10.1007/s10120-020-01118-9
2021	PRTG(Protogenin)	The novel ZEB1-upregulated protein PRTG induced by Helicobacter pylori infection promotes gastric carcinogenesis through the cGMP/PKG signaling pathway	Cell death & disease	10.1038/s41419-021-03440-1
2020	SMOX(spermine oxidase)	Spermine oxidase mediates Helicobacter pylori-induced gastric inflammation, DNA damage, and carcinogenic signaling	Oncogene	10.1038/s41388-020-1304-6
2020	USF1	USF1 defect drives p53 degradation during Helicobacter pylori infection and accelerates gastric carcinogenesis	Gut	10.1136/gutjnl-2019-318640
2019	MMP-10	Helicobacter pylori-induced matrix metallopeptidase-10 promotes gastric bacterial colonization and gastritis	Science advances	10.1126/sciadv.aau6547
2019	HIF-1α	The Helicobacter pylori Urease Virulence Factor Is Required for the Induction of Hypoxia-Induced Factor-1α in	Cancers	10.3390/cancers11060799

		Gastric Cells		
2019	TRAF1<br>TRAF2	Helicobacter pyloriInhibitory Effect of β-Carotene on -Induced TRAF Expression and Hyper-Proliferation in Gastric Epithelial Cells	Antioxidants (Basel, Switzerland)	10.3390/antiox8120637
2019	LC3	A Novel Role for Helicobacter pylori Gamma-Glutamyltranspeptidase in Regulating Autophagy and Bacterial Internalization in Human Gastric Cells	Cancers	10.3390/cancers11060801
2020	GKN1	Women with chronic follicular gastritis positive for Helicobacter pylori express lower levels of GKN1	Gastric cancer	10.1007/s10120-020-01049-5
2020	MCOLN1	MCOLN1/TRPML1 inhibition - a novel strategy used by Helicobacter pylori to escape autophagic killing and antibiotic eradication therapy in vivo	Autophagy	10.1080/15548627.2019.1677322
2020	CTTN(Cortactin)	Cortactin: A Major Cellular T arget of the Gastric Carcinogen Helicobacter pylori	Cancers	10.3390/cancers12010159
2021	MRCKβ	Helicobacter pylori-induced gastric cancer is orchestrated by MRCKβ-mediated Siah2 phosphorylation	Journal of biomedical science	10.1186/s12929-021-00710-0
2019	PD-L1(CD274)	Increased Programmed Death-Ligand 1 is an Early Epithelial Cell Response to Helicobacter pylori Infection	PLoS pathogens	10.1371/journal.ppat.1007468
2019	CAPZA1	CAPZA1 determines the risk of gastric carcinogenesis by inhibiting Helicobacter pylori CagA-degraded autophagy	Autophagy	10.1080/15548627.2018.1515530
2019	PDIA3	Vitamin D3 activates the autolysosomal degradation function against Helicobacter pylori through the PDIA3 receptor in gastric epithelial cells	Autophagy	10.1080/15548627.2018.1557835
2020	HIF-1α	Helicobacter pyloriThe Degree of Infection Affects the State of	Oxidative medicine and	10.1155/2020/5281795

		Macrophage Polarization through Crosstalk between ROS and HIF-1	cellular longevity	
2019	IL-17RB	Decreased IL-17RB expression impairs CD11bCD11c myeloid cell accumulation in gastric mucosa and host defense during the early-phase of Helicobacter pylori infection	Cell death & disease	10.1038/s41419-019-1312-z
2019	TRAP1	Helicobacter pylori-downregulated tumor necrosis factor receptor-associated protein 1 mediates apoptosis of human gastric epithelial cells	Journal of cellular physiology	10.1002/jcp.28223
2020	ETS1	Expression of ETS1 in gastric epithelial cells positively regulate inflammatory response in Helicobacter pylori-associated gastritis		10.1038/s41419-020-2705-8
2020	TLR5	Toll-like Receptor 5 Activation by the CagY Repeat Domains of Helicobacter pylori	Cell reports	10.1016/j.celrep.2020.108159
2020	Nurr1	Orphan nuclear receptor Nurr1 promotes Helicobacter pylori-associated gastric carcinogenesis by directly enhancing CDK4 expression	EBioMedicine	10.1016/j.ebiom.2020.102672
2021	CLEC4E	Helicobacter pylori metabolites exacerbate gastritis through C-type lectin receptors	The Journal of experimental medicine	10.1084/jem.20200815
2020	LATS2	The Hippo Kinase LATS2 Controls Helicobacter pylori-Induced Epithelial-Mesenchymal Transition and Intestinal Metaplasia in Gastric Mucosa	Cellular and molecular gastroenterology and hepatology	10.1016/j.jcmgh.2019.10.007
2020	ARRDC3	Arrestin domain containing 3 promotes Helicobacter pylori-associated gastritis by regulating protease-activated receptor 1	JCI insight	10.1172/jci.insight.135849
2020	ADM	Helicobacter pylori-induced adrenomedullin modulates IFN-γ-producing T-cell responses and contributes to	Cell death & disease	10.1038/s41419-020-2391-6

		gastritis		
2019	RACK1	Downregulation of tumor suppressor RACK1 by Helicobacter pylori infection promotes gastric carcinogenesis through the integrin β-1/NF-κB signaling pathway	Cancer letters	10.1016/j.canlet.2019.02.039
2020	PUMA	PUMA-mediated epithelial cell apoptosis promotes Helicobacter pylori infection-mediated gastritis	Cell death & disease	10.1038/s41419-020-2339-x
2020	NLRC5	Innate Immune Molecule NLRC5 Protects Mice From Helicobacter-induced Formation of Gastric Lymphoid Tissue	Gastroenterology	10.1053/j.gastro.2020.03.009
2021	CXCR2	Inflammation-Associated Senescence Promotes Helicobacter pylori-Induced Atrophic Gastritis	Cellular and molecular gastroenterology and hepatology	10.1016/j.jcmgh.2020.10.015
2020	CEACAM1<br>CEACAM3<br>CEACAM6	Helicobacter pyloriThe HopQ-CEACAM Interaction Controls CagA Translocation, Phosphorylation, and Phagocytosis of in Neutrophils	mBio	10.1128/mBio.03256-19

Table.2 The 1338 genes were identified between the HP-related genes and meta-dataset (GSE15459 and GSE66229).

gene	HP_genesets	cor	pvalue	Regulation
GKN1	AADAC	0.531426807	1.75E-36	postive
TLR5	ABCA6	0.501543057	4.55E-32	postive
TLR5	ABCA8	0.502290802	3.57E-32	postive
PDIA3	ABCC9	-0.550758737	1.39E-39	negative
CEACAM1	ABHD17C	0.503945387	2.08E-32	postive
CEACAM6	ABHD17C	0.539690747	8.76E-38	postive
PDIA3	ABHD17C	0.519275708	1.23E-34	postive
CD274	ACP5	0.516241486	3.48E-34	postive
ITGA5	ACTA2	0.577701578	2.97E-44	postive
ITGA5	ACTN1	0.655121461	2.01E-60	postive
ITGA5	ACVRL1	0.527814276	6.31E-36	postive
ADM	ADAM12	0.517573236	2.21E-34	postive
HIF1A	ADAM12	0.515636411	4.27E-34	postive
PDIA3	ADAM33	-0.537306577	2.10E-37	negative
ARRDC3	ADAMTS1	0.502906942	2.92E-32	postive
ITGA5	ADAMTS12	0.522221556	4.47E-35	postive
ITGA5	ADAMTS2	0.535773695	3.66E-37	postive
ITGA5	ADAMTS3	0.605226285	1.70E-49	postive
ITGA5	ADAMTS5	0.54201612	3.72E-38	postive
ITGA5	ADAMTS9	0.517944495	1.95E-34	postive
ETS1	ADAP2	0.546048274	8.27E-39	postive
ITGA5	ADCY4	0.539164525	1.06E-37	postive
TLR5	ADH1B	0.505167181	1.40E-32	postive
GKN1	ADH1C	0.536894173	2.44E-37	postive
GKN1	ADH7	0.500352004	6.68E-32	postive
ADM	ADM	1	0	postive
HIF1A	ADM	0.516095765	3.65E-34	postive
IL1B	ADM	0.600193643	1.68E-48	postive
ARRDC3	ADRM1	-0.533965426	7.04E-37	negative
GKN1	ADTRP	0.543614533	2.05E-38	postive
ITGA5	AEBP1	0.567142341	2.26E-42	postive
LATS2	AEBP1	0.540716975	6.01E-38	postive
ITGA5	AFAP1L1	0.516505214	3.18E-34	postive
CEACAM6	AGR3	0.554556932	3.24E-40	postive
CLEC4E	AIF1	0.509874517	2.96E-33	postive

ETS1	AIF1	0.600721186	1.33E-48	postive
CD274	AIM2	0.642615572	1.71E-57	postive
NLRC5	AIM2	0.645247353	4.24E-58	postive
NLRC5	AKNA	0.540046822	7.69E-38	postive
TRAF1	AKNA	0.559163344	5.40E-41	postive
GKN1	AKR1B10	0.657701214	4.79E-61	postive
GKN1	AKR1C1	0.55890819	5.97E-41	postive
ITGA5	AKT3	0.601571525	9.02E-49	postive
PDIA3	AKT3	-0.516623961	3.05E-34	negative
GKN1	ALDH3A1	0.624779699	1.53E-53	postive
CD274	ALOX5AP	0.539398746	9.76E-38	postive
CLEC4E	ALOX5AP	0.64823059	8.60E-59	postive
CXCR2	ALOX5AP	0.595780284	1.22E-47	postive
ETS1	ALOX5AP	0.529743366	3.19E-36	postive
CXCR2	AMICA1	0.508012811	5.48E-33	postive
ETS1	AMICA1	0.525135391	1.62E-35	postive
ITGA5	AMOTL1	0.572752989	2.31E-43	postive
PDIA3	AMOTL1	-0.501478864	4.64E-32	negative
LATS2	AMOTL2	0.57542141	7.66E-44	postive
ADM	ANGPT2	0.53984229	8.29E-38	postive
HIF1A	ANGPT2	0.514484014	6.30E-34	postive
ITGA5	ANGPTL2	0.763268666	4.13E-93	postive
LATS2	ANGPTL2	0.570267802	6.37E-43	postive
ADM	ANGPTL4	0.564101874	7.65E-42	postive
TLR5	ANK2	0.500959798	5.49E-32	postive
ETS1	ANKRD44	0.610387905	1.55E-50	postive
NLRC5	ANKRD44	0.535816372	3.60E-37	postive
TRAF1	ANKRD44	0.550773815	1.38E-39	postive
ITGA5	ANTXR1	0.585386621	1.14E-45	postive
LATS2	ANTXR1	0.527341247	7.46E-36	postive
GKN1	ANXA10	0.611939566	7.45E-51	postive
CEACAM6	ANXA4	0.510376565	2.50E-33	postive
ITGA5	ANXA6	0.565552804	4.28E-42	postive
ETS1	AOAH	0.543887805	1.86E-38	postive
NLRC5	AOAH	0.524391021	2.10E-35	postive
CEACAM6	AOC1	0.534351248	6.12E-37	postive
ITGA5	AOC3	0.538347619	1.43E-37	postive
ARRDC3	AP1S2	0.546143451	7.98E-39	postive
ITGA5	AP1S2	0.500225817	6.96E-32	postive
ITGA5	APLNR	0.503816512	2.17E-32	postive
GKN1	APOBEC1	0.509015746	3.93E-33	postive
CD274	APOBEC3G	0.579594383	1.34E-44	postive
ETS1	APOBEC3G	0.549815286	1.99E-39	postive

NLRC5	APOBEC3G	0.605217529	1.70E-49	postive
CD274	APOL3	0.503774772	2.20E-32	postive
NLRC5	APOL3	0.712200707	8.76E-76	postive
CD274	APOL6	0.550526842	1.52E-39	postive
NLRC5	APOL6	0.678311372	3.04E-66	postive
ADM	AQP9	0.612176478	6.67E-51	postive
CLEC4E	AQP9	0.721043768	1.66E-78	postive
CXCR2	AQP9	0.741919522	2.28E-85	postive
IL1B	AQP9	0.692026172	6.04E-70	postive
TLR5	ARHGAP24	0.546659215	6.58E-39	postive
CD274	ARHGAP25	0.507747417	5.98E-33	postive
CLEC4E	ARHGAP25	0.527272934	7.64E-36	postive
ETS1	ARHGAP25	0.623720685	2.57E-53	postive
NLRC5	ARHGAP25	0.574013962	1.37E-43	postive
TRAF1	ARHGAP25	0.622224268	5.35E-53	postive
CD274	ARHGAP30	0.512141809	1.39E-33	postive
ETS1	ARHGAP30	0.607980075	4.75E-50	postive
NLRC5	ARHGAP30	0.570642109	5.47E-43	postive
TRAF1	ARHGAP30	0.621529126	7.52E-53	postive
ETS1	ARHGAP31	0.585988333	8.84E-46	postive
ITGA5	ARHGAP31	0.610009483	1.85E-50	postive
LATS2	ARHGAP31	0.508610009	4.50E-33	postive
TRAF1	ARHGAP31	0.510560474	2.35E-33	postive
CD274	ARHGAP9	0.56917647	9.93E-43	postive
CLEC4E	ARHGAP9	0.571842229	3.35E-43	postive
CXCR2	ARHGAP9	0.509540265	3.30E-33	postive
ETS1	ARHGAP9	0.667210311	2.16E-63	postive
NLRC5	ARHGAP9	0.631767836	4.64E-55	postive
TRAF1	ARHGAP9	0.64121716	3.57E-57	postive
ETS1	ARHGDIB	0.658320785	3.39E-61	postive
TRAF1	ARHGDIB	0.561749663	1.95E-41	postive
ITGA5	ARHGEF17	0.596779594	7.81E-48	postive
LATS2	ARHGEF17	0.528637295	4.72E-36	postive
ETS1	ARHGEF6	0.567941144	1.64E-42	postive
ETS1	ARL4C	0.52706324	8.23E-36	postive
ARRDC3	ARRDC3	1	0	postive
ARRDC3	ASPN	0.533487323	8.36E-37	postive
ITGB1	ASPN	0.580584721	8.84E-45	postive
NR4A2	ATF3	0.577368131	3.41E-44	postive
CEACAM6	ATP10B	0.603055242	4.59E-49	postive
ARRDC3	ATP10D	0.501384934	4.79E-32	postive
GKN1	ATP2A3	0.538855671	1.19E-37	postive
ITGA5	ATP2B4	0.527349452	7.44E-36	postive

CEACAM6	ATP2C2	0.509198423	3.70E-33	postive
GKN1	ATP4B	0.579382063	1.47E-44	postive
ITGA5	ATP8B2	0.515959575	3.83E-34	postive
ARRDC3	ATP8B4	0.537839856	1.73E-37	postive
ETS1	ATP8B4	0.513435518	8.98E-34	postive
TLR5	AURKA	-0.519271089	1.24E-34	negative
TLR5	AURKB	-0.521693181	5.36E-35	negative
ITGA5	AVPR1A	0.502481106	3.36E-32	postive
ETS1	AXL	0.51065129	2.28E-33	postive
ITGA5	AXL	0.532068655	1.39E-36	postive
GKN1	B3GNT6	0.566819994	2.57E-42	postive
ITGB1	BAG2	0.53715385	2.22E-37	postive
PDIA3	BAI3	-0.53702457	2.32E-37	negative
ETS1	BASP1	0.522164326	4.56E-35	postive
CD274	BATF2	0.528345597	5.23E-36	postive
NLRC5	BATF2	0.519661442	1.08E-34	postive
ETS1	BATF3	0.520820207	7.25E-35	postive
GKN1	BCAS1	0.587688063	4.25E-46	postive
ADM	BCL2A1	0.610616965	1.39E-50	postive
CD274	BCL2A1	0.638540953	1.44E-56	postive
CLEC4E	BCL2A1	0.765678688	4.90E-94	postive
CXCR2	BCL2A1	0.714509301	1.75E-76	postive
ETS1	BCL2A1	0.524383379	2.11E-35	postive
HIF1A	BCL2A1	0.5141272	7.11E-34	postive
IL1B	BCL2A1	0.673816624	4.49E-65	postive
ITGA5	BCL6	0.561210839	2.41E-41	postive
LATS2	BCL6	0.532649566	1.13E-36	postive
ITGA5	BEND6	0.533629492	7.94E-37	postive
ITGA5	BGN	0.588663192	2.78E-46	postive
LATS2	BGN	0.524603147	1.95E-35	postive
ITGA5	BICC1	0.561858546	1.87E-41	postive
CD274	BIN2	0.528309012	5.30E-36	postive
CLEC4E	BIN2	0.530391223	2.53E-36	postive
ETS1	BIN2	0.627740784	3.51E-54	postive
NLRC5	BIN2	0.541428812	4.62E-38	postive
TRAF1	BIN2	0.566191785	3.32E-42	postive
CD274	BIRC3	0.51079175	2.18E-33	postive
IL17RB	BIRC5	0.50115819	5.15E-32	postive
ITGA5	BNC2	0.608072333	4.55E-50	postive
ARRDC3	BNIP2	0.537500528	1.95E-37	postive
ITGA5	BNIP2	0.509916619	2.91E-33	postive
GKN1	BPIFB1	0.638518732	1.46E-56	postive
NR4A2	BRE-AS1	0.58229649	4.28E-45	postive

ITGA5	BRI3BP	-0.531593438	1.65E-36	negative
ITGA5	BSPRY	-0.516476401	3.21E-34	negative
NLRC5	BST2	0.531356235	1.79E-36	postive
ETS1	BTK	0.5691948	9.86E-43	postive
NLRC5	BTN3A1	0.709733265	4.82E-75	postive
NLRC5	BTN3A2	0.541620759	4.30E-38	postive
NLRC5	BTN3A3	0.64891563	5.94E-59	postive
IL17RB	BUB1B	0.50345287	2.45E-32	postive
PRTG	BVES	0.506057424	1.04E-32	postive
ETS1	C10orf128	0.559714516	4.35E-41	postive
TRAP1	C10orf2	0.576804101	4.31E-44	postive
ITGA5	C11orf96	0.54417438	1.67E-38	postive
LATS2	C11orf96	0.511000359	2.03E-33	postive
ETS1	C16orf54	0.546267154	7.62E-39	postive
GKN1	C16orf89	0.520831563	7.22E-35	postive
PDIA3	C19orf10	0.558689453	6.50E-41	postive
CEACAM6	C19orf33	0.518792402	1.46E-34	postive
CEACAM6	C1orf106	0.514418409	6.45E-34	postive
ETS1	C1orf162	0.585943561	9.01E-46	postive
ITGA5	C1orf216	0.531164	1.92E-36	postive
ETS1	C1orf54	0.633227415	2.21E-55	postive
ITGA5	C1orf54	0.582428432	4.05E-45	postive
ETS1	C1QA	0.522181486	4.53E-35	postive
TRAP1	C1QBP	0.51679578	2.88E-34	postive
ETS1	C1QC	0.501047365	5.34E-32	postive
MCOLN1	C1QC	0.500160248	7.11E-32	postive
ETS1	C1R	0.505623214	1.20E-32	postive
ITGA5	C1R	0.591396544	8.45E-47	postive
LATS2	C1R	0.590491942	1.26E-46	postive
ARRDC3	C1S	0.537265949	2.13E-37	postive
ETS1	C1S	0.54596809	8.52E-39	postive
LATS2	C1S	0.517668155	2.14E-34	postive
LATS2	C3	0.531669523	1.60E-36	postive
CLEC4E	C3AR1	0.506955925	7.77E-33	postive
ETS1	C3AR1	0.550311216	1.65E-39	postive
CEACAM6	C4BPB	0.502603111	3.23E-32	postive
ARRDC3	C4orf3	0.57067992	5.39E-43	postive
ADM	C5AR1	0.50578459	1.14E-32	postive
CLEC4E	C5AR1	0.635918401	5.58E-56	postive
CXCR2	C5AR1	0.627498037	3.96E-54	postive
ETS1	C5AR1	0.507940396	5.61E-33	postive
ITGA5	C5AR1	0.526365101	1.05E-35	postive
MCOLN1	C5AR1	0.534908359	5.01E-37	postive

NLRC5	C5orf56	0.636263123	4.68E-56	postive
GKN1	C6orf58	0.691172747	1.04E-69	postive
ITGA5	C8orf88	0.533052634	9.77E-37	postive
PDIA3	C8orf88	-0.511109795	1.96E-33	negative
GKN1	CA2	0.604311284	2.58E-49	postive
GKN1	CA9	0.504352187	1.82E-32	postive
ETS1	CALCRL	0.587920774	3.84E-46	postive
ITGA5	CALD1	0.652915225	6.76E-60	postive
ITGB1	CALD1	0.517843013	2.01E-34	postive
LATS2	CALD1	0.52627964	1.08E-35	postive
ITGA5	CALHM2	0.527187303	7.88E-36	postive
CEACAM6	CALML4	0.526698419	9.36E-36	postive
ITGA5	CALU	0.521639019	5.46E-35	postive
ITGB1	CALU	0.593673954	3.10E-47	postive
PDIA3	CAND2	-0.504086992	1.99E-32	negative
PRTG	CAND2	0.522476286	4.09E-35	postive
GKN1	CAPN13	0.56131452	2.31E-41	postive
GKN1	CAPN8	0.537864417	1.71E-37	postive
GKN1	CAPN9	0.667323801	2.02E-63	postive
NLRC5	CARD16	0.602561641	5.75E-49	postive
NLRC5	CASP1	0.568337904	1.40E-42	postive
CLEC4E	CASS4	0.516296163	3.41E-34	postive
ITGA5	CAV1	0.65623895	1.08E-60	postive
LATS2	CAV1	0.509697852	3.13E-33	postive
ITGB1	CAV2	0.500696994	5.98E-32	postive
PRTG	CC2D2A	0.504607215	1.68E-32	postive
ETS1	CCDC102B	0.530909171	2.11E-36	postive
IL17RB	CCDC69	-0.517908256	1.97E-34	negative
ITGA5	CCDC69	0.531465172	1.73E-36	postive
ARRDC3	CCDC80	0.507383211	6.75E-33	postive
ITGA5	CCDC80	0.523935213	2.46E-35	postive
TRAP1	CCDC86	0.544521429	1.46E-38	postive
CD274	CCL18	0.539174193	1.06E-37	postive
CLEC4E	CCL18	0.601599778	8.91E-49	postive
CXCR2	CCL18	0.503704147	2.25E-32	postive
ETS1	CCL2	0.519945131	9.80E-35	postive
ITGA5	CCL2	0.551402883	1.09E-39	postive
TRAF1	CCL22	0.602598175	5.66E-49	postive
ADM	CCL4	0.50847247	4.71E-33	postive
CD274	CCL4	0.663044533	2.36E-62	postive
CLEC4E	CCL4	0.703201986	4.05E-73	postive
CXCR2	CCL4	0.570700045	5.34E-43	postive
IL1B	CCL4	0.564823292	5.74E-42	postive

CD274	CCL5	0.565283358	4.77E-42	postive
ETS1	CCL5	0.543931783	1.83E-38	postive
NLRC5	CCL5	0.647152351	1.53E-58	postive
TRAF1	CCL5	0.508666428	4.41E-33	postive
ADM	CCL7	0.520002746	9.61E-35	postive
CD274	CCL8	0.51368146	8.26E-34	postive
TLR5	CCNB1	-0.503502829	2.41E-32	negative
TRAP1	CCNF	0.553282481	5.30E-40	postive
CD274	CCR1	0.601760033	8.28E-49	postive
CLEC4E	CCR1	0.693245947	2.77E-70	postive
CXCR2	CCR1	0.640986395	4.03E-57	postive
ETS1	CCR1	0.581313258	6.50E-45	postive
TRAF1	CCR1	0.502850622	2.98E-32	postive
ETS1	CCR2	0.518887533	1.41E-34	postive
CD274	CCR5	0.514685741	5.89E-34	postive
ETS1	CCR5	0.591054732	9.81E-47	postive
NLRC5	CCR5	0.597561411	5.51E-48	postive
TRAF1	CCR5	0.53552552	4.01E-37	postive
ETS1	CCR7	0.517113468	2.58E-34	postive
TRAF1	CCR7	0.631436123	5.49E-55	postive
ITGA5	CD109	0.571539468	3.79E-43	postive
CLEC4E	CD14	0.537434312	2.00E-37	postive
ETS1	CD14	0.531416376	1.76E-36	postive
ITGA5	CD14	0.503374225	2.51E-32	postive
MCOLN1	CD14	0.527268307	7.66E-36	postive
CLEC4E	CD163	0.506715081	8.41E-33	postive
ETS1	CD163	0.576353811	5.20E-44	postive
ETS1	CD1D	0.504913733	1.52E-32	postive
ETS1	CD2	0.538112478	1.56E-37	postive
NLRC5	CD2	0.633933614	1.54E-55	postive
TRAF1	CD2	0.543386249	2.24E-38	postive
ETS1	CD200	0.620072638	1.53E-52	postive
ITGA5	CD200	0.503692768	2.26E-32	postive
NLRC5	CD244	0.506082584	1.04E-32	postive
CD274	CD247	0.520512304	8.06E-35	postive
ETS1	CD247	0.535139928	4.61E-37	postive
NLRC5	CD247	0.626391892	6.87E-54	postive
TRAF1	CD247	0.5560406	1.83E-40	postive
ITGA5	CD248	0.65805168	3.94E-61	postive
LATS2	CD248	0.541907371	3.87E-38	postive
CD274	CD274	1	0	postive
CLEC4E	CD274	0.630767539	7.69E-55	postive
NLRC5	CD274	0.597565266	5.50E-48	postive

ETS1	CD28	0.529609836	3.34E-36	postive
TRAF1	CD28	0.519397474	1.18E-34	postive
CD274	CD300A	0.614286768	2.46E-51	postive
CLEC4E	CD300A	0.66826056	1.17E-63	postive
CXCR2	CD300A	0.633826263	1.63E-55	postive
ETS1	CD300A	0.561609069	2.06E-41	postive
HPSE	CD300A	0.529925854	2.99E-36	postive
MCOLN1	CD300A	0.50876193	4.28E-33	postive
CD274	CD300LF	0.577718627	2.95E-44	postive
CLEC4E	CD300LF	0.561634507	2.04E-41	postive
ETS1	CD300LF	0.540888679	5.64E-38	postive
NLRC5	CD300LF	0.514111548	7.15E-34	postive
ITGA5	CD34	0.5822913	4.29E-45	postive
CD274	CD38	0.593724787	3.03E-47	postive
ETS1	CD38	0.51085362	2.13E-33	postive
NLRC5	CD38	0.554979245	2.75E-40	postive
CD274	CD3D	0.50362937	2.31E-32	postive
ETS1	CD3D	0.511699413	1.61E-33	postive
NLRC5	CD3D	0.628457037	2.45E-54	postive
TRAF1	CD3D	0.55035142	1.63E-39	postive
CD274	CD3G	0.519839352	1.02E-34	postive
ETS1	CD3G	0.543750586	1.95E-38	postive
NLRC5	CD3G	0.580513959	9.11E-45	postive
TRAF1	CD40	0.512271686	1.33E-33	postive
CD44	CD44	1	0	postive
CD274	CD48	0.50696331	7.75E-33	postive
ETS1	CD48	0.590568096	1.21E-46	postive
NLRC5	CD48	0.555108233	2.62E-40	postive
TRAF1	CD48	0.59134569	8.64E-47	postive
ETS1	CD52	0.539551715	9.22E-38	postive
NLRC5	CD52	0.553042323	5.81E-40	postive
TRAF1	CD52	0.520809183	7.28E-35	postive
CD274	CD53	0.601002685	1.17E-48	postive
CLEC4E	CD53	0.623066878	3.54E-53	postive
CXCR2	CD53	0.503783578	2.20E-32	postive
ETS1	CD53	0.652567122	8.17E-60	postive
NLRC5	CD53	0.580905504	7.72E-45	postive
TRAF1	CD53	0.603055412	4.59E-49	postive
ARRDC3	CD69	0.526237976	1.10E-35	postive
CD274	CD69	0.533588647	8.06E-37	postive
CLEC4E	CD69	0.592043384	6.36E-47	postive
CXCR2	CD69	0.506183535	1.00E-32	postive
ETS1	CD69	0.641958584	2.42E-57	postive

TRAF1	CD69	0.561406112	2.23E-41	postive
ETS1	CD72	0.565277977	4.78E-42	postive
NLRC5	CD74	0.625322099	1.17E-53	postive
CLEC4E	CD83	0.500520733	6.33E-32	postive
ETS1	CD83	0.548885786	2.84E-39	postive
TRAF1	CD83	0.545573744	9.88E-39	postive
CD274	CD84	0.553442985	4.98E-40	postive
CLEC4E	CD84	0.517859718	2.00E-34	postive
ETS1	CD84	0.598612968	3.44E-48	postive
NLRC5	CD84	0.52670283	9.34E-36	postive
CD274	CD86	0.620928608	1.01E-52	postive
CLEC4E	CD86	0.597260225	6.30E-48	postive
ETS1	CD86	0.626436393	6.72E-54	postive
NLRC5	CD8A	0.557987763	8.55E-41	postive
ETS1	CD93	0.598012367	4.50E-48	postive
HIF1A	CD93	0.51827552	1.74E-34	postive
ITGA5	CD93	0.668112068	1.28E-63	postive
LATS2	CD93	0.554659234	3.12E-40	postive
CD274	CD96	0.563403788	1.01E-41	postive
NLRC5	CD96	0.659457932	1.79E-61	postive
ITGA5	CDC42EP3	0.529871371	3.05E-36	postive
IL17RB	CDC45	0.52376412	2.61E-35	postive
TLR5	CDCA3	-0.516474264	3.21E-34	negative
TLR5	CDCA5	-0.510383229	2.50E-33	negative
IL17RB	CDCA7	0.517875349	1.99E-34	postive
TLR5	CDCA8	-0.507310105	6.91E-33	negative
ITGA5	CDH11	0.584433886	1.72E-45	postive
CEACAM6	CDH17	0.706913873	3.31E-74	postive
ETS1	CDH5	0.585802588	9.58E-46	postive
ITGA5	CDH5	0.637644627	2.29E-56	postive
ITGA5	CDS1	-0.544835209	1.30E-38	negative
TRAP1	CDT1	0.5680396	1.57E-42	postive
CEACAM6	CDX1	0.522335327	4.29E-35	postive
CEACAM1	CEACAM1	1	0	postive
CEACAM6	CEACAM1	0.5364045	2.91E-37	postive
CEACAM1	CEACAM5	0.56518566	4.96E-42	postive
CEACAM6	CEACAM5	0.837468253	4.74E-128	postive
CEACAM1	CEACAM6	0.536404508	2.91E-37	postive
CEACAM6	CEACAM6	1	0	postive
ADM	CEBPB	0.536601029	2.71E-37	postive
ETS1	CECR1	0.537778336	1.77E-37	postive
ARRDC3	CELF2	0.511278294	1.85E-33	postive
IL17RB	CENPM	0.540797839	5.83E-38	postive

ARRDC3	CFL2	0.502328018	3.53E-32	postive
ITGA5	CFL2	0.529771644	3.16E-36	postive
ITGB1	CFL2	0.530960404	2.07E-36	postive
CLEC4E	CFP	0.512573912	1.20E-33	postive
IL17RB	CHDH	0.751879541	6.99E-89	postive
ITGA5	CHN1	0.603157809	4.38E-49	postive
ITGA5	CHRDL2	0.511668225	1.62E-33	postive
CLEC4E	CHST11	0.508010557	5.48E-33	postive
ETS1	CHST11	0.616222417	9.76E-52	postive
ITGA5	CHST15	0.541083273	5.25E-38	postive
HIF1A	CHSY1	0.51915023	1.29E-34	postive
ITGA5	CHSY1	0.542731074	2.85E-38	postive
LATS2	CHSY1	0.548512845	3.27E-39	postive
ITGA5	CHSY3	0.651122089	1.80E-59	postive
LATS2	CHSY3	0.514552741	6.16E-34	postive
NLRC5	CIITA	0.536274084	3.05E-37	postive
PDIA3	CIRBP	-0.507849212	5.78E-33	negative
CEACAM1	CLDN4	0.598777306	3.19E-48	postive
CEACAM6	CLDN4	0.513727355	8.14E-34	postive
CEACAM1	CLDN7	0.554519003	3.29E-40	postive
CEACAM6	CLDN7	0.516078225	3.67E-34	postive
ITGA5	CLEC11A	0.609162514	2.74E-50	postive
CD274	CLEC12A	0.508678743	4.40E-33	postive
CLEC4E	CLEC12A	0.608123199	4.45E-50	postive
ETS1	CLEC14A	0.540731813	5.98E-38	postive
ITGA5	CLEC14A	0.550825517	1.36E-39	postive
ETS1	CLEC1A	0.511366006	1.80E-33	postive
ITGA5	CLEC1A	0.568324649	1.40E-42	postive
ARRDC3	CLEC2B	0.599520803	2.28E-48	postive
ETS1	CLEC2B	0.652562299	8.20E-60	postive
CD274	CLEC2D	0.546037875	8.30E-39	postive
ETS1	CLEC2D	0.595206564	1.57E-47	postive
NLRC5	CLEC2D	0.615467882	1.40E-51	postive
TRAF1	CLEC2D	0.541582903	4.36E-38	postive
CD274	CLEC4A	0.603191705	4.31E-49	postive
CLEC4E	CLEC4A	0.716053985	5.88E-77	postive
CXCR2	CLEC4A	0.636786736	3.57E-56	postive
ETS1	CLEC4A	0.59038176	1.32E-46	postive
CD274	CLEC4D	0.545898757	8.75E-39	postive
CEACAM3	CLEC4D	0.500965296	5.48E-32	postive
CLEC4E	CLEC4D	0.75563781	2.98E-90	postive
CXCR2	CLEC4D	0.55174007	9.57E-40	postive
IL1B	CLEC4D	0.512503925	1.23E-33	postive

CD274	CLEC4E	0.630767533	7.69E-55	postive
CLEC4E	CLEC4E	1	0	postive
CXCR2	CLEC4E	0.707658829	2.00E-74	postive
IL1B	CLEC4E	0.51555407	4.39E-34	postive
TRAF1	CLEC4E	0.517998266	1.91E-34	postive
ADM	CLEC5A	0.539842219	8.29E-38	postive
IL1B	CLEC5A	0.506225554	9.88E-33	postive
CD274	CLEC7A	0.653495201	4.92E-60	postive
CLEC4E	CLEC7A	0.710844902	2.24E-75	postive
CXCR2	CLEC7A	0.56789063	1.67E-42	postive
ETS1	CLEC7A	0.635081333	8.58E-56	postive
ARRDC3	CLIC2	0.51110038	1.96E-33	postive
ETS1	CLIC2	0.539691473	8.76E-38	postive
ARRDC3	CLIC4	0.538420311	1.40E-37	postive
ITGA5	CLIC4	0.61165645	8.52E-51	postive
ITGB1	CLIC4	0.587161189	5.33E-46	postive
PDIA3	CLIP3	-0.510772763	2.19E-33	negative
ITGA5	CLMP	0.634177652	1.36E-55	postive
CEACAM6	CLRN3	0.54850654	3.28E-39	postive
ARRDC3	CMAHP	0.52439425	2.10E-35	postive
ETS1	CMAHP	0.504208222	1.91E-32	postive
ETS1	CMKLR1	0.571149791	4.45E-43	postive
CEACAM3	CMTM2	0.512773447	1.12E-33	postive
CLEC4E	CMTM2	0.67795882	3.76E-66	postive
CXCR2	CMTM2	0.787786897	4.42E-103	postive
IL1B	CMTM2	0.59262172	4.93E-47	postive
ETS1	CMTM3	0.519921826	9.88E-35	postive
ITGA5	CMTM3	0.516045516	3.72E-34	postive
PRTG	CNKSR2	0.511416248	1.77E-33	postive
ITGA5	CNN1	0.526065607	1.17E-35	postive
ITGB1	CNN3	0.537934322	1.67E-37	postive
ITGA5	CNRIP1	0.604153625	2.78E-49	postive
ARRDC3	COASY	-0.561653177	2.02E-41	negative
ITGA5	COL12A1	0.659166706	2.11E-61	postive
LATS2	COL12A1	0.525544628	1.40E-35	postive
ETS1	COL15A1	0.505421135	1.29E-32	postive
ITGA5	COL15A1	0.742201642	1.82E-85	postive
LATS2	COL15A1	0.540281424	7.05E-38	postive
ITGA5	COL16A1	0.503822195	2.17E-32	postive
ITGA5	COL18A1	0.607812475	5.14E-50	postive
ITGA5	COL1A1	0.581960627	4.94E-45	postive
ITGA5	COL1A2	0.625598609	1.02E-53	postive
LATS2	COL1A2	0.516093377	3.66E-34	postive

ITGA5	COL3A1	0.62622448	7.46E-54	postive
LATS2	COL3A1	0.500197333	7.02E-32	postive
ITGA5	COL4A1	0.688990691	4.15E-69	postive
LATS2	COL4A1	0.510774101	2.19E-33	postive
ITGA5	COL4A2	0.753660787	1.58E-89	postive
LATS2	COL4A2	0.524208014	2.24E-35	postive
ITGA5	COL5A1	0.649279377	4.88E-59	postive
LATS2	COL5A1	0.502614852	3.21E-32	postive
ITGA5	COL5A2	0.624698248	1.59E-53	postive
ITGA5	COL5A3	0.639477	8.85E-57	postive
ITGA5	COL6A1	0.678424233	2.84E-66	postive
LATS2	COL6A1	0.532160604	1.35E-36	postive
ITGA5	COL6A2	0.783120217	4.39E-101	postive
LATS2	COL6A2	0.569716548	7.98E-43	postive
ITGA5	COL6A3	0.671790439	1.49E-64	postive
LATS2	COL6A3	0.538906626	1.17E-37	postive
HIF1A	COL7A1	0.569835349	7.60E-43	postive
ITGA5	COL7A1	0.550922859	1.31E-39	postive
ITGA5	COL8A1	0.530800485	2.19E-36	postive
ARRDC3	COLEC12	0.512475921	1.24E-33	postive
ITGA5	COLEC12	0.513169707	9.82E-34	postive
ITGA5	COPZ2	0.553708787	4.50E-40	postive
CD274	CORO1A	0.511220426	1.89E-33	postive
ETS1	CORO1A	0.607779507	5.22E-50	postive
NLRC5	CORO1A	0.567335163	2.09E-42	postive
TRAF1	CORO1A	0.58564938	1.02E-45	postive
ITGA5	CORO1C	0.52201622	4.80E-35	postive
ITGB1	CORO1C	0.526278909	1.08E-35	postive
ITGA5	COX7A1	0.620570804	1.20E-52	postive
LATS2	COX7A1	0.51585015	3.97E-34	postive
ITGA5	CPXM1	0.539445009	9.59E-38	postive
ITGA5	CPXM2	0.536479494	2.83E-37	postive
PDIA3	CPXM2	-0.51104085	2.00E-33	negative
CLEC4E	CR1	0.536943135	2.39E-37	postive
ETS1	CR1	0.549795298	2.01E-39	postive
TRAF1	CR1	0.51220148	1.36E-33	postive
ARRDC3	CREM	0.512677704	1.16E-33	postive
ITGA5	CRISPLD2	0.581006104	7.40E-45	postive
LATS2	CRISPLD2	0.508757894	4.28E-33	postive
CD274	CRTAM	0.517255776	2.46E-34	postive
ETS1	CRTAM	0.519285202	1.23E-34	postive
NLRC5	CRTAM	0.573839326	1.47E-43	postive
ITGA5	CRYAB	0.545969997	8.52E-39	postive

ETS1	CSF1R	0.501562101	4.52E-32	postive
CD274	CSF2RA	0.500759623	5.86E-32	postive
CLEC4E	CSF2RA	0.542657733	2.93E-38	postive
ETS1	CSF2RA	0.549097062	2.62E-39	postive
CD274	CSF2RB	0.541665206	4.23E-38	postive
CLEC4E	CSF2RB	0.652445833	8.74E-60	postive
CXCR2	CSF2RB	0.625996881	8.35E-54	postive
ETS1	CSF2RB	0.677773086	4.20E-66	postive
NLRC5	CSF2RB	0.500745728	5.89E-32	postive
TRAF1	CSF2RB	0.579817591	1.22E-44	postive
HIF1A	CSF3	0.50312548	2.72E-32	postive
CEACAM3	CSF3R	0.50092508	5.55E-32	postive
CLEC4E	CSF3R	0.737638923	6.59E-84	postive
CXCR2	CSF3R	0.79937339	2.89E-108	postive
ITGA5	CSGALNACT1	0.546291902	7.55E-39	postive
LATS2	CSGALNACT1	0.515791016	4.05E-34	postive
ARRDC3	CSGALNACT2	0.580571202	8.89E-45	postive
ETS1	CSGALNACT2	0.521790156	5.19E-35	postive
ITGA5	CSGALNACT2	0.501729619	4.28E-32	postive
ITGA5	CSPG4	0.651084763	1.84E-59	postive
NR4A2	CSRNP1	0.607059062	7.29E-50	postive
ITGA5	CSRP1	0.527943216	6.03E-36	postive
ETS1	CST7	0.538776788	1.23E-37	postive
TRAF1	CST7	0.549107528	2.61E-39	postive
LATS2	CTGF	0.509496574	3.35E-33	postive
CD274	CTLA4	0.558899996	5.99E-41	postive
CD274	CTSC	0.529078275	4.04E-36	postive
ETS1	CTSC	0.502131648	3.76E-32	postive
GKN1	CTSE	0.504824847	1.56E-32	postive
CLEC4E	CTSL	0.542343354	3.29E-38	postive
ETS1	CTSL	0.516529898	3.15E-34	postive
ITGA5	CTSL	0.510393977	2.49E-33	postive
CLEC4E	CTSLP8	0.569240769	9.68E-43	postive
ARRDC3	CTSO	0.501986256	3.94E-32	postive
CTTN	CTTN	1	0	postive
GKN1	CWH43	0.546650067	6.60E-39	postive
ITGA5	CXADR	-0.519356323	1.20E-34	negative
HIF1A	CXCL1	0.548758508	2.98E-39	postive
IL1B	CXCL1	0.615356977	1.48E-51	postive
MMP10	CXCL1	0.564198876	7.36E-42	postive
CD274	CXCL10	0.622015883	5.93E-53	postive
NLRC5	CXCL10	0.526868276	8.81E-36	postive
CD274	CXCL11	0.606257326	1.06E-49	postive

NLRC5	CXCL11	0.576230951	5.48E-44	postive
ITGA5	CXCL12	0.54676375	6.32E-39	postive
NLRC5	CXCL13	0.5032477	2.62E-32	postive
GKN1	CXCL14	0.508315675	4.96E-33	postive
GKN1	CXCL17	0.601295814	1.02E-48	postive
IL1B	CXCL2	0.528349257	5.23E-36	postive
IL1B	CXCL3	0.567494033	1.96E-42	postive
HIF1A	CXCL6	0.584096362	1.99E-45	postive
IL1B	CXCL6	0.557014519	1.25E-40	postive
MMP10	CXCL6	0.569102177	1.02E-42	postive
ADM	CXCL8	0.590486926	1.26E-46	postive
CLEC4E	CXCL8	0.508544689	4.60E-33	postive
CXCR2	CXCL8	0.546802694	6.23E-39	postive
HIF1A	CXCL8	0.503625312	2.31E-32	postive
IL1B	CXCL8	0.724335892	1.52E-79	postive
MMP10	CXCL8	0.543283526	2.32E-38	postive
CD274	CXCL9	0.630119693	1.07E-54	postive
NLRC5	CXCL9	0.564537919	6.43E-42	postive
CEACAM3	CXCR1	0.560805886	2.83E-41	postive
CLEC4E	CXCR1	0.539628899	8.97E-38	postive
CXCR2	CXCR1	0.681111278	5.54E-67	postive
CLEC4E	CXCR2	0.707658819	2.00E-74	postive
CXCR2	CXCR2	1	0	postive
IL1B	CXCR2	0.554707798	3.06E-40	postive
ETS1	CXCR4	0.532276585	1.29E-36	postive
CD274	CXCR6	0.560430217	3.28E-41	postive
NLRC5	CXCR6	0.598156205	4.22E-48	postive
ITGA5	CXorf36	0.569045587	1.05E-42	postive
CD274	CYBB	0.621358889	8.17E-53	postive
CLEC4E	CYBB	0.57112879	4.49E-43	postive
ETS1	CYBB	0.561844638	1.88E-41	postive
GKN1	CYP2C18	0.528277517	5.36E-36	postive
LATS2	CYR61	0.524292134	2.17E-35	postive
ETS1	CYSLTR1	0.553746331	4.43E-40	postive
GKN1	CYSTM1	0.530264127	2.65E-36	postive
NLRC5	CYTIP	0.512827841	1.10E-33	postive
TRAF1	CYTIP	0.524600649	1.95E-35	postive
ITGA5	CYTL1	0.53587274	3.53E-37	postive
ETS1	CYYR1	0.541254392	4.93E-38	postive
CD274	DAPP1	0.519668058	1.08E-34	postive
NLRC5	DAPP1	0.538099873	1.57E-37	postive
ITGA5	DCHS1	0.637502731	2.47E-56	postive
ARRDC3	DCN	0.521910242	4.97E-35	postive

ITGA5	DCN	0.530797136	2.19E-36	postive
IL17RB	DCTPP1	0.500528211	6.31E-32	postive
TRAP1	DCTPP1	0.613114601	4.28E-51	postive
ITGA5	DDR2	0.633419026	2.01E-55	postive
LATS2	DDR2	0.523877203	2.51E-35	postive
ARRDC3	DDX27	-0.504489878	1.74E-32	negative
NLRC5	DDX58	0.518310495	1.72E-34	postive
NLRC5	DENND1C	0.534751736	5.30E-37	postive
TRAF1	DENND1C	0.591424045	8.35E-47	postive
ITGA5	DENND5A	0.671349736	1.93E-64	postive
LATS2	DENND5A	0.515657177	4.24E-34	postive
ITGA5	DKK3	0.579388141	1.46E-44	postive
ITGA5	DLC1	0.59402604	2.66E-47	postive
ARRDC3	DNAJB4	0.603184139	4.33E-49	postive
ITGB1	DNAJB4	0.572328153	2.74E-43	postive
ITGA5	DNAJB5	0.566261021	3.22E-42	postive
PDIA3	DNAJC18	-0.503483431	2.42E-32	negative
ITGA5	DNM3OS	0.554019598	3.99E-40	postive
ETS1	DOCK10	0.652822084	7.11E-60	postive
NLRC5	DOCK10	0.511157399	1.93E-33	postive
TRAF1	DOCK10	0.528153606	5.60E-36	postive
ETS1	DOCK2	0.611848731	7.78E-51	postive
NLRC5	DOCK2	0.514520748	6.23E-34	postive
TRAF1	DOCK2	0.544547376	1.45E-38	postive
ETS1	DOCK4	0.508081968	5.36E-33	postive
ITGA5	DOCK4	0.553145308	5.59E-40	postive
CD274	DOCK8	0.518969679	1.37E-34	postive
ETS1	DOCK8	0.658904071	2.45E-61	postive
NLRC5	DOCK8	0.558986705	5.79E-41	postive
TRAF1	DOCK8	0.52530483	1.53E-35	postive
NLRC5	DOK2	0.500944854	5.52E-32	postive
TRAF1	DOK2	0.533069662	9.71E-37	postive
CD274	DOK3	0.548940328	2.78E-39	postive
CLEC4E	DOK3	0.627355172	4.25E-54	postive
CXCR2	DOK3	0.52885731	4.37E-36	postive
ETS1	DOK3	0.565748663	3.96E-42	postive
NLRC5	DOK3	0.541917924	3.86E-38	postive
TRAF1	DOK3	0.611222459	1.04E-50	postive
GKN1	DPCR1	0.756069168	2.07E-90	postive
PDIA3	DPY19L2	-0.520468425	8.18E-35	negative
ARRDC3	DPYD	0.515019468	5.26E-34	postive
ITGA5	DPYSL3	0.629057516	1.82E-54	postive
CEACAM6	DSC2	0.503819075	2.17E-32	postive

ETS1	DSE	0.54262817	2.96E-38	postive
ITGA5	DSE	0.508536174	4.61E-33	postive
ITGA5	DSEL	0.510132918	2.71E-33	postive
TRAP1	DTL	0.501563285	4.52E-32	postive
PDIA3	DTNA	-0.516127689	3.61E-34	negative
PRTG	DTNA	0.581949866	4.96E-45	postive
NR4A2	DUSP1	0.579969452	1.15E-44	postive
ETS1	DYSF	0.521835761	5.10E-35	postive
ITGA5	DYSF	0.707827021	1.78E-74	postive
ITGA5	DZIP1	0.521669626	5.41E-35	postive
ITGA5	EBF1	0.530739585	2.24E-36	postive
ITGA5	EBF2	0.508625323	4.47E-33	postive
ARRDC3	ECM2	0.500416852	6.54E-32	postive
ETS1	ECSCR	0.563085124	1.15E-41	postive
ITGA5	ECSCR	0.643392593	1.13E-57	postive
LATS2	ECSCR	0.50485951	1.55E-32	postive
IL17RB	ECT2	0.500168135	7.09E-32	postive
ITGA5	EDNRA	0.634621151	1.09E-55	postive
ITGB1	EDNRA	0.557594258	9.97E-41	postive
ITGA5	EFEMP2	0.677153921	6.11E-66	postive
LATS2	EFEMP2	0.570832003	5.06E-43	postive
ITGA5	EFS	0.605329977	1.62E-49	postive
ADM	EGFL6	0.531873008	1.49E-36	postive
HIF1A	EGFL6	0.541972201	3.78E-38	postive
NR4A2	EGR1	0.514712243	5.84E-34	postive
NR4A2	EGR3	0.57264386	2.41E-43	postive
ITGA5	EHD2	0.673942468	4.17E-65	postive
LATS2	EHD2	0.567476369	1.98E-42	postive
USF1	EIF5A	0.531208759	1.89E-36	postive
ARRDC3	EIF6	-0.534756819	5.29E-37	negative
ETS1	ELMO1	0.528108721	5.69E-36	postive
ETS1	ELTD1	0.603360655	3.99E-49	postive
ITGA5	ELTD1	0.624465951	1.78E-53	postive
LATS2	ELTD1	0.514782272	5.70E-34	postive
ITGA5	EMCN	0.513051098	1.02E-33	postive
ITGA5	EMILIN1	0.665538561	5.66E-63	postive
ITGA5	EML1	0.523338865	3.03E-35	postive
ITGA5	EMP3	0.670969299	2.41E-64	postive
LATS2	EMP3	0.510287393	2.58E-33	postive
CD274	EMR2	0.608980384	2.98E-50	postive
CLEC4E	EMR2	0.685571737	3.54E-68	postive
CXCR2	EMR2	0.686767878	1.68E-68	postive
ETS1	EMR2	0.54445209	1.50E-38	postive

HIF1A	EMR2	0.5094014	3.46E-33	postive
IL1B	EMR2	0.500984076	5.45E-32	postive
CLEC4E	EMR3	0.646768871	1.88E-58	postive
CXCR2	EMR3	0.668546084	9.94E-64	postive
IL1B	EMR3	0.510929012	2.08E-33	postive
ETS1	ENG	0.540338071	6.91E-38	postive
ITGA5	ENG	0.602219765	6.72E-49	postive
ETS1	ENTPD1	0.558670944	6.55E-41	postive
ITGA5	ENTPD1	0.565067986	5.20E-42	postive
ITGA5	EOGT	0.579199801	1.58E-44	postive
NLRC5	EOMES	0.537949196	1.66E-37	postive
ITGA5	EPB41L4B	-0.514672737	5.91E-34	negative
CEACAM1	EPCAM	0.515199228	4.95E-34	postive
USF1	EPM2AIP1	-0.533897321	7.21E-37	negative
CEACAM6	EPS8L3	0.519928346	9.86E-35	postive
CD274	EPSTI1	0.520471179	8.18E-35	postive
NLRC5	EPSTI1	0.59457646	2.08E-47	postive
ETS1	ERG	0.532167149	1.34E-36	postive
ITGA5	ERG	0.52528016	1.54E-35	postive
ITGA5	ESAM	0.54707279	5.63E-39	postive
ETS1	ETS1	1	0	postive
CD274	ETV7	0.507338333	6.85E-33	postive
NLRC5	ETV7	0.563740094	8.84E-42	postive
ARRDC3	EVI2A	0.537537933	1.93E-37	postive
CLEC4E	EVI2A	0.526705099	9.33E-36	postive
ETS1	EVI2A	0.647014015	1.65E-58	postive
CD274	EVI2B	0.513997774	7.43E-34	postive
CLEC4E	EVI2B	0.534125732	6.64E-37	postive
ETS1	EVI2B	0.616145924	1.01E-51	postive
NLRC5	EVI2B	0.545948537	8.59E-39	postive
TRAF1	EVI2B	0.546208158	7.79E-39	postive
IL17RB	EXO1	0.509487482	3.36E-33	postive
TRAP1	EXOSC4	0.542107024	3.60E-38	postive
PDIA3	F11R	0.504988514	1.48E-32	postive
CTTN	FADD	0.650456085	2.58E-59	postive
ITGA5	FAM101B	0.629445201	1.50E-54	postive
TLR5	FAM107A	0.522697401	3.79E-35	postive
ITGA5	FAM124A	0.571489801	3.87E-43	postive
ITGA5	FAM127A	0.505901595	1.10E-32	postive
ITGA5	FAM167B	0.551774514	9.45E-40	postive
TRAP1	FAM195A	0.627076308	4.89E-54	postive
ITGA5	FAM20C	0.528119577	5.67E-36	postive
TLR5	FAM228B	0.545627435	9.69E-39	postive

ITGA5	FAM26E	0.513612999	8.46E-34	postive
CD274	FAM26F	0.610081962	1.78E-50	postive
ETS1	FAM26F	0.516673969	3.00E-34	postive
NLRC5	FAM26F	0.566762759	2.63E-42	postive
GKN1	FAM3B	0.565775689	3.92E-42	postive
ARRDC3	FAM49A	0.525638508	1.36E-35	postive
ETS1	FAM49A	0.655048862	2.09E-60	postive
CLEC4E	FAM65B	0.514734954	5.79E-34	postive
CXCR2	FAM65B	0.516674048	3.00E-34	postive
ETS1	FAM65B	0.584824698	1.46E-45	postive
TRAF1	FAM65B	0.589091008	2.31E-46	postive
ARRDC3	FAM83G	-0.506906222	7.90E-33	negative
ITGA5	FAP	0.502219127	3.65E-32	postive
ITGB1	FAP	0.533657525	7.86E-37	postive
CD274	FASLG	0.57592931	6.21E-44	postive
NLRC5	FASLG	0.588231068	3.36E-46	postive
PDIA3	FAT3	-0.501209703	5.07E-32	negative
TRAP1	FBL	0.514818277	5.63E-34	postive
TLR5	FBLN5	0.511213594	1.89E-33	postive
ITGA5	FBN1	0.589860048	1.65E-46	postive
LATS2	FBN1	0.526384297	1.04E-35	postive
GKN1	FBP2	0.541517233	4.47E-38	postive
ARRDC3	FBXL5	0.554279004	3.61E-40	postive
ITGA5	FBXL7	0.619855929	1.70E-52	postive
PDIA3	FBXL7	-0.510732627	2.22E-33	negative
CEACAM3	FCAR	0.517414784	2.33E-34	postive
CLEC4E	FCAR	0.620467487	1.26E-52	postive
CXCR2	FCAR	0.504787224	1.58E-32	postive
IL1B	FCAR	0.541250899	4.93E-38	postive
CD274	FCER1G	0.583221601	2.89E-45	postive
CLEC4E	FCER1G	0.612888955	4.76E-51	postive
CXCR2	FCER1G	0.531471662	1.72E-36	postive
ETS1	FCER1G	0.544377279	1.55E-38	postive
MCOLN1	FCER1G	0.515389569	4.64E-34	postive
GKN1	FCGBP	0.539381249	9.82E-38	postive
CD274	FCGR1B	0.550895116	1.32E-39	postive
CLEC4E	FCGR1B	0.536462914	2.85E-37	postive
CD274	FCGR2A	0.508874832	4.12E-33	postive
CLEC4E	FCGR2A	0.567684027	1.82E-42	postive
CXCR2	FCGR2A	0.540121428	7.48E-38	postive
ETS1	FCGR2A	0.53345667	8.45E-37	postive
MCOLN1	FCGR2A	0.514305064	6.70E-34	postive
ETS1	FCGR2B	0.536770321	2.55E-37	postive

CD274	FCGR2C	0.522895266	3.54E-35	postive
CLEC4E	FCGR2C	0.579222193	1.57E-44	postive
ETS1	FCGR2C	0.536569724	2.74E-37	postive
CD274	FCGR3B	0.537167169	2.21E-37	postive
CLEC4E	FCGR3B	0.69958215	4.48E-72	postive
CXCR2	FCGR3B	0.812543069	1.37E-114	postive
ETS1	FCHSD2	0.591673999	7.48E-47	postive
CLEC4E	FCN1	0.61208513	6.96E-51	postive
CXCR2	FCN1	0.60264343	5.54E-49	postive
ITGA5	FERMT2	0.625541308	1.05E-53	postive
ITGB1	FERMT2	0.548191725	3.69E-39	postive
ITGA5	FEZ1	0.506299836	9.64E-33	postive
CLEC4E	FFAR2	0.58219547	4.47E-45	postive
CXCR2	FFAR2	0.570764077	5.21E-43	postive
IL1B	FFAR2	0.514742726	5.78E-34	postive
ITGA5	FGF1	0.508132876	5.27E-33	postive
ITGA5	FGF2	0.610079176	1.79E-50	postive
ARRDC3	FGF7	0.545663642	9.56E-39	postive
ITGA5	FGF7	0.533281221	9.00E-37	postive
IL17RB	FGFR4	0.562020701	1.75E-41	postive
ARRDC3	FGL2	0.539801295	8.42E-38	postive
ETS1	FGL2	0.519800874	1.03E-34	postive
CD274	FGR	0.512529702	1.22E-33	postive
CLEC4E	FGR	0.754062407	1.13E-89	postive
CXCR2	FGR	0.687718891	9.24E-69	postive
ETS1	FGR	0.541288665	4.87E-38	postive
TRAF1	FGR	0.566188142	3.32E-42	postive
ITGA5	FHL3	0.516116331	3.63E-34	postive
ITGA5	FIBIN	0.604523891	2.34E-49	postive
ITGA5	FILIP1	0.578297359	2.31E-44	postive
ITGA5	FILIP1L	0.505572946	1.22E-32	postive
ITGA5	FKBP14	0.602767703	5.24E-49	postive
TRAP1	FKBP4	0.521770604	5.22E-35	postive
ARRDC3	FLI1	0.542389411	3.24E-38	postive
ETS1	FLI1	0.671340245	1.94E-64	postive
ITGA5	FLNA	0.65960253	1.65E-61	postive
ITGA5	FLNC	0.515950323	3.84E-34	postive
TRAF1	FMNL1	0.507031113	7.58E-33	postive
ETS1	FMNL3	0.54897121	2.75E-39	postive
ITGA5	FMNL3	0.586327729	7.64E-46	postive
TRAF1	FMNL3	0.502449213	3.39E-32	postive
ITGA5	FN1	0.630100185	1.08E-54	postive
ITGB1	FN1	0.514244889	6.83E-34	postive

LATS2	FN1	0.504325359	1.84E-32	postive
ITGA5	FNBP1L	-0.508154166	5.23E-33	negative
ITGA5	FNDC1	0.566588252	2.83E-42	postive
NR4A2	FOS	0.587926581	3.83E-46	postive
NR4A2	FOSB	0.623167953	3.37E-53	postive
PDIA3	FOXP2	-0.53255087	1.17E-36	negative
ADM	FPR1	0.524110221	2.32E-35	postive
CLEC4E	FPR1	0.753835765	1.36E-89	postive
CXCR2	FPR1	0.790518831	2.83E-104	postive
ETS1	FPR1	0.548915034	2.81E-39	postive
IL1B	FPR1	0.523022819	3.38E-35	postive
ADM	FPR2	0.578611346	2.03E-44	postive
CD274	FPR2	0.550809308	1.37E-39	postive
CLEC4E	FPR2	0.764111606	1.96E-93	postive
CXCR2	FPR2	0.693308743	2.66E-70	postive
IL1B	FPR2	0.651193415	1.73E-59	postive
CD274	FPR3	0.50516877	1.40E-32	postive
ETS1	FPR3	0.560869809	2.76E-41	postive
ITGA5	FRMD4A	0.522184226	4.52E-35	postive
ARRDC3	FRMD6	0.50913951	3.77E-33	postive
ITGA5	FRMD6	0.634958967	9.13E-56	postive
ITGB1	FRMD6	0.512154341	1.38E-33	postive
LATS2	FRMD6	0.500464	6.45E-32	postive
TLR5	FRY	0.503512405	2.40E-32	postive
ITGA5	FSTL1	0.657068995	6.82E-61	postive
LATS2	FSTL1	0.557086247	1.22E-40	postive
CEACAM6	FUT3	0.53972384	8.66E-38	postive
CEACAM6	FUT6	0.505050437	1.45E-32	postive
GKN1	FUT9	0.591866395	6.88E-47	postive
CD274	FYB	0.587021152	5.67E-46	postive
CLEC4E	FYB	0.577600073	3.10E-44	postive
ETS1	FYB	0.668021469	1.35E-63	postive
NLRC5	FYB	0.600682274	1.35E-48	postive
TRAF1	FYB	0.521980603	4.85E-35	postive
ITGB1	FYTTD1	0.554842653	2.90E-40	postive
ADM	G0S2	0.652563289	8.19E-60	postive
CLEC4E	G0S2	0.60295884	4.80E-49	postive
CXCR2	G0S2	0.638824263	1.24E-56	postive
IL1B	G0S2	0.675694709	1.47E-65	postive
ETS1	GAB3	0.528450807	5.04E-36	postive
ARRDC3	GABARAPL1	0.560758481	2.88E-41	postive
ARRDC3	GADD45B	0.525114022	1.63E-35	postive
LATS2	GADD45B	0.527462696	7.15E-36	postive

ITGA5	GALNT15	0.564982991	5.38E-42	postive
PRTG	GALNT16	0.544407803	1.53E-38	postive
CEACAM6	GALNT5	0.533529193	8.23E-37	postive
GKN1	GALNT6	0.551259434	1.15E-39	postive
ARRDC3	GAS1	0.506467384	9.12E-33	postive
ITGA5	GAS1	0.603366522	3.98E-49	postive
LATS2	GAS1	0.504657268	1.65E-32	postive
ITGA5	GAS7	0.503763792	2.21E-32	postive
CD274	GBP1	0.738005833	4.95E-84	postive
CLEC4E	GBP1	0.517551321	2.23E-34	postive
ETS1	GBP1	0.532855892	1.05E-36	postive
NLRC5	GBP1	0.679203615	1.77E-66	postive
CD274	GBP1P1	0.515381388	4.65E-34	postive
NLRC5	GBP2	0.573693992	1.57E-43	postive
CD274	GBP4	0.670309532	3.55E-64	postive
NLRC5	GBP4	0.703834339	2.65E-73	postive
CD274	GBP5	0.782203499	1.07E-100	postive
CLEC4E	GBP5	0.628791071	2.08E-54	postive
ETS1	GBP5	0.555533842	2.22E-40	postive
NLRC5	GBP5	0.671526809	1.74E-64	postive
HPSE	GCH1	0.525254746	1.55E-35	postive
GKN1	GCKR	0.509028682	3.91E-33	postive
CEACAM6	GCNT3	0.714751045	1.47E-76	postive
CEACAM6	GDA	0.60095909	1.19E-48	postive
PDIA3	GFPT1	0.538576575	1.32E-37	postive
ITGA5	GFPT2	0.672168292	1.19E-64	postive
LATS2	GFPT2	0.566187029	3.32E-42	postive
ITGA5	GGT5	0.570305225	6.28E-43	postive
ARRDC3	GGTA1P	0.509448458	3.41E-33	postive
GKN1	GHRL	0.551807613	9.33E-40	postive
GKN1	GIF	0.619141619	2.40E-52	postive
ETS1	GIMAP1	0.614454129	2.27E-51	postive
NLRC5	GIMAP2	0.522204266	4.49E-35	postive
ARRDC3	GIMAP4	0.533831379	7.39E-37	postive
ETS1	GIMAP4	0.643877203	8.78E-58	postive
NLRC5	GIMAP4	0.517715526	2.10E-34	postive
ARRDC3	GIMAP6	0.527306388	7.55E-36	postive
ETS1	GIMAP6	0.652100514	1.05E-59	postive
ARRDC3	GIMAP7	0.507078073	7.46E-33	postive
ETS1	GIMAP7	0.603041696	4.62E-49	postive
ETS1	GIMAP8	0.637100143	3.04E-56	postive
TRAF1	GIMAP8	0.506696175	8.46E-33	postive
IL17RB	GINS2	0.514219227	6.89E-34	postive

TRAP1	GINS2	0.510342021	2.53E-33	postive
ITGA5	GJA1	0.624727945	1.56E-53	postive
LATS2	GJA1	0.522059658	4.72E-35	postive
ITGA5	GJA4	0.538520335	1.35E-37	postive
ITGA5	GJC1	0.633458801	1.97E-55	postive
ADM	GK	0.525404905	1.47E-35	postive
CLEC4E	GK	0.526019451	1.19E-35	postive
IL1B	GK	0.640780241	4.48E-57	postive
ADM	GK3P	0.540505681	6.49E-38	postive
CLEC4E	GK3P	0.507897804	5.69E-33	postive
IL1B	GK3P	0.598248077	4.05E-48	postive
GKN1	GKN1	1	0	postive
GKN1	GKN2	0.974482506	1.32491212769672e-313	postive
ITGA5	GLI3	0.664658073	9.39E-63	postive
PDIA3	GLI3	-0.518822414	1.44E-34	negative
ARRDC3	GLIPR1	0.534004804	6.94E-37	postive
ETS1	GLIPR1	0.5406204	6.23E-38	postive
ITGA5	GLIPR1	0.5619858	1.77E-41	postive
ITGB1	GLIPR1	0.530816773	2.18E-36	postive
ARRDC3	GLIPR2	0.519598189	1.10E-34	postive
ETS1	GLIPR2	0.542624388	2.97E-38	postive
ITGA5	GLIPR2	0.609388364	2.47E-50	postive
LATS2	GLIS2	0.558515603	6.96E-41	postive
CLEC4E	GLT1D1	0.590284686	1.37E-46	postive
CXCR2	GLT1D1	0.602422566	6.13E-49	postive
ARRDC3	GLT8D2	0.501665402	4.37E-32	postive
ITGA5	GLT8D2	0.558040456	8.38E-41	postive
LATS2	GLT8D2	0.506251843	9.79E-33	postive
ITGB1	GLYCTK	-0.511599972	1.66E-33	negative
CLEC4E	GMFG	0.550071251	1.81E-39	postive
CXCR2	GMFG	0.502987659	2.85E-32	postive
ETS1	GMFG	0.69441956	1.30E-70	postive
TRAF1	GMFG	0.571359105	4.08E-43	postive
IL17RB	GMNN	0.503797609	2.19E-32	postive
ARRDC3	GNB4	0.507533584	6.42E-33	postive
ETS1	GNB4	0.560528635	3.16E-41	postive
ITGA5	GNB4	0.533889889	7.23E-37	postive
ITGA5	GNG11	0.593760954	2.99E-47	postive
ARRDC3	GNG2	0.523661319	2.71E-35	postive
ETS1	GNG2	0.618191168	3.79E-52	postive
TRAF1	GNG2	0.508617536	4.49E-33	postive
CD274	GNLY	0.633512707	1.91E-55	postive
NLRC5	GNLY	0.535710597	3.75E-37	postive

CEACAM6	GPA33	0.554555699	3.24E-40	postive
ITGA5	GPC6	0.507393618	6.72E-33	postive
ITGA5	GPM6B	0.543698689	1.99E-38	postive
PDIA3	GPM6B	-0.500748306	5.88E-32	negative
ETS1	GPR116	0.559448354	4.83E-41	postive
ITGA5	GPR116	0.606730176	8.48E-50	postive
ITGA5	GPR124	0.623563631	2.78E-53	postive
LATS2	GPR124	0.508516577	4.64E-33	postive
CD274	GPR171	0.517539295	2.23E-34	postive
ETS1	GPR171	0.560167253	3.64E-41	postive
NLRC5	GPR171	0.634143954	1.39E-55	postive
TRAF1	GPR171	0.521274429	6.20E-35	postive
ITGA5	GPR176	0.516152823	3.58E-34	postive
NLRC5	GPR18	0.546370863	7.33E-39	postive
TRAF1	GPR18	0.503133627	2.71E-32	postive
ARRDC3	GPR183	0.53169419	1.59E-36	postive
ETS1	GPR183	0.572237434	2.85E-43	postive
TRAF1	GPR183	0.507470743	6.55E-33	postive
HIF1A	GPR4	0.565120915	5.09E-42	postive
ITGA5	GPR4	0.526690165	9.38E-36	postive
GKN1	GPR64	0.652462968	8.65E-60	postive
CD274	GPR65	0.565801216	3.88E-42	postive
CLEC4E	GPR65	0.622043783	5.85E-53	postive
CXCR2	GPR65	0.57066288	5.43E-43	postive
ETS1	GPR65	0.629453439	1.49E-54	postive
ADM	GPR84	0.557250413	1.14E-40	postive
CD274	GPR84	0.551731422	9.60E-40	postive
CLEC4E	GPR84	0.657288703	6.03E-61	postive
CXCR2	GPR84	0.514849866	5.57E-34	postive
HIF1A	GPR84	0.509035017	3.91E-33	postive
IL1B	GPR84	0.603066754	4.57E-49	postive
CEACAM3	GPR97	0.542359937	3.27E-38	postive
CLEC4E	GPR97	0.630621893	8.28E-55	postive
CXCR2	GPR97	0.670665705	2.88E-64	postive
PDIA3	GPRASP1	-0.518906061	1.40E-34	negative
TRAF1	GPSM3	0.607543754	5.82E-50	postive
ITGA5	GPX8	0.525832417	1.27E-35	postive
LATS2	GPX8	0.513190391	9.75E-34	postive
CEACAM6	GSKIP	0.550028228	1.84E-39	postive
ARRDC3	GSS	-0.532180763	1.34E-36	negative
GKN1	GSTA1	0.566067329	3.48E-42	postive
ITGA5	GUCY1A2	0.511822722	1.54E-33	postive
ITGA5	GUCY1A3	0.525189074	1.59E-35	postive

ARRDC3	GUCY1B3	0.535548496	3.97E-37	postive
ITGA5	GUCY1B3	0.570303192	6.28E-43	postive
ITGB1	GUCY1B3	0.539944666	7.98E-38	postive
CEACAM6	GUCY2C	0.571932146	3.23E-43	postive
ETS1	GVINP1	0.552698067	6.63E-40	postive
NLRC5	GVINP1	0.627797836	3.41E-54	postive
TRAF1	GVINP1	0.575023694	9.04E-44	postive
ITGB1	GYG1	0.547710253	4.43E-39	postive
ITGA5	GYPC	0.568392014	1.37E-42	postive
CD274	GZMA	0.569972215	7.19E-43	postive
NLRC5	GZMA	0.638503335	1.47E-56	postive
CD274	GZMB	0.723506002	2.78E-79	postive
CLEC4E	GZMB	0.560337418	3.40E-41	postive
NLRC5	GZMB	0.583238528	2.87E-45	postive
CD274	GZMH	0.574844283	9.73E-44	postive
ETS1	GZMH	0.501247219	5.01E-32	postive
NLRC5	GZMH	0.608907728	3.09E-50	postive
NLRC5	GZMK	0.503064164	2.78E-32	postive
CD274	HAPLN3	0.61298632	4.55E-51	postive
NLRC5	HAPLN3	0.523276301	3.10E-35	postive
CD274	HAVCR2	0.597275073	6.26E-48	postive
CLEC4E	HAVCR2	0.514882403	5.51E-34	postive
ETS1	HAVCR2	0.561358064	2.27E-41	postive
NR4A2	HBEGF	0.546105089	8.10E-39	postive
ADM	HCAR3	0.634996794	8.96E-56	postive
CD274	HCAR3	0.548760874	2.98E-39	postive
CLEC4E	HCAR3	0.659050674	2.25E-61	postive
CXCR2	HCAR3	0.676738821	7.84E-66	postive
IL1B	HCAR3	0.713474118	3.60E-76	postive
NLRC5	HCG26	0.561345419	2.29E-41	postive
CD274	HCK	0.61074865	1.31E-50	postive
CLEC4E	HCK	0.688691336	5.01E-69	postive
CXCR2	HCK	0.615376496	1.46E-51	postive
ETS1	HCK	0.583864984	2.20E-45	postive
TRAF1	HCK	0.518829617	1.44E-34	postive
CLEC4E	HCLS1	0.502012089	3.91E-32	postive
ETS1	HCLS1	0.647434615	1.32E-58	postive
NLRC5	HCLS1	0.542354705	3.28E-38	postive
TRAF1	HCLS1	0.607843919	5.06E-50	postive
NLRC5	HCP5	0.545115076	1.17E-38	postive
CD274	HCST	0.522199229	4.50E-35	postive
CLEC4E	HCST	0.517918427	1.96E-34	postive
ETS1	HCST	0.608838707	3.19E-50	postive

NLRC5	HCST	0.562446459	1.48E-41	postive
TRAF1	HCST	0.57029539	6.30E-43	postive
ARRDC3	HDGF	-0.505463249	1.27E-32	negative
HIF1A	HECW2	0.517338468	2.39E-34	postive
ETS1	HEG1	0.515765795	4.09E-34	postive
ITGA5	HEG1	0.641009922	3.98E-57	postive
LATS2	HEG1	0.594849139	1.84E-47	postive
GKN1	HHIP	0.547633086	4.56E-39	postive
CD274	HK3	0.596724119	8.01E-48	postive
CLEC4E	HK3	0.641530877	3.03E-57	postive
NLRC5	HLA-DMA	0.596129473	1.04E-47	postive
NLRC5	HLA-DMB	0.594645371	2.02E-47	postive
NLRC5	HLA-DOA	0.521710929	5.33E-35	postive
NLRC5	HLA-DPA1	0.537696337	1.82E-37	postive
NLRC5	HLA-DPB1	0.581807802	5.27E-45	postive
NLRC5	HLA-DRA	0.539403916	9.74E-38	postive
NLRC5	HLA-DRB6	0.528663774	4.68E-36	postive
NLRC5	HLA-F	0.677494379	4.97E-66	postive
NLRC5	HLA-J	0.560757662	2.88E-41	postive
ETS1	HLX	0.551790433	9.39E-40	postive
ITGA5	HLX	0.560586362	3.08E-41	postive
TRAP1	HMGA1	0.573887672	1.45E-43	postive
ADM	HMOX1	0.511789304	1.56E-33	postive
CLEC4E	HMOX1	0.504162425	1.94E-32	postive
CXCR2	HMOX1	0.502694474	3.13E-32	postive
USF1	HNRNPL	0.585777882	9.68E-46	postive
ITGA5	HOMER3	0.518207698	1.78E-34	postive
IL17RB	HOOK1	0.5268527	8.86E-36	postive
ITGA5	HOOK1	-0.578507129	2.12E-44	negative
GKN1	HPGD	0.589919986	1.61E-46	postive
HPSE	HPSE	1	0	postive
GKN1	HRASLS2	0.602880078	4.97E-49	postive
ITGA5	HS3ST3A1	0.514146077	7.07E-34	postive
CD274	HS3ST3B1	0.529754688	3.18E-36	postive
CLEC4E	HS3ST3B1	0.547079823	5.61E-39	postive
ETS1	HS3ST3B1	0.508310448	4.97E-33	postive
HIF1A	HS3ST3B1	0.507986803	5.53E-33	postive
TRAF1	HS3ST3B1	0.543369564	2.25E-38	postive
ETS1	HSD11B1	0.522264172	4.40E-35	postive
ITGA5	HSD11B1	0.502969324	2.86E-32	postive
NLRC5	HSH2D	0.521005069	6.80E-35	postive
ITGA5	HSPA12B	0.572217258	2.87E-43	postive
ITGA5	HSPA2	0.517563742	2.22E-34	postive

ADM	HSPA6	0.534271205	6.30E-37	postive
CLEC4E	HSPA6	0.532888196	1.04E-36	postive
ITGA5	HSPB2	0.555130798	2.60E-40	postive
LATS2	HSPB2	0.504313195	1.85E-32	postive
ITGA5	HSPB8	0.501642642	4.40E-32	postive
ITGA5	HSPG2	0.619692206	1.84E-52	postive
LATS2	HSPG2	0.523242423	3.13E-35	postive
ITGA5	HTRA1	0.526198107	1.12E-35	postive
ITGA5	HTRA3	0.544672128	1.38E-38	postive
ETS1	HVCN1	0.515700182	4.18E-34	postive
CD274	ICAM1	0.662004842	4.26E-62	postive
CLEC4E	ICAM1	0.575928423	6.21E-44	postive
ETS1	ICAM1	0.511811627	1.55E-33	postive
CD274	ICOS	0.594258962	2.40E-47	postive
ETS1	ICOS	0.560532969	3.15E-41	postive
NLRC5	ICOS	0.530350111	2.57E-36	postive
TRAF1	ICOS	0.555860395	1.96E-40	postive
CD274	IDO1	0.747048784	3.71E-87	postive
CLEC4E	IDO1	0.510393902	2.49E-33	postive
NLRC5	IDO1	0.630274912	9.86E-55	postive
IL1B	IER3	0.52085209	7.17E-35	postive
ETS1	IFFO1	0.535957172	3.43E-37	postive
ITGA5	IFFO1	0.569213242	9.79E-43	postive
ETS1	IFI16	0.56123342	2.39E-41	postive
NLRC5	IFI16	0.563909045	8.26E-42	postive
NLRC5	IFI44	0.533892565	7.23E-37	postive
CD274	IFIH1	0.571157153	4.43E-43	postive
NLRC5	IFIH1	0.527254631	7.69E-36	postive
CD274	IFIT3	0.562192577	1.64E-41	postive
NLRC5	IFIT3	0.57610166	5.78E-44	postive
NLRC5	IFIT5	0.516899685	2.78E-34	postive
NLRC5	IFITM1	0.523118125	3.27E-35	postive
CD274	IFNG	0.687122013	1.34E-68	postive
NLRC5	IFNG	0.553453144	4.96E-40	postive
TLR5	IGF1	0.502960865	2.87E-32	postive
ITGA5	IGFBP4	0.639630778	8.17E-57	postive
ITGA5	IGFBP7	0.591202446	9.20E-47	postive
CD274	IGFLR1	0.543764775	1.94E-38	postive
ETS1	IGFLR1	0.503683041	2.27E-32	postive
NLRC5	IGFLR1	0.502196208	3.68E-32	postive
GKN1	IGJ	0.586800304	6.23E-46	postive
CD274	IGSF6	0.582321524	4.24E-45	postive
CLEC4E	IGSF6	0.633416256	2.01E-55	postive

CXCR2	IGSF6	0.576776074	4.36E-44	postive
ETS1	IGSF6	0.577733385	2.93E-44	postive
ETS1	IKBIP	0.5609483	2.67E-41	postive
ETS1	IKZF1	0.590001138	1.56E-46	postive
NLRC5	IKZF1	0.59454805	2.11E-47	postive
TRAF1	IKZF1	0.614351643	2.38E-51	postive
NLRC5	IKZF3	0.59393078	2.77E-47	postive
ETS1	IL10RA	0.65463491	2.63E-60	postive
NLRC5	IL10RA	0.553378562	5.11E-40	postive
TRAF1	IL10RA	0.571399311	4.02E-43	postive
CLEC4E	IL10RB-AS1	0.504895238	1.53E-32	postive
HIF1A	IL11	0.561676944	2.01E-41	postive
IL1B	IL11	0.577083466	3.84E-44	postive
MMP10	IL11	0.587796046	4.05E-46	postive
MMP10	IL13RA2	0.622123816	5.62E-53	postive
NLRC5	IL15	0.563570731	9.46E-42	postive
CD274	IL15RA	0.542729472	2.85E-38	postive
NLRC5	IL15RA	0.526928203	8.63E-36	postive
NLRC5	IL16	0.512285846	1.32E-33	postive
TRAF1	IL16	0.57219296	2.90E-43	postive
IL17RB	IL17RB	1	0	postive
CD274	IL18RAP	0.58345695	2.61E-45	postive
CLEC4E	IL18RAP	0.559184455	5.35E-41	postive
ETS1	IL18RAP	0.54346483	2.17E-38	postive
NLRC5	IL18RAP	0.569207111	9.81E-43	postive
TRAF1	IL18RAP	0.555401261	2.34E-40	postive
ADM	IL1A	0.560574667	3.10E-41	postive
CLEC4E	IL1A	0.525991691	1.20E-35	postive
IL1B	IL1A	0.751321219	1.11E-88	postive
ADM	IL1B	0.600193634	1.68E-48	postive
CLEC4E	IL1B	0.515554089	4.39E-34	postive
CXCR2	IL1B	0.554707788	3.06E-40	postive
IL1B	IL1B	1	0	postive
MMP10	IL1B	0.559735917	4.31E-41	postive
ITGA5	IL1R1	0.556790317	1.36E-40	postive
LATS2	IL1R1	0.60233167	6.39E-49	postive
IL1B	IL1RN	0.656998529	7.09E-61	postive
CD274	IL21R	0.611596073	8.76E-51	postive
ETS1	IL21R	0.566318609	3.15E-42	postive
NLRC5	IL21R	0.603159971	4.38E-49	postive
TRAF1	IL21R	0.631556037	5.16E-55	postive
CEACAM1	IL22RA1	0.5006018	6.16E-32	postive
HIF1A	IL24	0.545251245	1.12E-38	postive

IL1B	IL24	0.544954989	1.25E-38	postive
MMP10	IL24	0.544853934	1.29E-38	postive
CD274	IL2RA	0.668400995	1.08E-63	postive
CLEC4E	IL2RA	0.591943454	6.65E-47	postive
ETS1	IL2RA	0.581377675	6.32E-45	postive
NLRC5	IL2RA	0.545025794	1.21E-38	postive
TRAF1	IL2RA	0.528659305	4.68E-36	postive
CD274	IL2RB	0.621577878	7.34E-53	postive
ETS1	IL2RB	0.5903797	1.32E-46	postive
NLRC5	IL2RB	0.681353735	4.78E-67	postive
TRAF1	IL2RB	0.583639097	2.42E-45	postive
ETS1	IL3RA	0.546015152	8.38E-39	postive
ITGA5	IL3RA	0.588396655	3.12E-46	postive
CD274	IL4I1	0.583958879	2.11E-45	postive
CLEC4E	IL4I1	0.519934884	9.84E-35	postive
ADM	IL6	0.569092504	1.03E-42	postive
IL1B	IL6	0.540300958	7.00E-38	postive
ARRDC3	IL6ST	0.550771592	1.39E-39	postive
ETS1	IL6ST	0.559557324	4.62E-41	postive
ETS1	IL7R	0.589177397	2.23E-46	postive
TRAF1	IL7R	0.557885744	8.90E-41	postive
ITGA5	ILK	0.51239461	1.27E-33	postive
GKN1	INSC	0.536182726	3.16E-37	postive
ETS1	IPCEF1	0.501408962	4.75E-32	postive
TRAP1	IPO4	0.50335197	2.53E-32	postive
CD274	IRF1	0.666528984	3.20E-63	postive
NLRC5	IRF1	0.695662816	5.81E-71	postive
TRAF1	IRF4	0.504346649	1.83E-32	postive
ITGA5	ISLR	0.52727709	7.63E-36	postive
ITGA5	ITGA1	0.563240647	1.08E-41	postive
ETS1	ITGA4	0.672065891	1.27E-64	postive
ITGA5	ITGA5	1	0	postive
LATS2	ITGA5	0.56916819	9.97E-43	postive
ITGA5	ITGA7	0.545526686	1.01E-38	postive
PDIA3	ITGA7	-0.516130212	3.61E-34	negative
CD274	ITGAL	0.552955416	6.01E-40	postive
ETS1	ITGAL	0.617044152	6.58E-52	postive
NLRC5	ITGAL	0.63634535	4.48E-56	postive
TRAF1	ITGAL	0.62245494	4.78E-53	postive
CLEC4E	ITGAM	0.599017251	2.86E-48	postive
ETS1	ITGAM	0.564808118	5.77E-42	postive
TRAF1	ITGAM	0.514564776	6.13E-34	postive
CD274	ITGAX	0.5273516	7.43E-36	postive

CLEC4E	ITGAX	0.617786237	4.61E-52	postive
CXCR2	ITGAX	0.525087674	1.65E-35	postive
ETS1	ITGAX	0.511381413	1.79E-33	postive
TRAF1	ITGAX	0.527601258	6.81E-36	postive
CD274	ITGB2	0.569930544	7.31E-43	postive
CLEC4E	ITGB2	0.575541093	7.29E-44	postive
ETS1	ITGB2	0.606752306	8.40E-50	postive
NLRC5	ITGB7	0.535979168	3.40E-37	postive
ETS1	ITK	0.577589694	3.11E-44	postive
NLRC5	ITK	0.603760798	3.33E-49	postive
TRAF1	ITK	0.56243219	1.49E-41	postive
ARRDC3	ITPR1	0.549535803	2.22E-39	postive
ARRDC3	ITPR3	-0.500148281	7.14E-32	negative
CLEC4E	ITPRIP	0.503178716	2.68E-32	postive
ETS1	ITPRIP	0.578476016	2.15E-44	postive
HIF1A	ITPRIP	0.533261929	9.07E-37	postive
ITGA5	ITPRIP	0.551623633	1.00E-39	postive
TRAF1	ITPRIP	0.543248401	2.35E-38	postive
CD274	JAK2	0.601341505	1.00E-48	postive
NLRC5	JAK2	0.523131818	3.26E-35	postive
NLRC5	JAKMIP1	0.512603232	1.19E-33	postive
TLR5	JAM2	0.524175763	2.26E-35	postive
NR4A2	JUNB	0.576482026	4.93E-44	postive
ITGA5	KANK2	0.508191221	5.17E-33	postive
ITGA5	KANK3	0.519465387	1.16E-34	postive
ITGA5	KATNAL1	0.524522441	2.01E-35	postive
GKN1	KCNE2	0.608555733	3.64E-50	postive
ARRDC3	KCNE4	0.523168056	3.22E-35	postive
ITGA5	KCNE4	0.505629136	1.20E-32	postive
ARRDC3	KCNJ8	0.511228257	1.88E-33	postive
ITGA5	KCNJ8	0.594994823	1.73E-47	postive
ITGA5	KCNMB1	0.54113466	5.15E-38	postive
ITGA5	KDF1	-0.519729152	1.06E-34	negative
ITGA5	KDR	0.503143009	2.71E-32	postive
ITGA5	KGFLP2	0.509442148	3.41E-33	postive
TRAF1	KIAA0226L	0.5211053	6.57E-35	postive
ITGA5	KIAA1462	0.565547387	4.29E-42	postive
LATS2	KIAA1462	0.525263821	1.55E-35	postive
ITGA5	KIAA1644	0.585968418	8.92E-46	postive
ITGA5	KIAA1804	-0.543586818	2.08E-38	negative
IL17RB	KIF15	0.532783267	1.08E-36	postive
IL17RB	KIF18A	0.504455637	1.76E-32	postive
IL17RB	KIF2C	0.502649937	3.18E-32	postive

TLR5	KIF2C	-0.518840544	1.43E-34	negative
CD274	KIR2DL4	0.521752686	5.25E-35	postive
ITGA5	KIRREL	0.637048898	3.12E-56	postive
LATS2	KIRREL	0.565663795	4.10E-42	postive
TLR5	KLHDC1	0.536777186	2.54E-37	postive
CD274	KLHDC7B	0.500691929	5.99E-32	postive
ITGA5	KLHL5	0.643859488	8.87E-58	postive
LATS2	KLHL5	0.54866777	3.08E-39	postive
CD274	KLHL6	0.523777164	2.60E-35	postive
ETS1	KLHL6	0.618556966	3.18E-52	postive
NLRC5	KLHL6	0.574204923	1.27E-43	postive
TRAF1	KLHL6	0.590942561	1.03E-46	postive
NLRC5	KLRC3	0.527064357	8.23E-36	postive
CD274	KLRD1	0.651559476	1.42E-59	postive
CLEC4E	KLRD1	0.548634789	3.12E-39	postive
ETS1	KLRD1	0.505421667	1.29E-32	postive
NLRC5	KLRD1	0.553302043	5.26E-40	postive
CD274	KMO	0.593173988	3.87E-47	postive
CLEC4E	KMO	0.577688404	2.98E-44	postive
TLR5	KPNA2	-0.500195207	7.03E-32	negative
CEACAM1	KRT18	0.504813369	1.57E-32	postive
CEACAM6	KRT19	0.516813937	2.86E-34	postive
CEACAM6	KRT20	0.544589739	1.43E-38	postive
GKN1	KRT20	0.508522246	4.63E-33	postive
CEACAM1	KRT8	0.505277344	1.35E-32	postive
CEACAM6	KRT8	0.538608681	1.30E-37	postive
ITGA5	L3HYPDH	0.514426022	6.43E-34	postive
ITGB1	L3HYPDH	0.527591931	6.83E-36	postive
HPSE	LACTB	0.59817064	4.19E-48	postive
CD274	LAG3	0.552220512	7.96E-40	postive
NLRC5	LAG3	0.549574521	2.19E-39	postive
CD274	LAIR1	0.541484603	4.53E-38	postive
CLEC4E	LAIR1	0.520159234	9.10E-35	postive
ETS1	LAIR1	0.597482237	5.70E-48	postive
ITGA5	LAMA4	0.718943031	7.53E-78	postive
LATS2	LAMA4	0.58097276	7.50E-45	postive
ITGA5	LAMB1	0.541558924	4.40E-38	postive
ITGA5	LAMB2	0.574871377	9.63E-44	postive
LATS2	LAMB2	0.529822655	3.10E-36	postive
ITGA5	LAMC1	0.59697862	7.15E-48	postive
LATS2	LAMC1	0.591547487	7.91E-47	postive
CD274	LAMP3	0.602577266	5.71E-49	postive
NLRC5	LAMP3	0.570677368	5.39E-43	postive

TRAF1	LAMP3	0.501602906	4.46E-32	postive
CD274	LAP3	0.605742366	1.34E-49	postive
NLRC5	LAP3	0.583535569	2.53E-45	postive
CD274	LAPTM5	0.56766634	1.83E-42	postive
CLEC4E	LAPTM5	0.56582551	3.84E-42	postive
ETS1	LAPTM5	0.640268136	5.86E-57	postive
TRAF1	LAT	0.519836963	1.02E-34	postive
CD274	LAT2	0.512246786	1.34E-33	postive
CLEC4E	LAT2	0.571158066	4.43E-43	postive
CXCR2	LAT2	0.520694397	7.57E-35	postive
ETS1	LAT2	0.543715882	1.98E-38	postive
TRAF1	LAT2	0.506399495	9.33E-33	postive
ITGA5	LAYN	0.604637234	2.23E-49	postive
NLRC5	LCK	0.508816697	4.20E-33	postive
CD274	LCP1	0.626684823	5.94E-54	postive
CLEC4E	LCP1	0.660849406	8.19E-62	postive
CXCR2	LCP1	0.537630728	1.86E-37	postive
ETS1	LCP1	0.653590962	4.66E-60	postive
NLRC5	LCP1	0.530715477	2.26E-36	postive
TRAF1	LCP1	0.616941007	6.91E-52	postive
CD274	LCP2	0.664046276	1.33E-62	postive
CLEC4E	LCP2	0.737309609	8.51E-84	postive
CXCR2	LCP2	0.62897446	1.89E-54	postive
ETS1	LCP2	0.66401474	1.36E-62	postive
IL1B	LCP2	0.510770559	2.19E-33	postive
NLRC5	LCP2	0.525827763	1.27E-35	postive
TRAF1	LCP2	0.633915474	1.56E-55	postive
ITGA5	LEPRE1	0.536895911	2.44E-37	postive
ITGA5	LGALS1	0.718519527	1.02E-77	postive
ITGB1	LGALS1	0.572953455	2.12E-43	postive
LATS2	LGALS1	0.537761835	1.78E-37	postive
CEACAM6	LGALS4	0.559106645	5.52E-41	postive
ITGA5	LHFP	0.538665012	1.28E-37	postive
CLEC4E	LILRA2	0.691973556	6.25E-70	postive
CXCR2	LILRA2	0.711938574	1.05E-75	postive
CEACAM3	LILRA5	0.52861547	4.76E-36	postive
CLEC4E	LILRA5	0.663272032	2.07E-62	postive
CXCR2	LILRA5	0.625911403	8.72E-54	postive
CLEC4E	LILRA6	0.556584428	1.48E-40	postive
CXCR2	LILRA6	0.502501412	3.33E-32	postive
CD274	LILRB1	0.608867981	3.14E-50	postive
CLEC4E	LILRB1	0.622178976	5.47E-53	postive
CXCR2	LILRB1	0.502980822	2.85E-32	postive

ETS1	LILRB1	0.569412966	9.02E-43	postive
CD274	LILRB2	0.526776039	9.10E-36	postive
CLEC4E	LILRB2	0.705413347	9.16E-74	postive
CXCR2	LILRB2	0.655465089	1.66E-60	postive
ETS1	LILRB2	0.608346001	4.01E-50	postive
ITGA5	LILRB2	0.528988027	4.17E-36	postive
TRAF1	LILRB2	0.506432993	9.23E-33	postive
CD274	LILRB3	0.575128985	8.65E-44	postive
CLEC4E	LILRB3	0.70712444	2.87E-74	postive
CXCR2	LILRB3	0.59823951	4.06E-48	postive
ETS1	LILRB3	0.547323218	5.12E-39	postive
TRAF1	LILRB3	0.559725893	4.33E-41	postive
CD274	LILRB4	0.627840083	3.34E-54	postive
CLEC4E	LILRB4	0.588032692	3.66E-46	postive
ITGA5	LIMS2	0.540758145	5.92E-38	postive
PDIA3	LIMS2	-0.502877305	2.95E-32	negative
GKN1	LINC00261	0.516048965	3.71E-34	postive
PDIA3	LINC00478	-0.530543523	2.40E-36	negative
CEACAM6	LINC00483	0.595714063	1.26E-47	postive
GKN1	LINC00675	0.555362111	2.37E-40	postive
GKN1	LINC01133	0.505781516	1.14E-32	postive
ITGA5	LINC01279	0.505043378	1.46E-32	postive
GKN1	LIPF	0.707184193	2.76E-74	postive
ITGA5	LIX1L	0.520162856	9.09E-35	postive
TRAP1	LMNB2	0.510587964	2.33E-33	postive
CEACAM6	LMO7	0.512598326	1.19E-33	postive
ITGA5	LOC100132891	0.550987496	1.28E-39	postive
ITGA5	LOC100287387	0.529169009	3.91E-36	postive
ETS1	LOC100505812	0.558479421	7.06E-41	postive
CD274	LOC101928173	0.598313979	3.93E-48	postive
CLEC4E	LOC101928173	0.549666744	2.11E-39	postive
ETS1	LOC101928173	0.536952349	2.39E-37	postive
TRAF1	LOC101928173	0.512649781	1.17E-33	postive
ETS1	LOC101928370	0.508137543	5.26E-33	postive
PDIA3	LOC102724362	0.505896136	1.10E-32	postive
ETS1	LOC374443	0.562471101	1.46E-41	postive
ARRDC3	LOC643733	0.500753969	5.87E-32	postive
ETS1	LOC643733	0.520088837	9.33E-35	postive
CLEC4E	LOC731424	0.56802319	1.58E-42	postive
IL1B	LOC731424	0.591983	6.53E-47	postive
ITGA5	LOX	0.544864786	1.29E-38	postive
ITGA5	LOXL2	0.554209115	3.71E-40	postive
TRAP1	LPAR1	-0.513293706	9.42E-34	negative

CD274	LPXN	0.504769786	1.59E-32	postive
ETS1	LPXN	0.539795946	8.43E-38	postive
NLRC5	LPXN	0.562584671	1.40E-41	postive
TRAF1	LPXN	0.588222479	3.37E-46	postive
ITGA5	LRCH2	0.506333254	9.53E-33	postive
ETS1	LRMP	0.506108227	1.03E-32	postive
ITGA5	LRRC32	0.530196688	2.71E-36	postive
LATS2	LRRC32	0.502255152	3.61E-32	postive
GKN1	LRRC66	0.544125333	1.70E-38	postive
ARRDC3	LRRK2	0.548211718	3.66E-39	postive
ETS1	LRRK2	0.548963283	2.76E-39	postive
TRAF1	LSP1	0.569130247	1.01E-42	postive
CD274	LST1	0.535542685	3.98E-37	postive
CLEC4E	LST1	0.657180497	6.41E-61	postive
CXCR2	LST1	0.611837055	7.82E-51	postive
ETS1	LST1	0.579187752	1.59E-44	postive
TRAF1	LST1	0.530093891	2.81E-36	postive
ITGA5	LTBP1	0.525860397	1.26E-35	postive
GKN1	LTF	0.696120662	4.32E-71	postive
ITGA5	LUM	0.506495673	9.04E-33	postive
ETS1	LY86	0.525272668	1.54E-35	postive
ARRDC3	LY96	0.540678577	6.09E-38	postive
ETS1	LY96	0.60309966	4.50E-49	postive
ITGA5	LYVE1	0.518928008	1.39E-34	postive
ETS1	MAFB	0.579291642	1.52E-44	postive
PDIA3	MAGI2	-0.516401921	3.29E-34	negative
ITGA5	MAGI2-AS3	0.570697967	5.35E-43	postive
PDIA3	MAGI2-AS3	-0.517509937	2.26E-34	negative
GKN1	MAL	0.578264662	2.34E-44	postive
CEACAM6	MALL	0.535279207	4.38E-37	postive
PDIA3	MANF	0.530708907	2.26E-36	postive
PDIA3	MAP1A	-0.537744731	1.79E-37	negative
ITGA5	MAP1B	0.586333786	7.62E-46	postive
ETS1	MAP3K7CL	0.516744853	2.93E-34	postive
NLRC5	MAP4K1	0.550274075	1.67E-39	postive
TRAF1	MAP4K1	0.58541857	1.13E-45	postive
CEACAM1	MAP7	0.501289775	4.94E-32	postive
ITGA5	MAP7D1	0.650158764	3.03E-59	postive
ETS1	1-Mar	0.645745057	3.26E-58	postive
NLRC5	1-Mar	0.503114504	2.73E-32	postive
ITGA5	MARVELD1	0.572808261	2.25E-43	postive
LATS2	MARVELD1	0.550634889	1.46E-39	postive
ITGA5	MARVELD2	-0.552348379	7.58E-40	negative

CEACAM1	MARVELD3	0.517069253	2.62E-34	postive
ITGA5	MBNL1-AS1	0.505061862	1.45E-32	postive
ITGA5	MCAM	0.73154071	7.09E-82	postive
LATS2	MCAM	0.539545867	9.24E-38	postive
ITGA5	MCC	0.548585596	3.18E-39	postive
ADM	MCEMP1	0.593081779	4.03E-47	postive
CLEC4E	MCEMP1	0.635127721	8.38E-56	postive
CXCR2	MCEMP1	0.606443369	9.69E-50	postive
HIF1A	MCEMP1	0.53194771	1.45E-36	postive
IL1B	MCEMP1	0.587953381	3.79E-46	postive
TRAP1	MCM2	0.510301917	2.56E-33	postive
ETS1	MCTP1	0.529520351	3.45E-36	postive
ARRDC3	MDFIC	0.521643689	5.46E-35	postive
ETS1	MDFIC	0.585282448	1.20E-45	postive
ITGA5	MEDAG	0.541406115	4.66E-38	postive
ETS1	MEF2C	0.535451994	4.11E-37	postive
TRAF1	MEI1	0.530495574	2.44E-36	postive
ITGA5	MEOX1	0.522254812	4.41E-35	postive
ITGA5	MEOX2	0.531804923	1.53E-36	postive
CEACAM6	MEP1A	0.514208543	6.92E-34	postive
ITGA5	MFGE8	0.526657141	9.49E-36	postive
ITGA5	MGC24103	0.521401286	5.93E-35	postive
NLRC5	MIAT	0.561359701	2.27E-41	postive
TRAF1	MIAT	0.528574301	4.83E-36	postive
ITGA5	MICAL2	0.504916554	1.52E-32	postive
CD274	MICB	0.548138215	3.77E-39	postive
TRAP1	MIPEP	0.517073085	2.62E-34	postive
CLEC4E	mir-223	0.606652775	8.79E-50	postive
CXCR2	mir-223	0.666794384	2.75E-63	postive
ITGA5	MIR100HG	0.535608049	3.89E-37	postive
ITGA5	MIR143HG	0.500033835	7.40E-32	postive
PDIA3	MIR143HG	-0.514212269	6.91E-34	negative
CEACAM6	MISP	0.57251193	2.55E-43	postive
USF1	MLH1	-0.587375015	4.86E-46	negative
NLRC5	MLKL	0.541584268	4.36E-38	postive
IL1B	MMP1	0.556115977	1.77E-40	postive
MMP10	MMP1	0.686570918	1.90E-68	postive
IL1B	MMP10	0.559735926	4.31E-41	postive
MMP10	MMP10	1	0	postive
IL1B	MMP12	0.617224706	6.03E-52	postive
MMP10	MMP12	0.624752145	1.55E-53	postive
ETS1	MMP19	0.510165362	2.68E-33	postive
ITGA5	MMP19	0.536873218	2.46E-37	postive

TRAF1	MMP19	0.556944941	1.28E-40	postive
ITGA5	MMP2	0.601167261	1.08E-48	postive
LATS2	MMP2	0.517092284	2.60E-34	postive
IL1B	MMP3	0.628399284	2.53E-54	postive
MMP10	MMP3	0.73168136	6.38E-82	postive
ADM	MMP8	0.515269781	4.83E-34	postive
CLEC4E	MMP8	0.512485473	1.24E-33	postive
CD274	MMP9	0.53427805	6.29E-37	postive
CLEC4E	MMP9	0.576541582	4.81E-44	postive
IL1B	MMP9	0.504719008	1.62E-32	postive
TRAF1	MMP9	0.517568157	2.21E-34	postive
ETS1	MMRN2	0.509616815	3.22E-33	postive
ITGA5	MMRN2	0.55900978	5.73E-41	postive
CD274	MNDA	0.522017301	4.79E-35	postive
CLEC4E	MNDA	0.674772202	2.54E-65	postive
CXCR2	MNDA	0.744059964	4.14E-86	postive
ETS1	MNDA	0.581047175	7.27E-45	postive
ITGA5	MPDZ	0.577807436	2.84E-44	postive
ETS1	MPEG1	0.587552627	4.50E-46	postive
ITGA5	MRAS	0.579561144	1.36E-44	postive
LATS2	MRAS	0.522577797	3.95E-35	postive
ETS1	MRC1	0.537556783	1.91E-37	postive
ITGA5	MRC2	0.664857053	8.37E-63	postive
LATS2	MRC2	0.566126968	3.40E-42	postive
ITGA5	MRGPRF	0.617710412	4.78E-52	postive
TRAP1	MRPS12	0.50398753	2.06E-32	postive
TRAP1	MRTO4	0.518268921	1.74E-34	postive
ETS1	MS4A4A	0.532823458	1.06E-36	postive
ETS1	MS4A6A	0.515692788	4.19E-34	postive
ETS1	MS4A7	0.608482691	3.76E-50	postive
GKN1	MSMB	0.588871916	2.54E-46	postive
ETS1	MSN	0.597974931	4.57E-48	postive
ITGA5	MSN	0.626916599	5.29E-54	postive
LATS2	MSN	0.507216416	7.13E-33	postive
ITGA5	MSRB3	0.609126669	2.79E-50	postive
ITGB1	MSRB3	0.546728383	6.41E-39	postive
CEACAM1	MUC13	0.575442166	7.60E-44	postive
CEACAM6	MUC13	0.642879611	1.49E-57	postive
CEACAM6	MUC17	0.556787931	1.37E-40	postive
CEACAM6	MUC3B	0.546864119	6.09E-39	postive
GKN1	MUC5AC	0.74577165	1.04E-86	postive
GKN1	MUC6	0.572945263	2.13E-43	postive
IL1B	MXD1	0.593912086	2.79E-47	postive

ITGA5	MXRA7	0.613553115	3.48E-51	postive
LATS2	MXRA7	0.518752697	1.48E-34	postive
ITGA5	MXRA8	0.594173796	2.49E-47	postive
LATS2	MXRA8	0.526329128	1.07E-35	postive
ITGA5	MYADM	0.527742142	6.48E-36	postive
LATS2	MYADM	0.525672611	1.34E-35	postive
ETS1	MYCT1	0.536555181	2.76E-37	postive
ITGA5	MYCT1	0.594742894	1.93E-47	postive
ITGA5	MYL9	0.60278463	5.20E-49	postive
ITGA5	MYLK	0.548699574	3.05E-39	postive
CEACAM6	MYO1A	0.508915175	4.06E-33	postive
CD274	MYO1F	0.50403062	2.03E-32	postive
CLEC4E	MYO1F	0.635110609	8.45E-56	postive
CXCR2	MYO1F	0.58272312	3.57E-45	postive
ETS1	MYO1F	0.579169923	1.60E-44	postive
NLRC5	MYO1F	0.548476111	3.31E-39	postive
TRAF1	MYO1F	0.618242601	3.70E-52	postive
ETS1	MYO1G	0.529113752	3.99E-36	postive
NLRC5	MYO1G	0.541315746	4.82E-38	postive
TRAF1	MYO1G	0.693236716	2.78E-70	postive
ETS1	MYO5A	0.645288146	4.15E-58	postive
ITGA5	MYO5A	0.512623579	1.18E-33	postive
CEACAM6	MYO7B	0.50359564	2.34E-32	postive
ADM	NAMPT	0.648969904	5.77E-59	postive
CLEC4E	NAMPT	0.612769365	5.04E-51	postive
CXCR2	NAMPT	0.60237094	6.27E-49	postive
HIF1A	NAMPT	0.562993552	1.19E-41	postive
IL1B	NAMPT	0.657366286	5.78E-61	postive
IL17RB	NCAPG	0.510112078	2.73E-33	postive
CD274	NCF1	0.581821198	5.24E-45	postive
CLEC4E	NCF1	0.636350502	4.47E-56	postive
CXCR2	NCF1	0.53881127	1.21E-37	postive
ETS1	NCF1	0.575488302	7.45E-44	postive
NLRC5	NCF1	0.570003543	7.10E-43	postive
TRAF1	NCF1	0.578155031	2.45E-44	postive
ADM	NCF2	0.501607785	4.45E-32	postive
CD274	NCF2	0.5711806	4.39E-43	postive
CLEC4E	NCF2	0.713431752	3.71E-76	postive
CXCR2	NCF2	0.711588121	1.34E-75	postive
ETS1	NCF2	0.52056449	7.92E-35	postive
CD274	NCF4	0.504260389	1.88E-32	postive
CLEC4E	NCF4	0.577624436	3.06E-44	postive
CXCR2	NCF4	0.543376393	2.24E-38	postive

ETS1	NCF4	0.547575361	4.66E-39	postive
NLRC5	NCF4	0.521636358	5.47E-35	postive
TRAF1	NCF4	0.540452318	6.62E-38	postive
ETS1	NCKAP1L	0.619794472	1.75E-52	postive
NLRC5	NCKAP1L	0.551117594	1.21E-39	postive
TRAF1	NCKAP1L	0.559595774	4.56E-41	postive
PDIA3	NCS1	-0.501625764	4.43E-32	negative
ITGA5	NDN	0.56776736	1.76E-42	postive
IL17RB	NEK2	0.511636688	1.64E-33	postive
ARRDC3	NELFCD	-0.54350979	2.14E-38	negative
ITGA5	NEXN	0.551115502	1.22E-39	postive
ITGA5	NID1	0.590976519	1.02E-46	postive
ETS1	NID2	0.535567619	3.94E-37	postive
ITGA5	NID2	0.661231985	6.60E-62	postive
LATS2	NID2	0.545321991	1.09E-38	postive
CD274	NKG7	0.58883399	2.58E-46	postive
ETS1	NKG7	0.530659199	2.30E-36	postive
NLRC5	NKG7	0.582783529	3.48E-45	postive
ETS1	NLRC3	0.58154653	5.89E-45	postive
NLRC5	NLRC3	0.648397505	7.86E-59	postive
TRAF1	NLRC3	0.544135422	1.69E-38	postive
CLEC4E	NLRC4	0.621450682	7.81E-53	postive
CXCR2	NLRC4	0.518066079	1.87E-34	postive
ETS1	NLRC4	0.507600967	6.28E-33	postive
CD274	NLRC5	0.597565281	5.50E-48	postive
NLRC5	NLRC5	1	0	postive
TRAF1	NLRC5	0.505038566	1.46E-32	postive
CLEC4E	NLRP3	0.524335414	2.14E-35	postive
NLRC5	NMI	0.508083106	5.35E-33	postive
TRAP1	NOC4L	0.509818777	3.01E-33	postive
CLEC4E	NOD2	0.580319049	9.89E-45	postive
ITGA5	NOTCH3	0.543772203	1.94E-38	postive
PDIA3	NPIPA1	-0.506830828	8.09E-33	negative
CLEC4E	NPL	0.517186135	2.52E-34	postive
CEACAM6	NR1I2	0.534082909	6.75E-37	postive
ADAM17	NR3C2	-0.500529218	6.31E-32	negative
NR4A2	NR4A1	0.725744597	5.38E-80	postive
NR4A2	NR4A2	1	0	postive
NR4A2	NR4A3	0.675433603	1.71E-65	postive
ETS1	NRP1	0.55252992	7.07E-40	postive
ITGA5	NRP1	0.548427881	3.37E-39	postive
ITGB1	NRP1	0.568278614	1.43E-42	postive
LATS2	NRP1	0.509983727	2.85E-33	postive

ITGA5	NRP2	0.64459793	5.99E-58	postive
ITGB1	NRP2	0.550002747	1.86E-39	postive
PRTG	NRXN3	0.500512831	6.34E-32	postive
GKN1	NSG1	0.506679203	8.51E-33	postive
TRAP1	NTHL1	0.561131031	2.49E-41	postive
ITGA5	NUAK1	0.534395913	6.03E-37	postive
IL17RB	NUF2	0.503121038	2.73E-32	postive
ARRDC3	NUPR1	0.503028239	2.81E-32	postive
IL17RB	NUSAP1	0.532223233	1.32E-36	postive
ITGA5	NXN	0.530782383	2.20E-36	postive
NLRC5	OAS2	0.557215027	1.16E-40	postive
ITGA5	OCLN	-0.534761396	5.28E-37	negative
ETS1	OGFRL1	0.525011552	1.69E-35	postive
IL17RB	OIP5	0.52213282	4.61E-35	postive
ITGA5	OLFML1	0.532335441	1.26E-36	postive
ETS1	OLFML2B	0.513228696	9.62E-34	postive
ITGA5	OLFML2B	0.562153671	1.66E-41	postive
GKN1	OSBPL7	0.512370807	1.28E-33	postive
CD274	OSCAR	0.514832631	5.60E-34	postive
CLEC4E	OSCAR	0.618996695	2.57E-52	postive
ADM	OSM	0.597418714	5.87E-48	postive
CLEC4E	OSM	0.578832238	1.85E-44	postive
CXCR2	OSM	0.535000186	4.84E-37	postive
IL1B	OSM	0.690215013	1.91E-69	postive
LATS2	OSMR	0.516703284	2.97E-34	postive
ARRDC3	OSTM1	0.546276767	7.59E-39	postive
ITGA5	OSTM1	0.531002167	2.04E-36	postive
ETS1	P2RX7	0.550663805	1.44E-39	postive
NLRC5	P2RX7	0.501301411	4.92E-32	postive
ETS1	P2RY10	0.534797169	5.21E-37	postive
CLEC4E	P2RY13	0.554591116	3.20E-40	postive
CXCR2	P2RY13	0.564671906	6.10E-42	postive
ETS1	P2RY13	0.514381879	6.53E-34	postive
ETS1	P2RY8	0.564003566	7.96E-42	postive
TRAF1	P2RY8	0.556859727	1.33E-40	postive
ITGA5	P4HA3	0.545729323	9.32E-39	postive
ITGA5	PABPC4L	0.5847499	1.50E-45	postive
TRAP1	PAICS	0.583945849	2.12E-45	postive
ARRDC3	PALLD	0.508814677	4.20E-33	postive
ITGA5	PALLD	0.539565228	9.18E-38	postive
ITGA5	PAPPA	0.513576584	8.56E-34	postive
TRAP1	PAQR4	0.566288733	3.19E-42	postive
CD274	PARP9	0.525492278	1.43E-35	postive

NLRC5	PARP9	0.586545705	6.96E-46	postive
ITGA5	PARVA	0.561634745	2.04E-41	postive
ETS1	PARVG	0.547435951	4.91E-39	postive
NLRC5	PARVG	0.522723239	3.75E-35	postive
TRAF1	PARVG	0.543382819	2.24E-38	postive
ITGA5	PCAT19	0.589929519	1.60E-46	postive
TRAP1	PCBD1	0.505711592	1.17E-32	postive
ITGA5	PCDH12	0.519889677	9.99E-35	postive
ITGA5	PCDH17	0.627335135	4.30E-54	postive
LATS2	PCDH17	0.500071231	7.31E-32	postive
PDIA3	PCDH9	-0.515141421	5.05E-34	negative
PRTG	PCDH9	0.502521969	3.31E-32	postive
ITGA5	PCOLCE	0.640325549	5.69E-57	postive
LATS2	PCOLCE	0.514540705	6.18E-34	postive
CD274	PDCD1LG2	0.621121914	9.17E-53	postive
CLEC4E	PDCD1LG2	0.543577535	2.08E-38	postive
ETS1	PDCD1LG2	0.539598982	9.07E-38	postive
ITGA5	PDE3A	0.517555755	2.22E-34	postive
ARRDC3	PDE4B	0.575239413	8.26E-44	postive
CLEC4E	PDE4B	0.62541341	1.12E-53	postive
CXCR2	PDE4B	0.554507726	3.30E-40	postive
ETS1	PDE4B	0.607212307	6.79E-50	postive
IL1B	PDE4B	0.500890488	5.62E-32	postive
TRAF1	PDE4B	0.501619555	4.44E-32	postive
ARRDC3	PDGFC	0.507665697	6.15E-33	postive
ITGB1	PDGFC	0.501437988	4.71E-32	postive
ITGA5	PDGFRB	0.611144902	1.08E-50	postive
LATS2	PDGFRB	0.544715628	1.36E-38	postive
PDIA3	PDIA4	0.620889655	1.03E-52	postive
ITGA5	PDLIM3	0.568431679	1.34E-42	postive
ITGA5	PDLIM4	0.626442572	6.70E-54	postive
ITGA5	PDLIM7	0.609637559	2.20E-50	postive
ITGA5	PDPN	0.57404126	1.36E-43	postive
PDIA3	PDZRN4	-0.506977927	7.71E-33	negative
ITGA5	PEAR1	0.569917664	7.35E-43	postive
ETS1	PECAM1	0.644827965	5.30E-58	postive
ITGA5	PECAM1	0.570537107	5.71E-43	postive
CEACAM6	PERP	0.511621432	1.65E-33	postive
ADM	PFKFB3	0.569887487	7.44E-43	postive
HIF1A	PFKFB3	0.564481068	6.58E-42	postive
IL1B	PFKFB3	0.528683112	4.64E-36	postive
TLR5	PGAM5	-0.502051186	3.86E-32	negative
TRAP1	PGAM5	0.507242354	7.07E-33	postive

GKN1	PGC	0.756018064	2.16E-90	postive
CLEC4E	PHACTR1	0.5198343	1.02E-34	postive
TRAF1	PHACTR1	0.522067348	4.71E-35	postive
ITGA5	PHLDB2	0.505737224	1.16E-32	postive
HIF1A	PI15	0.54003209	7.73E-38	postive
ITGA5	PI15	0.54221431	3.46E-38	postive
ETS1	PIK3CD	0.595284401	1.52E-47	postive
NLRC5	PIK3CD	0.523430646	2.93E-35	postive
TRAF1	PIK3CD	0.612626655	5.39E-51	postive
ETS1	PIK3CG	0.585646661	1.02E-45	postive
CD274	PILRA	0.591423761	8.35E-47	postive
CLEC4E	PILRA	0.760363462	5.21E-92	postive
CXCR2	PILRA	0.607028546	7.39E-50	postive
ETS1	PILRA	0.562411697	1.50E-41	postive
MCOLN1	PILRA	0.50735253	6.82E-33	postive
ARRDC3	PJA2	0.652923478	6.73E-60	postive
ARRDC3	PKD2	0.507122529	7.35E-33	postive
ITGA5	PKD2	0.548983337	2.73E-39	postive
ITGB1	PKD2	0.501441748	4.70E-32	postive
LATS2	PKD2	0.508790438	4.24E-33	postive
ITGA5	PKIG	0.529044148	4.09E-36	postive
TRAP1	PKMYT1	0.582463795	3.99E-45	postive
ITGA5	PLA2G4C	0.536188313	3.15E-37	postive
CD274	PLA2G7	0.565703915	4.03E-42	postive
CLEC4E	PLA2G7	0.516133497	3.61E-34	postive
HPSE	PLA2G7	0.511752814	1.58E-33	postive
ADM	PLAU	0.630325276	9.61E-55	postive
HIF1A	PLAU	0.526195096	1.12E-35	postive
IL1B	PLAU	0.545299555	1.10E-38	postive
MMP10	PLAU	0.541343495	4.77E-38	postive
ADM	PLAUR	0.57523944	8.26E-44	postive
CD274	PLAUR	0.500453604	6.47E-32	postive
IL1B	PLAUR	0.675768305	1.40E-65	postive
CD274	PLEK	0.683315882	1.43E-67	postive
CLEC4E	PLEK	0.752776899	3.31E-89	postive
CXCR2	PLEK	0.61542357	1.43E-51	postive
ETS1	PLEK	0.56570753	4.03E-42	postive
IL1B	PLEK	0.569136409	1.01E-42	postive
TRAF1	PLEK	0.579091927	1.66E-44	postive
ETS1	PLEKHO1	0.514729013	5.80E-34	postive
ITGA5	PLEKHO1	0.655893331	1.31E-60	postive
ETS1	PLEKHO2	0.567850328	1.70E-42	postive
ITGA5	PLEKHO2	0.571410582	4.00E-43	postive

TRAF1	PLEKHO2	0.533968872	7.03E-37	postive
ITGA5	PLS1	-0.502097686	3.80E-32	negative
ITGA5	PLVAP	0.52312173	3.27E-35	postive
ETS1	PLXNC1	0.564489842	6.56E-42	postive
CD274	PMAIP1	0.53343063	8.53E-37	postive
ITGA5	PMP22	0.507982125	5.54E-33	postive
ARRDC3	PNRC1	0.532490897	1.20E-36	postive
CEACAM1	POF1B	0.501778085	4.22E-32	postive
CEACAM6	POF1B	0.524183952	2.26E-35	postive
TRAP1	POLD2	0.501789864	4.20E-32	postive
IL17RB	POLE2	0.503619918	2.32E-32	postive
ITGA5	POPDC2	0.532562843	1.17E-36	postive
PDIA3	POU6F1	-0.548543084	3.23E-39	negative
TRAP1	PPAT	0.530421645	2.50E-36	postive
ADM	PPBP	0.535367766	4.24E-37	postive
IL1B	PPIF	0.548955123	2.76E-39	postive
CEACAM6	PPP1R14D	0.526894464	8.73E-36	postive
ETS1	PPP1R16B	0.570005823	7.09E-43	postive
NLRC5	PPP1R16B	0.520827265	7.23E-35	postive
TRAF1	PPP1R16B	0.540415385	6.71E-38	postive
ETS1	PPP1R18	0.56751492	1.95E-42	postive
ITGA5	PPP1R18	0.616548807	8.34E-52	postive
TRAF1	PPP1R18	0.53520534	4.50E-37	postive
IL17RB	PRC1	0.512195572	1.36E-33	postive
CD274	PRDM1	0.560346625	3.39E-41	postive
TRAF1	PRDM1	0.504615089	1.67E-32	postive
CLEC4E	PREX1	0.5247709	1.84E-35	postive
ETS1	PREX1	0.629053942	1.82E-54	postive
TRAF1	PREX1	0.59077763	1.11E-46	postive
ITGA5	PREX2	0.520708253	7.54E-35	postive
CD274	PRF1	0.662040529	4.17E-62	postive
NLRC5	PRF1	0.624800301	1.51E-53	postive
TRAF1	PRF1	0.508670669	4.41E-33	postive
ITGA5	PRICKLE2	0.615588732	1.32E-51	postive
ITGA5	PRKCDBP	0.596928258	7.31E-48	postive
ETS1	PRKCH	0.658658854	2.81E-61	postive
TRAF1	PRKCH	0.503148414	2.70E-32	postive
ARRDC3	PRKCZ	-0.514667936	5.92E-34	negative
ITGA5	PRKD1	0.542518741	3.09E-38	postive
ITGA5	PRKG1	0.543154882	2.44E-38	postive
ITGB1	PRNP	0.517432995	2.32E-34	postive
ADM	PROK2	0.586647918	6.66E-46	postive
CLEC4E	PROK2	0.614682164	2.03E-51	postive

CXCR2	PROK2	0.658664673	2.80E-61	postive
IL1B	PROK2	0.704234012	2.03E-73	postive
CEACAM6	PRR15	0.587940608	3.81E-46	postive
CEACAM6	PRR15L	0.515325348	4.74E-34	postive
ETS1	PRR16	0.509494362	3.35E-33	postive
ITGA5	PRR16	0.586279001	7.80E-46	postive
ITGA5	PRR24	0.557932132	8.74E-41	postive
ARRDC3	PRRX1	0.505504842	1.25E-32	postive
ETS1	PRRX1	0.527925541	6.07E-36	postive
ITGA5	PRRX1	0.612260039	6.41E-51	postive
LATS2	PRRX1	0.508705203	4.36E-33	postive
ITGA5	PRSS23	0.600670805	1.36E-48	postive
CEACAM1	PRSS3	0.523883517	2.51E-35	postive
CEACAM6	PRSS3	0.56955441	8.52E-43	postive
PRTG	PRTG	1	0	postive
GKN1	PSAPL1	0.617879326	4.40E-52	postive
GKN1	PSCA	0.685663163	3.34E-68	postive
CD274	PSMB10	0.504265974	1.88E-32	postive
NLRC5	PSMB10	0.656341275	1.02E-60	postive
NLRC5	PSMB8	0.580452886	9.34E-45	postive
NLRC5	PSMB8-AS1	0.648814977	6.28E-59	postive
CD274	PSMB9	0.599796718	2.02E-48	postive
NLRC5	PSMB9	0.7110114	2.00E-75	postive
ETS1	PSTPIP1	0.512771178	1.12E-33	postive
TRAF1	PSTPIP1	0.529190173	3.88E-36	postive
CD274	PTAFR	0.521913373	4.97E-35	postive
CLEC4E	PTAFR	0.606676768	8.70E-50	postive
CXCR2	PTAFR	0.561426448	2.21E-41	postive
ETS1	PTAFR	0.525489197	1.43E-35	postive
ITGA5	PTGFR	0.526945446	8.58E-36	postive
ADM	PTGS2	0.56565131	4.12E-42	postive
IL1B	PTGS2	0.52527382	1.54E-35	postive
CEACAM6	PTK6	0.509910734	2.92E-33	postive
TLR5	PTPLAD2	0.506479009	9.09E-33	postive
LATS2	PTPN14	0.562205518	1.63E-41	postive
CD274	PTPN22	0.591851405	6.92E-47	postive
ETS1	PTPN22	0.60018067	1.69E-48	postive
NLRC5	PTPN22	0.682264916	2.73E-67	postive
TRAF1	PTPN22	0.539905779	8.10E-38	postive
CD274	PTPRC	0.590085165	1.50E-46	postive
CLEC4E	PTPRC	0.576649633	4.60E-44	postive
ETS1	PTPRC	0.699057685	6.33E-72	postive
NLRC5	PTPRC	0.597873223	4.79E-48	postive

TRAF1	PTPRC	0.567352935	2.08E-42	postive
NLRC5	PTPRCAP	0.562879231	1.25E-41	postive
TRAF1	PTPRCAP	0.517960824	1.93E-34	postive
ITGA5	PTPRM	0.604042021	2.92E-49	postive
LATS2	PTPRM	0.543220781	2.38E-38	postive
ITGA5	PTRF	0.696494541	3.39E-71	postive
ITGB1	PTRF	0.547534809	4.73E-39	postive
LATS2	PTRF	0.583369483	2.71E-45	postive
TRAP1	PUS1	0.515915631	3.88E-34	postive
TRAP1	PUS7	0.509507818	3.34E-33	postive
ETS1	PVRIG	0.554321918	3.55E-40	postive
NLRC5	PVRIG	0.554363723	3.49E-40	postive
TRAF1	PVRIG	0.533844477	7.35E-37	postive
ITGA5	PXDN	0.756089072	2.03E-90	postive
LATS2	PXDN	0.544900929	1.27E-38	postive
ITGA5	PXMP2	-0.524713451	1.88E-35	negative
ETS1	PYHIN1	0.547397889	4.98E-39	postive
NLRC5	PYHIN1	0.587777743	4.09E-46	postive
TRAF1	PYHIN1	0.555467532	2.28E-40	postive
ARRDC3	QKI	0.520103902	9.28E-35	postive
ETS1	QKI	0.565401529	4.55E-42	postive
ITGA5	QKI	0.615719314	1.24E-51	postive
LATS2	QKI	0.548500052	3.28E-39	postive
ARRDC3	RAB11FIP4	-0.522916061	3.51E-35	negative
ITGA5	RAB23	0.56278483	1.29E-41	postive
ITGB1	RAB23	0.575392853	7.76E-44	postive
CEACAM6	RAB25	0.502552275	3.28E-32	postive
GKN1	RAB27A	0.534250242	6.35E-37	postive
ITGA5	RAB31	0.706656917	3.95E-74	postive
ITGB1	RAB31	0.517753619	2.08E-34	postive
LATS2	RAB31	0.570725795	5.29E-43	postive
ETS1	RAB33A	0.504338024	1.83E-32	postive
ARRDC3	RAB8B	0.537380092	2.04E-37	postive
ETS1	RAB8B	0.63684045	3.47E-56	postive
CD274	RAC2	0.573513852	1.69E-43	postive
CLEC4E	RAC2	0.531755835	1.56E-36	postive
ETS1	RAC2	0.603749469	3.34E-49	postive
NLRC5	RAC2	0.543294508	2.31E-38	postive
TRAF1	RAC2	0.570158692	6.66E-43	postive
IL17RB	RAD51AP1	0.530138132	2.77E-36	postive
ITGA5	RAI14	0.636837297	3.48E-56	postive
ITGA5	RARRES2	0.576854463	4.22E-44	postive
LATS2	RARRES2	0.514284726	6.74E-34	postive

CD274	RARRES3	0.524546824	1.99E-35	postive
NLRC5	RARRES3	0.599491283	2.31E-48	postive
NLRC5	RASAL3	0.539051886	1.11E-37	postive
TRAF1	RASAL3	0.562482725	1.46E-41	postive
ITGA5	RASGRF2	0.673378157	5.82E-65	postive
LATS2	RASGRF2	0.505337581	1.32E-32	postive
NLRC5	RASGRP1	0.527705376	6.56E-36	postive
TRAF1	RASGRP1	0.520054446	9.44E-35	postive
ETS1	RASGRP3	0.590238114	1.40E-46	postive
ITGA5	RASIP1	0.539746176	8.59E-38	postive
ITGA5	RASL12	0.566236194	3.26E-42	postive
ETS1	RASSF2	0.613719031	3.21E-51	postive
CLEC4E	RASSF5	0.507731673	6.01E-33	postive
ETS1	RASSF5	0.53580265	3.62E-37	postive
NLRC5	RASSF5	0.526072184	1.17E-35	postive
TRAF1	RASSF5	0.536422793	2.89E-37	postive
ITGA5	RASSF8	0.603378227	3.96E-49	postive
ITGA5	RASSF8-AS1	0.569846031	7.57E-43	postive
ITGA5	RBM47	-0.520450968	8.23E-35	negative
PDIA3	RBM47	0.517874088	1.99E-34	postive
ITGA5	RBMS1	0.521918245	4.96E-35	postive
LATS2	RBMS1	0.56191741	1.82E-41	postive
ITGA5	RBPMS	0.527819836	6.30E-36	postive
LATS2	RBPMS	0.526757849	9.16E-36	postive
PRTG	RBPMS2	0.530903945	2.11E-36	postive
ARRDC3	RCBTB2	0.516390455	3.30E-34	postive
TRAP1	RCC1	0.516877463	2.80E-34	postive
ETS1	RCSD1	0.612408958	5.97E-51	postive
TRAF1	RCSD1	0.53176968	1.55E-36	postive
GKN1	RDH12	0.545376243	1.06E-38	postive
ETS1	RECQL	0.501682949	4.35E-32	postive
CEACAM6	REG4	0.564949162	5.46E-42	postive
ETS1	RFTN1	0.595814094	1.20E-47	postive
TRAF1	RFTN1	0.5103144	2.55E-33	postive
CLEC4E	RGL4	0.524211912	2.24E-35	postive
TRAF1	RGL4	0.5189481	1.38E-34	postive
ARRDC3	RGS18	0.506029242	1.05E-32	postive
CLEC4E	RGS18	0.515654219	4.24E-34	postive
CXCR2	RGS18	0.533801719	7.47E-37	postive
ETS1	RGS18	0.598087967	4.35E-48	postive
ITGA5	RHOB	0.513867257	7.76E-34	postive
CTTN	RHOD	0.525302671	1.53E-35	postive
CD274	RHOH	0.544296216	1.59E-38	postive

CLEC4E	RHOH	0.546497473	6.99E-39	postive
ETS1	RHOH	0.599348795	2.47E-48	postive
NLRC5	RHOH	0.537488926	1.96E-37	postive
TRAF1	RHOH	0.652923495	6.73E-60	postive
ITGA5	RHOJ	0.654743642	2.47E-60	postive
LATS2	RHOJ	0.520515844	8.05E-35	postive
ARRDC3	RHOQ	0.505694056	1.18E-32	postive
ITGA5	RHOQ	0.664691616	9.21E-63	postive
ITGB1	RHOQ	0.521134092	6.51E-35	postive
LATS2	RHOQ	0.575863319	6.38E-44	postive
CEACAM1	RHPN2	0.513113333	1.00E-33	postive
PDIA3	RHPN2	0.542012473	3.72E-38	postive
ITGA5	RILPL1	0.543282315	2.32E-38	postive
LATS2	RILPL1	0.524720182	1.87E-35	postive
CLEC4E	RILPL2	0.603362905	3.99E-49	postive
ETS1	RILPL2	0.54900816	2.71E-39	postive
TRAF1	RILPL2	0.533138335	9.48E-37	postive
CD274	RIPK2	0.500759717	5.86E-32	postive
GKN1	RNASE1	0.535753112	3.69E-37	postive
ETS1	RNASE6	0.521634447	5.47E-35	postive
PDIA3	RNF150	-0.546034772	8.31E-39	negative
CEACAM6	RNF186	0.572838414	2.23E-43	postive
CD274	RNF19B	0.586679359	6.57E-46	postive
HPSE	RNF19B	0.532686968	1.11E-36	postive
CD274	RNF213	0.548759952	2.98E-39	postive
NLRC5	RNF213	0.615076785	1.69E-51	postive
ITGA5	RNF217	0.543243687	2.36E-38	postive
ITGA5	ROBO1	0.525173223	1.60E-35	postive
ETS1	ROBO4	0.516278533	3.43E-34	postive
HIF1A	ROBO4	0.514272256	6.77E-34	postive
ITGA5	ROBO4	0.61111914	1.10E-50	postive
ITGA5	ROR2	0.536762232	2.56E-37	postive
ITGA5	RP1-142L7.8	0.51810944	1.84E-34	postive
CD274	RP1-93H18.6	0.556937555	1.29E-40	postive
NLRC5	RP1-93H18.6	0.594007236	2.68E-47	postive
TRAF1	RP1-93H18.6	0.501737434	4.27E-32	postive
GKN1	RP11-363E7.4	0.651628984	1.36E-59	postive
ETS1	RP11-389C8.2	0.53725209	2.14E-37	postive
ADM	RP11-548H18.2	0.513001505	1.04E-33	postive
CLEC4E	RP11-548H18.2	0.547142008	5.48E-39	postive
IL1B	RP11-548H18.2	0.615353846	1.48E-51	postive
PDIA3	RP11-642D21.1	-0.505292073	1.34E-32	negative
ITGB1	RRAGB	0.519586535	1.11E-34	postive

TRAP1	RRP9	0.545298391	1.10E-38	postive
NLRC5	RTP4	0.502071061	3.83E-32	postive
PDIA3	RUNX1T1	-0.539889614	8.15E-38	negative
TRAF1	RUNX3	0.501902816	4.05E-32	postive
ITGA5	RUSC2	0.507880944	5.72E-33	postive
PRTG	RYR3	0.504681661	1.64E-32	postive
ADM	S100A12	0.551254837	1.15E-39	postive
CLEC4E	S100A12	0.652504618	8.46E-60	postive
CXCR2	S100A12	0.705313279	9.80E-74	postive
HIF1A	S100A12	0.511082076	1.98E-33	postive
IL1B	S100A12	0.605742346	1.34E-49	postive
CEACAM6	S100A14	0.518176255	1.80E-34	postive
ADM	S100A8	0.58356066	2.50E-45	postive
CLEC4E	S100A8	0.675683709	1.48E-65	postive
CXCR2	S100A8	0.783699185	2.50E-101	postive
HIF1A	S100A8	0.53348708	8.36E-37	postive
IL1B	S100A8	0.622094807	5.70E-53	postive
ADM	S100A9	0.582602123	3.76E-45	postive
CLEC4E	S100A9	0.59314343	3.92E-47	postive
CXCR2	S100A9	0.659307325	1.95E-61	postive
IL1B	S100A9	0.581710082	5.49E-45	postive
ETS1	S1PR1	0.563037861	1.17E-41	postive
ITGA5	S1PR1	0.521669542	5.41E-35	postive
ITGA5	S1PR3	0.540717048	6.01E-38	postive
PDIA3	SALL2	-0.521060957	6.67E-35	negative
PRTG	SALL2	0.550795112	1.37E-39	postive
NLRC5	SAMD3	0.557347995	1.10E-40	postive
ITGA5	SAMD4A	0.654505806	2.82E-60	postive
LATS2	SAMD4A	0.508578012	4.55E-33	postive
CD274	SAMD9L	0.614848909	1.88E-51	postive
NLRC5	SAMD9L	0.673994446	4.04E-65	postive
ETS1	SAMHD1	0.546225951	7.74E-39	postive
NLRC5	SAMHD1	0.533563048	8.14E-37	postive
CD274	SAMSN1	0.621083803	9.34E-53	postive
CLEC4E	SAMSN1	0.679236504	1.73E-66	postive
CXCR2	SAMSN1	0.583242668	2.86E-45	postive
ETS1	SAMSN1	0.682392068	2.53E-67	postive
TRAF1	SAMSN1	0.587745279	4.14E-46	postive
ITGA5	SASH1	0.506698461	8.45E-33	postive
CD274	SASH3	0.503784905	2.20E-32	postive
ETS1	SASH3	0.554869025	2.87E-40	postive
NLRC5	SASH3	0.624452115	1.79E-53	postive
TRAF1	SASH3	0.645346534	4.03E-58	postive

IL1B	SAT1	0.52828476	5.35E-36	postive
GKN1	SCGB2A1	0.713900696	2.67E-76	postive
GKN1	SCNN1B	0.513310085	9.36E-34	postive
ARRDC3	SDC2	0.515406292	4.61E-34	postive
ITGA5	SDC2	0.512724263	1.14E-33	postive
ITGB1	SDC2	0.548810109	2.92E-39	postive
PDIA3	SDF2L1	0.561888739	1.84E-41	postive
ITGB1	SEC23A	0.633389551	2.04E-55	postive
PDIA3	SEC23B	0.519559591	1.12E-34	postive
CLEC4E	SELL	0.624170979	2.06E-53	postive
CXCR2	SELL	0.649963666	3.37E-59	postive
ETS1	SELL	0.641369706	3.29E-57	postive
TRAF1	SELL	0.595725809	1.25E-47	postive
ITGA5	SELM	0.592778794	4.60E-47	postive
LATS2	SELM	0.512800801	1.11E-33	postive
ETS1	SELPLG	0.505400402	1.29E-32	postive
NLRC5	SELPLG	0.549105931	2.61E-39	postive
TRAF1	SELPLG	0.56980252	7.70E-43	postive
ITGA5	SEMA4C	0.5464053	7.23E-39	postive
TRAP1	SEPP1	-0.503906805	2.11E-32	negative
ITGB1	10-Sep	0.60067501	1.36E-48	postive
CEACAM6	SERPINB5	0.566980541	2.41E-42	postive
CD274	SERPINB9	0.50987653	2.95E-33	postive
CLEC4E	SERPINB9	0.518343067	1.70E-34	postive
TRAF1	SERPINB9	0.540807072	5.81E-38	postive
ADM	SERPINE1	0.579975273	1.14E-44	postive
ETS1	SERPINE1	0.519378323	1.19E-34	postive
HIF1A	SERPINE1	0.540196963	7.28E-38	postive
ITGA5	SERPINE1	0.510303915	2.56E-33	postive
ITGA5	SERPINF1	0.519002616	1.35E-34	postive
ETS1	SERPING1	0.508014467	5.48E-33	postive
ITGA5	SERPING1	0.513628332	8.41E-34	postive
ITGA5	SERPINH1	0.577687498	2.98E-44	postive
LATS2	SERPINH1	0.500406388	6.57E-32	postive
ETS1	SFMBT2	0.584093865	1.99E-45	postive
NLRC5	SFMBT2	0.564584827	6.31E-42	postive
TRAF1	SFMBT2	0.584238944	1.87E-45	postive
GKN1	SGSM3	0.514051668	7.29E-34	postive
ETS1	SGTB	0.521175418	6.41E-35	postive
ITGA5	SGTB	0.505026765	1.46E-32	postive
ETS1	SH2B3	0.691473696	8.59E-70	postive
CD274	SH2D1A	0.52868237	4.65E-36	postive
ETS1	SH2D1A	0.543241431	2.36E-38	postive

NLRC5	SH2D1A	0.54889	2.83E-39	postive
ITGA5	SH2D3C	0.506084639	1.03E-32	postive
ARRDC3	SH3BGRL	0.527485795	7.09E-36	postive
ETS1	SH3BP5	0.581745824	5.41E-45	postive
ITGA5	SH3BP5	0.50351787	2.40E-32	postive
ITGA5	SH3PXD2B	0.603291542	4.12E-49	postive
ITGA5	SH3RF3	0.632389957	3.38E-55	postive
ITGA5	SHANK3	0.574036062	1.36E-43	postive
CD274	SIGLEC10	0.594524355	2.13E-47	postive
CLEC4E	SIGLEC10	0.554096432	3.87E-40	postive
ETS1	SIGLEC10	0.50866776	4.41E-33	postive
NLRC5	SIGLEC10	0.532616974	1.14E-36	postive
CLEC4E	SIGLEC5	0.650706384	2.25E-59	postive
CXCR2	SIGLEC5	0.575023351	9.04E-44	postive
CLEC4E	SIGLEC9	0.524130085	2.30E-35	postive
NR4A2	SIK1	0.699511363	4.70E-72	postive
CD274	SIRPG	0.521883719	5.02E-35	postive
NLRC5	SIRPG	0.560624198	3.04E-41	postive
TRAF1	SIRPG	0.512968843	1.05E-33	postive
TRAF1	SIT1	0.529916837	3.00E-36	postive
PRTG	SKIDA1	0.525019486	1.69E-35	postive
CD274	SLA	0.596570708	8.57E-48	postive
CLEC4E	SLA	0.630792293	7.59E-55	postive
CXCR2	SLA	0.523741572	2.63E-35	postive
ETS1	SLA	0.713553872	3.41E-76	postive
NLRC5	SLA	0.593147839	3.91E-47	postive
TRAF1	SLA	0.621265684	8.55E-53	postive
CD274	SLAMF1	0.553043199	5.81E-40	postive
CLEC4E	SLAMF1	0.522894403	3.54E-35	postive
ETS1	SLAMF1	0.525040662	1.67E-35	postive
NLRC5	SLAMF1	0.520400179	8.38E-35	postive
TRAF1	SLAMF1	0.712251152	8.45E-76	postive
NLRC5	SLAMF6	0.512628407	1.18E-33	postive
TRAF1	SLAMF6	0.542367628	3.26E-38	postive
CD274	SLAMF7	0.575489569	7.45E-44	postive
ETS1	SLAMF7	0.50860878	4.50E-33	postive
NLRC5	SLAMF7	0.538740465	1.24E-37	postive
CD274	SLAMF8	0.659295036	1.96E-61	postive
CLEC4E	SLAMF8	0.62314207	3.41E-53	postive
ETS1	SLAMF8	0.595086702	1.66E-47	postive
NLRC5	SLAMF8	0.562526216	1.43E-41	postive
TRAF1	SLAMF8	0.540244897	7.15E-38	postive
CD274	SLC15A3	0.518303328	1.72E-34	postive

MCOLN1	SLC15A3	0.502225604	3.65E-32	postive
NLRC5	SLC15A3	0.527361482	7.41E-36	postive
CD274	SLC16A6	0.537765048	1.77E-37	postive
CLEC4E	SLC16A6	0.556825275	1.35E-40	postive
CEACAM6	SLC22A18	0.510223374	2.63E-33	postive
TRAP1	SLC25A10	0.561859091	1.87E-41	postive
PDIA3	SLC25A27	-0.505259963	1.36E-32	negative
ADM	SLC2A3	0.619288708	2.23E-52	postive
CLEC4E	SLC2A3	0.601647262	8.72E-49	postive
ETS1	SLC2A3	0.535981389	3.40E-37	postive
ITGA5	SLC2A3	0.507852215	5.78E-33	postive
PDIA3	SLC31A1	0.519430895	1.17E-34	postive
CD274	SLC31A2	0.592296584	5.69E-47	postive
CLEC4E	SLC31A2	0.550213374	1.71E-39	postive
ETS1	SLC31A2	0.510549917	2.36E-33	postive
ARRDC3	SLC5A6	-0.505833366	1.12E-32	negative
CEACAM6	SLC6A20	0.519666602	1.08E-34	postive
GKN1	SLC7A8	0.540583008	6.31E-38	postive
ETS1	SLFN5	0.558578921	6.79E-41	postive
PDIA3	SLITRK5	-0.522564667	3.97E-35	negative
ITGB1	SLMAP	0.520655732	7.67E-35	postive
TRAF1	SMAP2	0.503448581	2.45E-32	postive
GKN1	SMIM5	0.542182442	3.50E-38	postive
GKN1	SMIM6	0.520853759	7.17E-35	postive
ITGA5	SMTN	0.511380924	1.79E-33	postive
PRTG	SMYD1	0.503678847	2.27E-32	postive
ETS1	SNAI2	0.517551864	2.22E-34	postive
ITGB1	SNAI2	0.503242199	2.62E-32	postive
HIF1A	SNAPC1	0.523779293	2.60E-35	postive
TLR5	SNRPB	-0.516239386	3.48E-34	negative
CD274	SNX10	0.597088122	6.80E-48	postive
CLEC4E	SNX10	0.58247372	3.97E-45	postive
CD274	SNX20	0.574530281	1.11E-43	postive
CLEC4E	SNX20	0.550909398	1.31E-39	postive
ETS1	SNX20	0.562255562	1.59E-41	postive
NLRC5	SNX20	0.608238542	4.22E-50	postive
TRAF1	SNX20	0.634934829	9.25E-56	postive
ADM	SOCS3	0.503733208	2.23E-32	postive
HIF1A	SOCS3	0.513411435	9.05E-34	postive
IL1B	SOCS3	0.521927636	4.94E-35	postive
LATS2	SOCS3	0.505773018	1.15E-32	postive
HIF1A	SOD2	0.540493967	6.52E-38	postive
GKN1	SOSTDC1	0.738566976	3.19E-84	postive

ITGA5	SOX17	0.585833673	9.45E-46	postive
ITGA5	SOX18	0.500952557	5.51E-32	postive
IL17RB	SOX9	0.539126756	1.08E-37	postive
CD274	SP110	0.556112907	1.78E-40	postive
NLRC5	SP110	0.655591996	1.55E-60	postive
ITGA5	SPARC	0.62610799	7.91E-54	postive
LATS2	SPARC	0.517545193	2.23E-34	postive
ITGA5	SPARCL1	0.517656618	2.15E-34	postive
PDIA3	SPINT1	0.535746478	3.70E-37	postive
CEACAM1	SPINT2	0.546041918	8.29E-39	postive
PDIA3	SPINT2	0.525369805	1.49E-35	postive
ITGA5	SPOCK1	0.557201895	1.16E-40	postive
ITGB1	SPOCK1	0.502692832	3.13E-32	postive
ADM	SPP1	0.511598883	1.66E-33	postive
ARRDC3	SRGN	0.509919297	2.91E-33	postive
CD274	SRGN	0.565955608	3.64E-42	postive
CLEC4E	SRGN	0.697004948	2.43E-71	postive
CXCR2	SRGN	0.65208186	1.07E-59	postive
ETS1	SRGN	0.678319964	3.02E-66	postive
ITGA5	SRPX2	0.540533395	6.43E-38	postive
ARRDC3	SSBP2	0.525848646	1.26E-35	postive
GKN1	SST	0.632021481	4.08E-55	postive
GKN1	SSTR1	0.563611031	9.31E-42	postive
ITGA5	ST6GALNAC5	0.533978549	7.00E-37	postive
CD274	ST8SIA4	0.554296983	3.58E-40	postive
CLEC4E	ST8SIA4	0.568524076	1.29E-42	postive
CXCR2	ST8SIA4	0.518466589	1.63E-34	postive
ETS1	ST8SIA4	0.756492167	1.44E-90	postive
NLRC5	ST8SIA4	0.541720805	4.15E-38	postive
TRAF1	ST8SIA4	0.528821746	4.42E-36	postive
PDIA3	STARD9	-0.515305087	4.78E-34	negative
CD274	STAT1	0.625847614	9.00E-54	postive
NLRC5	STAT1	0.527824018	6.29E-36	postive
CD274	STAT4	0.535393398	4.20E-37	postive
CLEC4E	STAT4	0.514365188	6.56E-34	postive
ETS1	STAT4	0.606433744	9.73E-50	postive
NLRC5	STAT4	0.569816126	7.66E-43	postive
TRAF1	STAT4	0.629164764	1.72E-54	postive
CD274	STK17B	0.516633276	3.04E-34	postive
TRAF1	STK17B	0.500839258	5.71E-32	postive
ITGA5	STOM	0.53381371	7.43E-37	postive
LATS2	STOM	0.555308572	2.42E-40	postive
ITGA5	STON1	0.612208908	6.57E-51	postive

LATS2	STON1	0.508042183	5.43E-33	postive
CD274	STX11	0.63934097	9.50E-57	postive
CLEC4E	STX11	0.678933932	2.08E-66	postive
CXCR2	STX11	0.541524547	4.46E-38	postive
ETS1	STX11	0.655042198	2.10E-60	postive
NLRC5	STX11	0.519115246	1.30E-34	postive
TRAF1	STX11	0.543963548	1.80E-38	postive
GKN1	SULT1B1	0.531567175	1.66E-36	postive
GKN1	SULT1C2	0.612061665	7.04E-51	postive
ITGA5	SYNC	0.563591546	9.38E-42	postive
ITGB1	SYNC	0.528367786	5.19E-36	postive
PDIA3	SYNE1	-0.501802088	4.18E-32	negative
ITGA5	SYT11	0.540622073	6.22E-38	postive
GKN1	SYTL2	0.515831465	4.00E-34	postive
GKN1	SYTL5	0.605989729	1.19E-49	postive
CD274	TAGAP	0.597591366	5.43E-48	postive
CLEC4E	TAGAP	0.659715901	1.55E-61	postive
CXCR2	TAGAP	0.533790311	7.50E-37	postive
ETS1	TAGAP	0.615449622	1.41E-51	postive
NLRC5	TAGAP	0.516249806	3.47E-34	postive
TRAF1	TAGAP	0.648474556	7.54E-59	postive
ITGA5	TAGLN	0.592436612	5.35E-47	postive
CD274	TAP1	0.624970293	1.39E-53	postive
NLRC5	TAP1	0.665468376	5.90E-63	postive
CD274	TAP2	0.64839811	7.86E-59	postive
NLRC5	TAP2	0.622237052	5.32E-53	postive
NLRC5	TBC1D10C	0.600620532	1.39E-48	postive
TRAF1	TBC1D10C	0.561718546	1.97E-41	postive
TRAP1	TBC1D9	-0.505492571	1.26E-32	negative
CD274	TBX21	0.557049858	1.23E-40	postive
NLRC5	TBX21	0.593018198	4.14E-47	postive
TRAF1	TBX21	0.510637292	2.29E-33	postive
ARRDC3	TCEAL7	0.516044104	3.72E-34	postive
ITGA5	TCEAL7	0.540294544	7.02E-38	postive
ETS1	TCF4	0.526301208	1.08E-35	postive
ITGA5	TCF4	0.58947897	1.95E-46	postive
LATS2	TCF4	0.50347395	2.43E-32	postive
ITGA5	TENC1	0.500561811	6.25E-32	postive
TRAF1	TESPA1	0.521937735	4.93E-35	postive
CD274	TFEC	0.539489398	9.44E-38	postive
ETS1	TFEC	0.533210605	9.23E-37	postive
GKN1	TFF1	0.669443179	5.90E-64	postive
GKN1	TFF2	0.758961904	1.75E-91	postive

ITGA5	TGFB1I1	0.676188881	1.09E-65	postive
LATS2	TGFB1I1	0.524529054	2.00E-35	postive
ITGA5	TGFB3	0.563610313	9.31E-42	postive
ITGA5	TGFBI	0.613944571	2.89E-51	postive
ITGA5	THBD	0.59172029	7.33E-47	postive
LATS2	THBD	0.554811807	2.94E-40	postive
ETS1	THBS1	0.512899519	1.08E-33	postive
ITGA5	THBS1	0.655645067	1.50E-60	postive
LATS2	THBS1	0.596565887	8.59E-48	postive
PDIA3	THBS3	-0.529731021	3.20E-36	negative
ETS1	THEMIS	0.551748741	9.54E-40	postive
NLRC5	THEMIS	0.567512602	1.95E-42	postive
CD274	THEMIS2	0.541307352	4.83E-38	postive
CLEC4E	THEMIS2	0.602287826	6.52E-49	postive
CXCR2	THEMIS2	0.51928372	1.23E-34	postive
ETS1	THEMIS2	0.668131112	1.27E-63	postive
TRAF1	THEMIS2	0.534632169	5.53E-37	postive
ITGA5	THY1	0.674163403	3.65E-65	postive
LATS2	THY1	0.539689378	8.77E-38	postive
ITGA5	TIE1	0.645035486	4.75E-58	postive
CD274	TIFA	0.515692311	4.19E-34	postive
CD274	TIGIT	0.571237904	4.29E-43	postive
ETS1	TIGIT	0.542786901	2.79E-38	postive
NLRC5	TIGIT	0.620955482	9.94E-53	postive
ITGA5	TIMP1	0.521363494	6.01E-35	postive
ITGA5	TIMP2	0.612197359	6.60E-51	postive
LATS2	TIMP2	0.612682528	5.25E-51	postive
ARRDC3	TLR1	0.55036317	1.62E-39	postive
CD274	TLR1	0.505914083	1.09E-32	postive
CLEC4E	TLR1	0.540506452	6.49E-38	postive
ETS1	TLR1	0.602140546	6.97E-49	postive
ADM	TLR2	0.534889	5.04E-37	postive
CLEC4E	TLR2	0.625380642	1.13E-53	postive
CXCR2	TLR2	0.534774911	5.26E-37	postive
ARRDC3	TLR4	0.541461995	4.56E-38	postive
CLEC4E	TLR4	0.59570631	1.26E-47	postive
CXCR2	TLR4	0.553844212	4.27E-40	postive
ETS1	TLR4	0.596080986	1.07E-47	postive
TLR5	TLR5	1	0	postive
CD274	TLR8	0.633910914	1.56E-55	postive
CLEC4E	TLR8	0.676564152	8.70E-66	postive
CXCR2	TLR8	0.570363769	6.13E-43	postive
ETS1	TLR8	0.624178089	2.05E-53	postive

NLRC5	TLR8	0.503601288	2.33E-32	postive
ETS1	TM4SF18	0.527730938	6.50E-36	postive
CEACAM6	TM4SF20	0.50833612	4.92E-33	postive
ARRDC3	TM6SF1	0.560810457	2.82E-41	postive
ETS1	TM6SF1	0.600169179	1.70E-48	postive
NLRC5	TMC8	0.574263694	1.24E-43	postive
TRAF1	TMC8	0.561729855	1.96E-41	postive
CD274	TMEM140	0.514528349	6.21E-34	postive
ETS1	TMEM140	0.539445909	9.59E-38	postive
NLRC5	TMEM140	0.525696126	1.33E-35	postive
CLEC4E	TMEM154	0.574711493	1.03E-43	postive
CXCR2	TMEM154	0.603280052	4.14E-49	postive
HIF1A	TMEM158	0.514805864	5.65E-34	postive
ITGA5	TMEM158	0.519764164	1.04E-34	postive
CEACAM1	TMEM45B	0.513134963	9.93E-34	postive
CEACAM6	TMEM45B	0.558641879	6.62E-41	postive
CLEC4E	TMEM71	0.543956821	1.81E-38	postive
CXCR2	TMEM71	0.589057372	2.35E-46	postive
ETS1	TMEM71	0.511792301	1.56E-33	postive
ITGA5	TMEM71	0.508736134	4.31E-33	postive
ITGA5	TNC	0.758117739	3.61E-91	postive
LATS2	TNC	0.505072638	1.44E-32	postive
CD274	TNFAIP3	0.561956888	1.80E-41	postive
CLEC4E	TNFAIP3	0.566735805	2.66E-42	postive
ETS1	TNFAIP3	0.574174533	1.28E-43	postive
IL1B	TNFAIP3	0.517709881	2.11E-34	postive
TRAF1	TNFAIP3	0.560861339	2.77E-41	postive
ADM	TNFAIP6	0.581686875	5.55E-45	postive
CD274	TNFAIP6	0.510039136	2.80E-33	postive
CLEC4E	TNFAIP6	0.623776554	2.50E-53	postive
CXCR2	TNFAIP6	0.537145316	2.22E-37	postive
HIF1A	TNFAIP6	0.556568121	1.49E-40	postive
IL1B	TNFAIP6	0.523887937	2.50E-35	postive
ITGA5	TNFAIP6	0.506586604	8.77E-33	postive
ETS1	TNFAIP8	0.560693711	2.96E-41	postive
ETS1	TNFAIP8L2	0.596306563	9.65E-48	postive
TRAF1	TNFAIP8L2	0.515355195	4.70E-34	postive
CXCR2	TNFRSF10C	0.606243636	1.06E-49	postive
GKN1	TNFRSF17	0.531362039	1.79E-36	postive
CLEC4E	TNFRSF1B	0.583016172	3.15E-45	postive
CXCR2	TNFRSF1B	0.520414214	8.34E-35	postive
ETS1	TNFRSF1B	0.513126901	9.96E-34	postive
TRAF1	TNFRSF1B	0.596523042	8.76E-48	postive

TRAF1	TNFRSF4	0.503435905	2.46E-32	postive
CD274	TNFRSF9	0.630185839	1.03E-54	postive
CLEC4E	TNFRSF9	0.534002616	6.94E-37	postive
TRAF1	TNFRSF9	0.544856953	1.29E-38	postive
CD274	TNFSF13B	0.596927467	7.31E-48	postive
CLEC4E	TNFSF13B	0.504872703	1.54E-32	postive
ETS1	TNFSF13B	0.56853882	1.29E-42	postive
NLRC5	TNFSF13B	0.63360022	1.83E-55	postive
CLEC4E	TNFSF8	0.525099602	1.64E-35	postive
ETS1	TNFSF8	0.617895751	4.37E-52	postive
NLRC5	TNFSF8	0.508318457	4.95E-33	postive
TRAF1	TNFSF8	0.647794503	1.09E-58	postive
CLEC4E	TNIP3	0.537284428	2.11E-37	postive
ITGA5	TNS1	0.598332923	3.90E-48	postive
LATS2	TNS1	0.518501902	1.61E-34	postive
ITGA5	TOM1L1	-0.501679994	4.35E-32	negative
ARRDC3	TOMM34	-0.558444492	7.15E-41	negative
TRAP1	TOMM40	0.505208194	1.38E-32	postive
ETS1	TOX2	0.524833589	1.80E-35	postive
TRAF1	TOX2	0.5053197	1.33E-32	postive
ITGA5	TPM2	0.52842095	5.10E-36	postive
ITGB1	TPM4	0.515031609	5.24E-34	postive
TLR5	TPX2	-0.529604461	3.35E-36	negative
ETS1	TRAC	0.567732928	1.78E-42	postive
NLRC5	TRAC	0.598503057	3.61E-48	postive
TRAF1	TRAC	0.51260245	1.19E-33	postive
TRAF2	TRAF2	1	0	postive
ETS1	TRAF3IP3	0.558051976	8.34E-41	postive
NLRC5	TRAF3IP3	0.58373514	2.32E-45	postive
TRAF1	TRAF3IP3	0.539157343	1.07E-37	postive
ETS1	TRAT1	0.507917889	5.65E-33	postive
NLRC5	TRAT1	0.562086961	1.70E-41	postive
ETS1	TRBC1	0.529288733	3.75E-36	postive
NLRC5	TRBC1	0.609494841	2.35E-50	postive
TRAF1	TRBC1	0.548937054	2.78E-39	postive
CD274	TRDV3	0.50861329	4.49E-33	postive
NLRC5	TRDV3	0.556216036	1.71E-40	postive
ADM	TREM1	0.694354359	1.35E-70	postive
CLEC4E	TREM1	0.655292635	1.83E-60	postive
CXCR2	TREM1	0.62744091	4.08E-54	postive
IL1B	TREM1	0.686976822	1.47E-68	postive
NLRC5	TRG-AS1	0.565678215	4.07E-42	postive
TRAF1	TRG-AS1	0.519092984	1.31E-34	postive

CEACAM1	TRIM15	0.50288777	2.94E-32	postive
CEACAM6	TRIM15	0.505865825	1.11E-32	postive
ARRDC3	TRIM22	0.554489313	3.33E-40	postive
ETS1	TRIM22	0.554410553	3.43E-40	postive
NLRC5	TRIM22	0.629804186	1.25E-54	postive
ARRDC3	TRIM23	0.553318482	5.23E-40	postive
ITGB1	TRIM23	0.558150913	8.02E-41	postive
MMP10	TRPA1	0.500629557	6.11E-32	postive
ITGA5	TRPC1	0.507630865	6.22E-33	postive
PDIA3	TRPC1	-0.507727959	6.02E-33	negative
ITGA5	TRPC4	0.52200704	4.81E-35	postive
HIF1A	TRPC6	0.506265906	9.75E-33	postive
ITGA5	TSHZ3	0.667424623	1.91E-63	postive
LATS2	TSHZ3	0.521863036	5.06E-35	postive
CEACAM6	TSPAN1	0.557068169	1.22E-40	postive
PRTG	TSPAN18	0.532921963	1.02E-36	postive
ETS1	TSPAN4	0.548107572	3.81E-39	postive
ITGA5	TSPAN4	0.555776258	2.02E-40	postive
LATS2	TSPAN4	0.505587606	1.22E-32	postive
CEACAM6	TSPAN8	0.517059455	2.63E-34	postive
TRAP1	TTC28	-0.51758965	2.20E-34	negative
ITGA5	TTC7B	0.570633426	5.49E-43	postive
ITGA5	TUBA1A	0.616784138	7.45E-52	postive
ITGA5	TUBB6	0.712896774	5.39E-76	postive
ITGB1	TUBB6	0.502501244	3.33E-32	postive
LATS2	TUBB6	0.574683304	1.04E-43	postive
ITGA5	TWIST2	0.661228203	6.61E-62	postive
LATS2	TWIST2	0.521458926	5.82E-35	postive
ITGB1	TWSG1	0.550327679	1.64E-39	postive
CD274	TYMP	0.685220333	4.40E-68	postive
CLEC4E	TYMP	0.513332514	9.29E-34	postive
HPSE	TYMP	0.524344606	2.13E-35	postive
NLRC5	TYMP	0.55762204	9.87E-41	postive
CLEC4E	TYROBP	0.538892709	1.17E-37	postive
ETS1	TYROBP	0.567947912	1.63E-42	postive
ETS1	UBASH3A	0.505274082	1.35E-32	postive
NLRC5	UBASH3A	0.545524023	1.01E-38	postive
TRAF1	UBASH3A	0.5029359	2.89E-32	postive
CD274	UBE2L6	0.621215583	8.76E-53	postive
NLRC5	UBE2L6	0.680237352	9.44E-67	postive
IL17RB	UBE2T	0.518536426	1.59E-34	postive
GKN1	UGT2B15	0.600365385	1.56E-48	postive
GKN1	UPK1B	0.583416012	2.66E-45	postive

USF1	USF1	1	0	postive
CEACAM6	USH1C	0.559261416	5.19E-41	postive
CD274	USP30-AS1	0.524237084	2.22E-35	postive
NLRC5	USP30-AS1	0.61571718	1.24E-51	postive
ETS1	VAMP5	0.514992812	5.31E-34	postive
HIF1A	VASN	0.504961749	1.50E-32	postive
ITGA5	VASN	0.567624081	1.86E-42	postive
LATS2	VASN	0.513026755	1.03E-33	postive
CD274	VAV1	0.529358884	3.65E-36	postive
CLEC4E	VAV1	0.52440643	2.09E-35	postive
ETS1	VAV1	0.581009799	7.39E-45	postive
TRAF1	VAV1	0.544552259	1.45E-38	postive
ETS1	VCAM1	0.607751729	5.29E-50	postive
TRAF1	VCAM1	0.509019891	3.93E-33	postive
ITGA5	VCAN	0.541558566	4.40E-38	postive
ETS1	VEGFC	0.54912896	2.59E-39	postive
ITGA5	VEGFC	0.614209549	2.55E-51	postive
LATS2	VEGFC	0.533347621	8.79E-37	postive
ITGA5	VGLL3	0.559733102	4.32E-41	postive
LATS2	VGLL3	0.534206895	6.45E-37	postive
CEACAM6	VIL1	0.513839234	7.84E-34	postive
ETS1	VIM	0.520438347	8.27E-35	postive
ITGA5	VIM	0.625652266	9.91E-54	postive
LATS2	VIM	0.509836074	2.99E-33	postive
CXCR2	VNN2	0.546488131	7.01E-39	postive
GKN1	VSIG1	0.675973359	1.24E-65	postive
GKN1	VSIG2	0.673399722	5.75E-65	postive
ITGA5	VSTM4	0.64543407	3.84E-58	postive
LATS2	VSTM4	0.515915992	3.88E-34	postive
ETS1	VWF	0.510782533	2.18E-33	postive
ITGA5	VWF	0.585077832	1.31E-45	postive
CD274	WARS	0.707583749	2.10E-74	postive
NLRC5	WARS	0.640433431	5.38E-57	postive
TRAP1	WDR4	0.520185012	9.02E-35	postive
ARRDC3	WIPF1	0.534827666	5.16E-37	postive
CD274	WIPF1	0.509212487	3.68E-33	postive
CLEC4E	WIPF1	0.527728459	6.51E-36	postive
ETS1	WIPF1	0.731642244	6.57E-82	postive
NLRC5	WIPF1	0.543216047	2.38E-38	postive
TRAF1	WIPF1	0.576402524	5.10E-44	postive
ETS1	WISP1	0.500507618	6.36E-32	postive
ITGA5	WISP1	0.647529427	1.25E-58	postive
MMP10	WNT5A	0.525896109	1.24E-35	postive

LATS2	WWTR1	0.589813221	1.69E-46	postive
NLRC5	XAF1	0.654286125	3.18E-60	postive
TRAP1	YDJC	0.532286582	1.29E-36	postive
NLRC5	ZAP70	0.509267438	3.62E-33	postive
TRAF1	ZAP70	0.513643961	8.37E-34	postive
CD274	ZBED2	0.552675408	6.69E-40	postive
NLRC5	ZBED2	0.5141566	7.04E-34	postive
CD274	ZBP1	0.55483245	2.91E-40	postive
NLRC5	ZBP1	0.581820693	5.24E-45	postive
IL17RB	ZBTB4	-0.527515499	7.02E-36	negative
IL17RB	ZCCHC24	-0.506538446	8.91E-33	negative
ITGA5	ZCCHC24	0.567253905	2.16E-42	postive
ARRDC3	ZEB2	0.529615587	3.34E-36	postive
ETS1	ZEB2	0.616630897	8.02E-52	postive
ITGA5	ZEB2	0.567290936	2.13E-42	postive
NR4A2	ZFP36	0.586596101	6.81E-46	postive
ITGA5	ZFPM2	0.531234179	1.87E-36	postive
PDIA3	ZMAT1	-0.501368025	4.81E-32	negative
TLR5	ZMAT1	0.532122378	1.36E-36	postive
TRAF2	ZMYND19	0.552549451	7.02E-40	postive
ARRDC3	ZNF25	0.524107131	2.32E-35	postive
ITGA5	ZNF385D	0.529461779	3.52E-36	postive
ITGA5	ZNF423	0.524952763	1.73E-35	postive
HIF1A	ZNF469	0.5175262	2.24E-34	postive
ITGA5	ZNF469	0.585680325	1.01E-45	postive
ITGA5	ZNF521	0.546077179	8.18E-39	postive
ITGA5	ZNF532	0.545169025	1.15E-38	postive
ITGA5	ZNF542P	0.519211937	1.26E-34	postive
TRAP1	ZNF593	0.508337156	4.92E-33	postive
IL17RB	ZWINT	0.537492634	1.96E-37	postive
ITGA5	ZYX	0.577849352	2.79E-44	postive

Table.3 DEGs of HP-related prognostic gene modification groups.

gene	logFC	AveExpr	t	P.Value	adj.P.Val	B
ZCCHC24	1.407527	8.535903	16.54693	4.63E-49	1.00E-44	100.7597
RBMS3	1.041956	5.896617	16.16083	2.74E-47	2.97E-43	96.71976
MIR100HG	2.152192	6.796221	16.10977	4.69E-47	3.39E-43	96.18759
MPDZ	1.268154	7.555285	16.05661	8.20E-47	4.44E-43	95.63406
EML1	1.457893	7.184108	15.96939	2.05E-46	8.88E-43	94.72697
DZIP1	1.19957	5.819204	15.93639	2.90E-46	1.05E-42	94.38416
C14orf132	1.419563	6.579342	15.90797	3.91E-46	1.21E-42	94.0891
AOC3	1.877745	8.152566	15.78457	1.42E-45	3.85E-42	92.80998
ZNF423	1.400286	6.83778	15.75345	1.97E-45	4.74E-42	92.4878
CRISPLD1	1.80801	7.074921	15.6626	5.09E-45	1.10E-41	91.5486
LTBP1	1.241403	9.045929	15.62904	7.22E-45	1.42E-41	91.20217
SPARCL1	1.444818	12.03754	15.60912	8.88E-45	1.60E-41	90.99663
FBLN1	1.388232	7.448739	15.56707	1.38E-44	2.29E-41	90.56299
MGP	1.762759	8.713445	15.53517	1.92E-44	2.97E-41	90.23424
MFAP4	1.920967	7.763441	15.4312	5.65E-44	7.65E-41	89.16444
NAP1L3	1.825384	5.762124	15.38144	9.48E-44	1.18E-40	88.65336
FERMT2	1.385478	8.674778	15.37788	9.83E-44	1.18E-40	88.61678
RUNX1T1	1.124683	5.506546	15.36693	1.10E-43	1.26E-40	88.50438
TMEM47	1.356638	9.143077	15.25731	3.43E-43	3.45E-40	87.38065
FAM13C	1.080671	5.623659	15.25519	3.50E-43	3.45E-40	87.35902
GPRASP1	1.569229	6.396126	15.23064	4.52E-43	4.25E-40	87.10776
SETBP1	1.468706	6.924287	15.17517	8.01E-43	6.76E-40	86.54052
RCAN2	1.457976	8.508543	15.17388	8.12E-43	6.76E-40	86.52732
TRPC1	1.162626	5.809865	15.15259	1.01E-42	8.11E-40	86.30987
FBLN5	1.422252	7.533495	15.11578	1.48E-42	1.14E-39	85.93397
COL14A1	1.297308	6.0499	15.10571	1.64E-42	1.22E-39	85.83126
SLC24A3	1.227798	7.552381	15.09933	1.75E-42	1.26E-39	85.76615
EFEMP2	1.013789	8.334201	15.06154	2.59E-42	1.78E-39	85.38089
ANK2	1.174326	5.276806	15.05993	2.63E-42	1.78E-39	85.36448
ROBO1	1.086791	9.405301	15.04128	3.19E-42	2.09E-39	85.17436
HMCN1	1.578724	6.445371	15.00561	4.60E-42	2.93E-39	84.81118
PDE1A	1.06166	5.373834	14.94292	8.76E-42	5.42E-39	84.17354
FXYD6	1.311533	7.79439	14.89817	1.39E-41	8.11E-39	83.71905
GUCY1A3	1.509257	8.144204	14.88913	1.52E-41	8.44E-39	83.62731
MAN1C1	1.02327	7.224646	14.84602	2.37E-41	1.25E-38	83.18992
NR2F1	1.487076	8.150648	14.82943	2.80E-41	1.45E-38	83.02175
EPHA3	1.218838	5.323756	14.81029	3.41E-41	1.68E-38	82.82785

OGN	2.839469	6.459811	14.79921	3.82E-41	1.84E-38	82.71561
PKD2	1.086042	8.357941	14.79054	4.17E-41	1.96E-38	82.62785
LHFP	1.160653	8.341135	14.76975	5.16E-41	2.38E-38	82.41742
EFEMP1	1.44464	7.846595	14.7456	6.61E-41	2.98E-38	82.1731
SRPX	1.686481	7.942907	14.74115	6.92E-41	3.06E-38	82.12807
FHL1	1.880577	8.709848	14.68357	1.25E-40	5.39E-38	81.54619
DPYSL3	1.391504	7.260072	14.6709	1.42E-40	5.92E-38	81.41833
SYNPO2	2.013377	7.981392	14.67054	1.42E-40	5.92E-38	81.41472
NDN	1.386733	8.160688	14.66029	1.58E-40	6.45E-38	81.31125
FAT4	1.15865	7.39351	14.64894	1.77E-40	7.11E-38	81.19671
ZNF521	1.223368	6.583324	14.63859	1.97E-40	7.75E-38	81.09228
BOC	1.1124	6.850573	14.63604	2.02E-40	7.82E-38	81.06665
MN1	1.480001	6.760101	14.61133	2.60E-40	9.88E-38	80.81751
DDR2	1.127684	7.521434	14.5833	3.46E-40	1.25E-37	80.5351
SERPINF1	1.349639	9.716898	14.47161	1.08E-39	3.82E-37	79.41194
LINC01279	2.208654	7.18845	14.43128	1.62E-39	5.57E-37	79.00724
JAM2	1.346716	7.020799	14.39647	2.30E-39	7.45E-37	78.65819
STON1	1.169694	6.340784	14.38142	2.68E-39	8.55E-37	78.50747
ZFPM2	1.60027	6.101772	14.35148	3.63E-39	1.12E-36	78.2077
ARMCX1	1.308871	8.322933	14.33348	4.36E-39	1.31E-36	78.02763
MRGPRF	1.715507	7.605276	14.33154	4.44E-39	1.32E-36	78.0082
CHRDL1	2.1915	6.72211	14.28249	7.29E-39	2.13E-36	77.51793
ECM2	1.076838	7.191512	14.24967	1.02E-38	2.93E-36	77.19027
FOXF1	1.307992	9.111238	14.21969	1.37E-38	3.86E-36	76.89119
RP11-999E24.3	1.162459	4.74862	14.21558	1.43E-38	3.98E-36	76.85021
GHR	1.575685	5.61503	14.20992	1.52E-38	4.10E-36	76.79373
TGFB1I1	1.297153	9.530212	14.17865	2.08E-38	5.42E-36	76.48216
RNF150	1.175111	5.601269	14.16049	2.49E-38	6.43E-36	76.30135
CXCL3	-1.65591	9.860868	-14.146	2.89E-38	7.35E-36	76.15678
MYLK	1.255752	8.181163	14.12397	3.60E-38	8.96E-36	75.93804
MRVI1	1.24404	7.298973	14.12073	3.72E-38	9.15E-36	75.90582
MSRB3	1.353854	7.677055	14.1029	4.45E-38	1.06E-35	75.72861
COX7A1	1.244669	8.416851	14.07975	5.61E-38	1.30E-35	75.49869
OLFML1	1.121314	7.325235	14.07925	5.64E-38	1.30E-35	75.49367
PRICKLE2	1.251858	7.031427	14.07255	6.03E-38	1.38E-35	75.42719
PDLIM3	1.330268	7.284612	14.02705	9.52E-38	2.15E-35	74.97576
TAGLN	1.432675	9.970663	14.01944	1.03E-37	2.29E-35	74.9003
BNC2	1.066536	5.861576	14.01411	1.08E-37	2.40E-35	74.84743
DCLK1	1.242403	5.443692	13.99827	1.27E-37	2.78E-35	74.69046
RERG	1.543752	6.782554	13.95152	2.03E-37	4.35E-35	74.22767
SLIT2	1.412391	6.321971	13.92501	2.64E-37	5.61E-35	73.96547
SCN4B	1.590549	5.098557	13.89642	3.52E-37	7.39E-35	73.68302
FOXP2	1.060361	4.948521	13.86179	4.97E-37	1.03E-34	73.34118

BHMT2	1.018144	4.918085	13.81535	7.89E-37	1.63E-34	72.88336
CNRIP1	1.102207	8.387738	13.81084	8.25E-37	1.69E-34	72.839
LMO3	1.12175	4.453879	13.78244	1.10E-36	2.22E-34	72.55936
SPON1	1.392124	7.543733	13.77918	1.13E-36	2.27E-34	72.52728
PLSCR4	1.233658	8.360546	13.75052	1.50E-36	2.96E-34	72.24536
HSPB2	1.205356	6.027783	13.73925	1.68E-36	3.28E-34	72.1346
IGF1	1.370411	6.469605	13.73102	1.83E-36	3.53E-34	72.05367
NEGR1	1.321572	5.569741	13.70658	2.33E-36	4.42E-34	71.8137
LRRN4CL	1.170862	6.56203	13.68876	2.78E-36	5.14E-34	71.63876
CCDC80	1.62294	7.335464	13.68674	2.83E-36	5.20E-34	71.61897
SDPR	1.373474	7.19161	13.63536	4.71E-36	8.58E-34	71.11522
PDZRN3	1.165638	6.965434	13.62989	4.98E-36	8.91E-34	71.06167
PLN	2.186205	7.084811	13.6072	6.23E-36	1.11E-33	70.83956
DAAM2	1.103381	6.907571	13.60597	6.31E-36	1.11E-33	70.82745
CSRP2	1.036212	9.16747	13.5964	6.93E-36	1.21E-33	70.73384
FBN1	1.233721	8.503122	13.58024	8.13E-36	1.41E-33	70.57581
CPED1	1.124489	7.758411	13.55223	1.07E-35	1.81E-33	70.302
DCN	1.084714	9.369768	13.53767	1.24E-35	2.06E-33	70.1598
TCEAL7	1.50966	6.574249	13.52129	1.46E-35	2.39E-33	69.99996
OLFML3	1.284561	8.563341	13.51827	1.50E-35	2.44E-33	69.97046
RGMA	1.015079	6.588652	13.51408	1.56E-35	2.51E-33	69.92962
CRYAB	1.45767	8.014737	13.50769	1.66E-35	2.65E-33	69.86726
TNS1	1.012282	7.7104	13.49049	1.97E-35	3.12E-33	69.69947
FRZB	1.204251	6.566387	13.48666	2.05E-35	3.21E-33	69.66222
C2orf40	2.721733	6.817225	13.48502	2.08E-35	3.24E-33	69.64619
CNN1	2.205031	8.861198	13.4639	2.56E-35	3.96E-33	69.44043
AK021804	1.593805	5.961914	13.45812	2.71E-35	4.17E-33	69.38411
GLT8D2	1.192473	7.984187	13.44516	3.08E-35	4.70E-33	69.2579
RSPO3	1.817694	8.172233	13.39989	4.81E-35	7.23E-33	68.81761
MYH11	1.772637	7.831695	13.38597	5.51E-35	8.24E-33	68.68231
C7	1.991542	6.615591	13.38424	5.61E-35	8.32E-33	68.66557
ZNF667-AS1	1.168801	6.460863	13.35878	7.20E-35	1.06E-32	68.41828
PEG3	1.375735	5.232046	13.35111	7.76E-35	1.14E-32	68.34388
SGCE	1.290681	8.698625	13.3472	8.07E-35	1.17E-32	68.30598
PRELP	1.153398	6.797297	13.3317	9.39E-35	1.36E-32	68.15565
SMAD9	1.11109	6.667078	13.31459	1.11E-34	1.58E-32	67.98978
TUBB6	1.091876	10.08602	13.25639	1.96E-34	2.78E-32	67.42629
MAP1B	1.149649	7.548015	13.25434	2.00E-34	2.82E-32	67.40645
LOXL1	1.015858	8.307467	13.2535	2.02E-34	2.82E-32	67.39836
PPP1R14A	1.628734	7.11135	13.23027	2.53E-34	3.52E-32	67.17379
SYNM	2.147833	8.909667	13.18177	4.07E-34	5.54E-32	66.70556
EMILIN1	1.096541	7.589861	13.18119	4.09E-34	5.54E-32	66.69998
GPR133	1.545666	5.757442	13.13809	6.23E-34	8.32E-32	66.28458

CPE	1.558403	8.609333	13.12361	7.17E-34	9.53E-32	66.14512
ITIH5	1.111547	6.87074	13.11817	7.56E-34	9.98E-32	66.09274
ACTG2	1.640258	8.704275	13.09902	9.11E-34	1.19E-31	65.90848
HSPB8	1.307799	7.314971	12.99011	2.62E-33	3.32E-31	64.86317
KGFLP2	1.059796	5.399113	12.98961	2.63E-33	3.32E-31	64.85837
RBPMS2	1.640134	6.868632	12.93995	4.26E-33	5.21E-31	64.38303
ABI3BP	1.249681	6.13215	12.92601	4.87E-33	5.93E-31	64.24982
TIMP3	1.084038	8.659428	12.91669	5.33E-33	6.41E-31	64.16081
CDH11	1.044397	8.301491	12.90154	6.17E-33	7.38E-31	64.0161
AEBP1	1.228312	9.237747	12.88744	7.07E-33	8.41E-31	63.88152
GUCY1B3	1.136439	8.14664	12.86237	9.01E-33	1.06E-30	63.64237
ITGA7	1.289087	6.192623	12.85183	9.97E-33	1.17E-30	63.54188
C8orf88	1.367699	5.808213	12.84112	1.11E-32	1.29E-30	63.43988
WASF3	1.310141	7.274335	12.83448	1.18E-32	1.36E-30	63.37662
SMOC2	1.379597	7.778175	12.83327	1.19E-32	1.37E-30	63.36511
TCF21	1.018193	6.523674	12.8125	1.46E-32	1.66E-30	63.16737
MYL9	1.328281	8.044253	12.76867	2.22E-32	2.48E-30	62.75065
PGM5	1.337651	5.898735	12.75116	2.63E-32	2.90E-30	62.58432
PLAC9	1.161274	5.882559	12.73149	3.17E-32	3.45E-30	62.39765
LOC728392	1.029795	7.014385	12.73048	3.20E-32	3.47E-30	62.38807
LMOD1	1.67157	7.13137	12.72423	3.40E-32	3.66E-30	62.32883
MAMDC2	1.842672	5.582538	12.72356	3.42E-32	3.67E-30	62.32249
MEOX2	1.446191	4.365246	12.70764	3.99E-32	4.23E-30	62.17154
C1QTNF7	1.318334	5.284333	12.6806	5.16E-32	5.43E-30	61.91541
CYP1B1	1.515987	7.348842	12.62808	8.54E-32	8.84E-30	61.41867
C1QTNF2	1.432159	5.362062	12.61536	9.64E-32	9.94E-30	61.2985
MYH10	1.173597	8.247574	12.59751	1.14E-31	1.17E-29	61.13002
MYOCD	1.624912	6.914718	12.59142	1.21E-31	1.23E-29	61.07262
LRCH2	1.038352	5.629543	12.58478	1.29E-31	1.30E-29	61.00993
FIBIN	1.103096	7.333176	12.57696	1.39E-31	1.39E-29	60.93616
PTGIS	1.01569	6.209157	12.53266	2.12E-31	2.11E-29	60.51885
CFL2	1.280765	8.262744	12.50842	2.67E-31	2.63E-29	60.29089
OMD	1.587504	5.45639	12.50493	2.76E-31	2.71E-29	60.25804
APOD	1.737883	8.210105	12.47483	3.68E-31	3.54E-29	59.97522
RAI2	1.238601	7.374154	12.46423	4.06E-31	3.88E-29	59.8757
ITGBL1	1.375789	6.126175	12.45628	4.38E-31	4.16E-29	59.80115
ELOVL4	1.027365	4.815406	12.44592	4.84E-31	4.57E-29	59.70391
FILIP1	1.260667	5.734128	12.44239	5.00E-31	4.69E-29	59.67089
KCTD12	1.036417	10.61162	12.41948	6.21E-31	5.71E-29	59.45612
PRKAR2B	1.309245	8.38064	12.40557	7.09E-31	6.48E-29	59.32584
ARMCX2	1.229513	8.209941	12.38038	9.00E-31	8.05E-29	59.09011
CAP2	1.266086	7.159794	12.37141	9.79E-31	8.69E-29	59.00623
CACNA2D1	1.00222	6.782407	12.33628	1.37E-30	1.19E-28	58.67804

CLIP3	1.072412	5.984681	12.31444	1.68E-30	1.44E-28	58.47417
SCRG1	1.688118	5.633318	12.29581	2.00E-30	1.70E-28	58.30049
SORBS1	1.006579	7.706876	12.29041	2.10E-30	1.77E-28	58.25022
EPHA7	1.313991	4.600515	12.27437	2.45E-30	2.05E-28	58.10082
SCN7A	1.456174	4.566527	12.26396	2.70E-30	2.25E-28	58.00389
CXCL2	-1.04912	7.016072	-12.2545	2.95E-30	2.44E-28	57.91557
FRMD6	1.137964	8.866787	12.23435	3.57E-30	2.92E-28	57.72844
THBS4	1.81163	7.942004	12.21635	4.23E-30	3.41E-28	57.56117
INMT	1.136937	6.563126	12.20878	4.54E-30	3.65E-28	57.49088
CAV1	1.114778	10.59553	12.19911	4.97E-30	3.96E-28	57.40109
C3orf70	1.304795	7.219927	12.19089	5.37E-30	4.24E-28	57.32479
CYBRD1	1.27301	8.868695	12.1574	7.35E-30	5.70E-28	57.01431
PDE3A	1.04001	6.350589	12.14978	7.89E-30	6.08E-28	56.94372
ASPN	1.623448	9.42344	12.14224	8.47E-30	6.50E-28	56.87395
COLEC12	1.363387	7.454713	12.13257	9.27E-30	7.05E-28	56.78437
SYNC	1.471658	6.858165	12.09382	1.33E-29	1.00E-27	56.42613
BCHE	1.817562	5.093359	12.0897	1.38E-29	1.04E-27	56.38804
DNM3OS	1.053044	5.395824	12.08346	1.47E-29	1.10E-27	56.33039
CXCL12	1.35975	8.128499	12.06512	1.74E-29	1.29E-27	56.16107
PNMAL1	1.34694	5.710692	12.06218	1.79E-29	1.32E-27	56.13395
ANGPTL1	1.697954	5.07331	12.05523	1.91E-29	1.40E-27	56.06983
CH25H	1.216568	7.444921	11.97515	4.03E-29	2.84E-27	55.33252
TSPAN7	1.165944	7.431123	11.94283	5.44E-29	3.78E-27	55.03576
CLMP	1.28507	6.972156	11.93521	5.84E-29	4.03E-27	54.96581
ABCA8	1.290002	5.697691	11.90866	7.47E-29	5.07E-27	54.7224
TMEM35	1.248877	5.370444	11.90304	7.87E-29	5.33E-27	54.67089
C1R	1.056509	10.23403	11.87799	9.93E-29	6.65E-27	54.44156
MIR143HG	1.04836	5.98352	11.87397	1.03E-28	6.89E-27	54.40483
ROR2	1.040297	5.58097	11.86852	1.08E-28	7.22E-27	54.35492
KCNJ8	1.045123	7.129209	11.8655	1.11E-28	7.40E-27	54.32736
MFAP5	1.529726	7.139394	11.85696	1.21E-28	7.99E-27	54.24926
FOXF2	1.141252	8.355582	11.85603	1.22E-28	8.03E-27	54.24071
GREM2	1.219764	4.882119	11.84159	1.39E-28	9.15E-27	54.10878
MEIS1	1.086302	7.57417	11.8268	1.59E-28	1.04E-26	53.97374
COL8A1	1.116704	6.51836	11.77927	2.47E-28	1.59E-26	53.54031
NEXN	1.465845	8.42107	11.75692	3.04E-28	1.94E-26	53.33692
RBM24	1.293909	5.310533	11.73576	3.69E-28	2.33E-26	53.14446
AKAP12	1.135128	7.078288	11.71416	4.50E-28	2.82E-26	52.94823
ABCA6	1.002779	4.922557	11.70363	4.96E-28	3.09E-26	52.8526
PPP1R3C	1.161842	6.167054	11.68164	6.06E-28	3.75E-26	52.65319
CILP	1.372142	7.279827	11.67957	6.18E-28	3.81E-26	52.63441
ISLR	1.072247	7.349855	11.67668	6.35E-28	3.91E-26	52.60817
SFRP4	1.93522	7.660543	11.6653	7.05E-28	4.32E-26	52.50504

PABPC5	1.063003	3.411505	11.62449	1.02E-27	6.21E-26	52.13575
KCNMB1	1.044077	6.066534	11.59267	1.37E-27	8.15E-26	51.84822
CHRDL2	1.591036	6.560696	11.56903	1.70E-27	9.98E-26	51.63491
MICU3	1.250742	4.995583	11.56737	1.73E-27	1.01E-25	51.62002
TSPAN2	1.014381	6.516894	11.5616	1.82E-27	1.06E-25	51.56798
DACT1	1.169613	8.013597	11.53287	2.36E-27	1.35E-25	51.30916
FLNA	1.048397	8.744599	11.42683	6.20E-27	3.45E-25	50.35714
GREM1	1.784136	10.53368	11.39039	8.62E-27	4.76E-25	50.03118
NRXN3	1.471655	5.827272	11.38619	8.95E-27	4.93E-25	49.99369
BEX4	1.172932	7.966415	11.36286	1.11E-26	6.02E-25	49.78532
REEP1	1.357406	6.820869	11.36196	1.11E-26	6.05E-25	49.7773
TCEAL2	1.706366	5.384177	11.36025	1.13E-26	6.13E-25	49.76203
PCDH9	1.000051	4.558383	11.33013	1.49E-26	7.99E-25	49.49346
LOC101927943	1.029603	3.520247	11.32505	1.56E-26	8.32E-25	49.44813
ACKR1	1.598747	7.320742	11.29029	2.13E-26	1.12E-24	49.13878
RAB9B	1.129903	5.975738	11.28738	2.18E-26	1.14E-24	49.11291
SFRP1	1.195945	5.865298	11.27943	2.35E-26	1.22E-24	49.0422
FBLN2	1.317654	6.815638	11.2598	2.80E-26	1.44E-24	48.8679
MGC24103	1.025611	4.978301	11.25358	2.96E-26	1.52E-24	48.81269
FABP4	1.45434	5.967605	11.19876	4.84E-26	2.43E-24	48.32677
LOC100507165	1.203208	4.82524	11.18736	5.36E-26	2.68E-24	48.22589
RAB23	1.006217	8.613284	11.18134	5.66E-26	2.81E-24	48.1727
DES	1.617409	6.714607	11.15525	7.15E-26	3.50E-24	47.94214
PRIMA1	1.460185	6.484027	11.13182	8.81E-26	4.30E-24	47.73543
TMEM178A	1.051615	5.29695	11.12156	9.66E-26	4.68E-24	47.6449
KCNMA1	1.0931	6.010813	11.11761	1.00E-25	4.84E-24	47.61009
NAP1L2	1.178015	4.87087	11.10887	1.08E-25	5.21E-24	47.53309
PDZRN4	1.732579	4.987365	11.08393	1.35E-25	6.45E-24	47.31355
RP11-305O6.3	1.125277	4.312541	11.07584	1.45E-25	6.90E-24	47.24242
HSPB6	1.151751	5.436838	11.05793	1.70E-25	8.05E-24	47.08503
CASQ2	1.630858	5.697825	10.96664	3.83E-25	1.75E-23	46.28509
COL21A1	1.21906	6.574911	10.95937	4.08E-25	1.86E-23	46.22155
PCOLCE	1.106003	8.51006	10.95677	4.18E-25	1.90E-23	46.19881
GXYLT2	1.035467	6.70479	10.95145	4.38E-25	1.98E-23	46.15231
F13A1	1.348295	7.678746	10.92115	5.73E-25	2.57E-23	45.88789
CYS1	1.170857	5.601308	10.89879	6.98E-25	3.08E-23	45.69303
PDZK1IP1	-1.54719	10.14493	-10.8971	7.08E-25	3.12E-23	45.67878
SFRP2	1.994726	8.177994	10.87693	8.46E-25	3.70E-23	45.50282
CDH19	1.000628	4.578136	10.84226	1.15E-24	4.92E-23	45.20156
TMEM100	1.59881	6.984088	10.83581	1.21E-24	5.18E-23	45.14556
HSPA2	1.032015	7.950906	10.82183	1.37E-24	5.81E-23	45.02429
GPM6B	1.23084	6.001895	10.80082	1.65E-24	6.95E-23	44.8422
ADH1B	1.711297	6.12344	10.79011	1.81E-24	7.59E-23	44.74949

ATP1A2	1.36023	5.078929	10.74901	2.60E-24	1.07E-22	44.39412
PRUNE2	1.28213	6.468263	10.74856	2.61E-24	1.07E-22	44.39022
PGM5-AS1	1.594952	5.517672	10.73892	2.84E-24	1.16E-22	44.30699
KLHL13	1.351868	6.534435	10.72082	3.33E-24	1.36E-22	44.15089
CCDC69	1.149266	7.335432	10.71973	3.36E-24	1.37E-22	44.1415
C1S	1.030848	9.63234	10.71004	3.66E-24	1.48E-22	44.05802
TMEM170B	1.118943	5.779843	10.69326	4.23E-24	1.69E-22	43.91349
NRK	1.054791	4.400653	10.66807	5.27E-24	2.08E-22	43.69679
ISL1	1.199111	7.68661	10.66446	5.44E-24	2.14E-22	43.66571
SPOCK1	1.479237	7.73672	10.66172	5.57E-24	2.19E-22	43.64221
VGLL3	1.018169	6.041099	10.65489	5.91E-24	2.30E-22	43.58354
NBEA	1.143959	6.484894	10.63417	7.08E-24	2.73E-22	43.40569
PBK	-1.05134	9.433834	-10.6156	8.32E-24	3.18E-22	43.24669
FLNC	1.114975	8.046286	10.60623	9.03E-24	3.44E-22	43.16613
DPT	1.294968	6.280078	10.60582	9.06E-24	3.44E-22	43.1627
LINC00702	1.16976	4.603688	10.58588	1.08E-23	4.05E-22	42.99198
HSPB7	1.062781	5.535993	10.55565	1.40E-23	5.16E-22	42.73361
GGT5	1.107286	6.472675	10.54831	1.49E-23	5.47E-22	42.67091
STMN2	1.322277	5.243679	10.52787	1.78E-23	6.47E-22	42.49655
MBNL1-AS1	1.1314	6.731492	10.52761	1.78E-23	6.47E-22	42.49432
HAND2-AS1	1.540626	4.701173	10.52277	1.86E-23	6.73E-22	42.45306
HSD17B6	1.0572	6.275921	10.41189	4.83E-23	1.67E-21	41.51116
FMO2	1.01546	6.789075	10.39298	5.68E-23	1.96E-21	41.35121
KIT	1.116764	8.253084	10.34133	8.84E-23	3.00E-21	40.91506
ASB2	1.099352	6.060857	10.30429	1.21E-22	4.05E-21	40.60321
PTGFR	1.007711	4.836831	10.30155	1.24E-22	4.13E-21	40.58017
TTLL7	1.058162	5.331142	10.29742	1.29E-22	4.26E-21	40.54539
ZNF415	1.139659	5.426591	10.26949	1.63E-22	5.30E-21	40.31083
TNC	1.177951	7.291398	10.26708	1.66E-22	5.40E-21	40.29062
GGTA1P	1.04636	7.720015	10.216	2.57E-22	8.09E-21	39.86269
CCL20	-1.88006	10.36627	-10.191	3.18E-22	9.85E-21	39.65372
FAM129A	1.103387	9.13976	10.16023	4.12E-22	1.27E-20	39.39702
AGTR1	1.115041	5.017398	10.10512	6.56E-22	1.99E-20	38.93847
PLP1	1.320789	4.684322	10.03772	1.16E-21	3.42E-20	38.37981
POPDC2	1.183998	5.144355	9.871981	4.61E-21	1.26E-19	37.01668
THSD7B	1.0231	4.358776	9.821215	7.02E-21	1.89E-19	36.60214
FBXL22	1.33433	4.494055	9.705056	1.83E-20	4.69E-19	35.65899
CXCL1	-1.31954	10.26393	-9.6695	2.45E-20	6.22E-19	35.37179
MYOT	1.158833	4.215866	9.632053	3.32E-20	8.31E-19	35.07011
SUSD5	1.019719	4.109576	9.55919	6.01E-20	1.47E-18	34.48535
TYRP1	1.457378	6.863939	9.523061	8.06E-20	1.93E-18	34.19652
PDGFRL	1.18759	6.318208	9.522237	8.12E-20	1.94E-18	34.18994
TSPYL5	1.099604	7.902154	9.471202	1.23E-19	2.87E-18	33.78325

GAS1	1.135456	6.650601	9.450077	1.45E-19	3.38E-18	33.61534
PCDHB5	1.260964	4.57263	9.420156	1.85E-19	4.25E-18	33.37796
FGF13	1.148545	6.846265	9.418901	1.87E-19	4.29E-18	33.36801
PTGDS	1.018864	8.975997	9.325857	3.94E-19	8.64E-18	32.63323
ITGB1BP2	1.023085	5.379225	9.228604	8.56E-19	1.78E-17	31.87062
ASB5	1.49975	4.186122	9.209229	9.98E-19	2.05E-17	31.71936
SGCA	1.003811	4.357949	9.192896	1.14E-18	2.31E-17	31.59202
IL33	1.412761	8.092903	9.156856	1.51E-18	3.03E-17	31.3116
FNDC1	1.475778	7.618752	9.112836	2.14E-18	4.21E-17	30.97013
THBS2	1.243299	9.365068	9.083124	2.70E-18	5.27E-17	30.74031
VIP	1.60725	4.437471	8.976137	6.23E-18	1.17E-16	29.91717
CXCL8	-1.53383	10.56678	-8.95383	7.41E-18	1.38E-16	29.74644
FGF10	1.005085	4.315398	8.946035	7.87E-18	1.45E-16	29.68683
CFH	1.04543	8.714587	8.940014	8.25E-18	1.52E-16	29.64082
LCN2	-1.6853	11.85609	-8.91373	1.01E-17	1.85E-16	29.44027
SHISA3	1.215289	5.084547	8.791526	2.60E-17	4.52E-16	28.51324
MMRN1	1.054569	5.434968	8.750906	3.54E-17	6.07E-16	28.20715
TAC1	1.466125	4.458466	8.708067	4.92E-17	8.24E-16	27.88545
WFDC1	1.001096	8.484669	8.676734	6.24E-17	1.03E-15	27.6509
CPA3	1.308919	9.275288	8.617848	9.75E-17	1.57E-15	27.21174
C2CD4A	-1.20399	7.523894	-8.56294	1.48E-16	2.35E-15	26.80423
TMEM255A	1.075943	5.350813	8.496412	2.43E-16	3.76E-15	26.31303
PLEKHS1	-1.0946	6.315535	-8.35062	7.21E-16	1.06E-14	25.24652
CCL19	1.314448	7.798962	8.333306	8.19E-16	1.19E-14	25.12081
SLC6A14	-1.66036	8.758073	-8.19163	2.32E-15	3.21E-14	24.09927
MMP12	-1.47031	10.58509	-8.16474	2.82E-15	3.84E-14	23.90685
TRIM31	-1.02027	7.970419	-7.95424	1.29E-14	1.61E-13	22.41741
SCG2	1.004951	5.785705	7.879404	2.19E-14	2.66E-13	21.89505
IDO1	-1.22284	8.790368	-7.81976	3.34E-14	3.95E-13	21.48144
AGT	1.008632	9.157777	7.746002	5.61E-14	6.43E-13	20.97338
TACR2	1.102465	5.657393	7.742355	5.76E-14	6.58E-13	20.94835
FAM19A4	1.100315	3.881806	7.433766	4.83E-13	4.82E-12	18.86438
CCL11	1.078276	9.095624	7.275773	1.40E-12	1.31E-11	17.82344
HCAR3	-1.33617	7.352442	-7.07396	5.31E-12	4.60E-11	16.51994
HTR2B	1.198918	6.09815	6.93793	1.28E-11	1.06E-10	15.65816
CEACAM7	-1.12795	6.333654	-6.81228	2.86E-11	2.26E-10	14.8743
SMPX	1.39486	4.934527	6.80623	2.98E-11	2.34E-10	14.83685
SERPINB7	-1.08673	5.592784	-6.69254	6.09E-11	4.59E-10	14.13832
CXCL5	-1.23827	8.938089	-6.53986	1.57E-10	1.11E-09	13.21561
PI3	-1.05414	9.155595	-5.11328	4.57E-07	1.99E-06	5.48491
SLCO1B3	-1.02044	5.262156	-5.02356	7.15E-07	3.03E-06	5.054713
MMP3	-1.02171	9.223047	-4.5441	6.98E-06	2.51E-05	2.87286
BPIFB1	-1.14839	8.644634	-3.8302	0.000145	0.000413	-0.00073

Table.4 The detailed information of HP-related groups for the patients in the meta-cohort.

sample	fustat	Futime (years)	Age	Gender	Stage	HP score	Hp_score group	Hp_cluster	Risk	Stromal Score	Microenvironment Score
GSM1523727	alive	2.957778	79	M	II	-0.0112	low	cluster_1	low	-1030.22	-481.643
GSM1523728	alive	2.941111	70	M	II	0.015007	low	cluster_2	low	668.5165	2389.636
GSM1523729	alive	2.941111	70	M	II	0.029724	low	cluster_2	low	382.5918	1602.783
GSM1523744	alive	3.523333	79	F	II	0.000953	low	cluster_1	low	258.7765	1182.749
GSM1523745	alive	3.517778	77	M	III	0.024384	low	cluster_2	high	769.994	2181.165
GSM1523746	dead	0.85	66	M	II	0.03913	high	cluster_2	high	-244.873	686.3868
GSM1523747	dead	0.29	88	M	III	0.068493	high	cluster_2	high	-315.994	643.551
GSM1523748	alive	3.497778	41	F	III	0.037468	high	cluster_2	low	707.6402	2310.519
GSM1523765	dead	0.473333	70	M	III	0.034752	low	cluster_2	high	-11.1868	723.9986
GSM1523768	dead	0.807778	49	F	III	0.028767	low	cluster_2	high	1385.858	3409.101
GSM1523769	dead	0.457778	71	F	IV	0.092468	high	cluster_2	high	1500.244	4244.921
GSM1523770	dead	0.735556	76	F	II	-0.00826	low	cluster_1	low	372.8491	2521.366
GSM1523771	dead	0.422222	73	M	IV	0.116128	high	cluster_2	high	2398.481	4949.673
GSM1523772	dead	0.334444	74	M	IV	0.146295	high	cluster_2	high	1680.428	3373.385
GSM1523773	dead	0.144444	70	M	IV	0.122354	high	cluster_2	high	2158.943	4361.229
GSM1523774	alive	3.256667	67	F	III	0.053486	high	cluster_2	high	1092.208	2873.605
GSM1523775	alive	3.253333	68	F	II	0.021071	low	cluster_2	high	-148.953	972.7816
GSM1523776	alive	3.248889	65	M	II	-0.0162	low	cluster_1	low	248.3524	2185.597
GSM1523777	alive	3.097778	56	M	IV	0.044206	high	cluster_2	low	87.46861	486.2299
GSM1523778	alive	3.087778	82	F	III	0.065879	high	cluster_2	high	1309.019	3422.879
GSM1523779	alive	3.086667	64	M	III	0.009965	low	cluster_1	low	111.2109	1969.295
GSM1523780	dead	0.546667	46	F	IV	0.057867	high	cluster_2	high	930.4337	2352.692
GSM1523781	dead	0.097778	65	M	IV	0.066548	high	cluster_2	low	-701.29	-860.935
GSM1523782	dead	0.146667	23	M	IV	0.008695	low	cluster_1	low	336.6546	1459.503
GSM1523783	alive	3.064444	67	M	III	0.055467	high	cluster_2	low	341.5619	2890.033
GSM1523784	alive	3.043333	73	M	III	0.030663	low	cluster_2	low	762.5508	2888.49
GSM1523785	dead	0.392222	68	M	II	0.034713	low	cluster_2	low	682.2689	2434.021
GSM1523786	dead	0.948889	51	F	IV	0.05684	high	cluster_2	low	1109.454	3652.501
GSM1523787	alive	2.987778	70	F	IV	0.01671	low	cluster_2	low	-1227.58	-944.596
GSM1523788	dead	0.275556	60	F	IV	0.092965	high	cluster_2	high	2020.989	4331.712
GSM1523789	dead	0.393333	64	M	IV	0.098649	high	cluster_2	high	1413.399	2870.09
GSM1523790	alive	2.697778	73	M	II	-0.01731	low	cluster_1	low	1213.106	3726.536
GSM1523791	alive	2.704444	65	F	I	0.032548	low	cluster_1	low	845.7052	2881.794
GSM1523792	alive	2.693333	34	F	I	0.024264	low	cluster_2	low	-283.826	966.3517
GSM1523793	alive	2.691111	67	M	II	0.00457	low	cluster_2	low	226.4317	2030.392
GSM1523794	dead	0.9	69	M	IV	0.086624	high	cluster_2	low	965.1827	3298.708

GSM1523795	alive	2.668889	69	M	I	0.032404	low	cluster_2	low	436.1467	1732.442
GSM1523796	dead	1.921111	79	M	III	0.093088	high	cluster_2	high	2596.323	5361.638
GSM1523797	dead	0.942222	60	M	III	0.010713	low	cluster_2	low	-592.051	730.4371
GSM1523798	dead	2.061111	79	F	II	0.053193	high	cluster_2	low	1655.677	5361.317
GSM1523799	alive	2.583333	50	M	II	0.069498	high	cluster_2	high	420.4187	2322.698
GSM1523800	alive	2.564444	51	M	III	-0.02005	low	cluster_1	low	115.2697	2462.338
GSM1523801	alive	2.564444	40	M	II	-0.00657	low	cluster_1	low	-19.1796	1830.793
GSM1523802	alive	2.558889	64	M	II	0.015911	low	cluster_1	high	62.52591	1692.47
GSM1523803	dead	0.235556	39	F	IV	0.015791	low	cluster_2	low	222.4497	1676.2
GSM1523804	alive	2.556667	88	F	III	0.064989	high	cluster_2	high	1225.08	3518.27
GSM1523805	dead	0.148889	80	M	III	0.053714	high	cluster_2	low	1509.657	3899.018
GSM1523806	dead	0.423333	48	M	III	0.037241	high	cluster_2	low	276.6433	2127.313
GSM1523807	alive	2.457778	74	M	II	0.027814	low	cluster_2	high	959.6146	3169.17
GSM1523808	alive	2.45	54	F	III	0.065368	high	cluster_2	low	1465.308	3908.111
GSM1523809	alive	2.447778	61	M	II	0.021149	low	cluster_2	high	249.0415	2161.023
GSM1523810	alive	2.435556	76	F	I	0.039637	high	cluster_2	low	1099.44	4051.181
GSM1523811	alive	2.428889	74	F	I	0.022759	low	cluster_2	low	529.4199	2904.774
GSM1523812	alive	2.427778	80	M	III	0.049234	high	cluster_2	low	1571.887	4348.757
GSM1523813	alive	2.427778	77	M	III	0.064255	high	cluster_2	high	1000.965	2010.465
GSM1523814	alive	2.424444	72	M	II	0.027527	low	cluster_2	high	231.4756	1729.87
GSM1523815	dead	0.838889	59	M	III	0.104184	high	cluster_2	low	533.548	1702.388
GSM1523816	alive	2.402222	71	M	II	-0.00214	low	cluster_1	low	-688.604	142.6334
GSM1523817	dead	0.207778	66	M	IV	0.043165	high	cluster_2	high	88.98378	395.4267
GSM1523818	dead	0.327778	73	M	IV	0.012627	low	cluster_1	low	-384.026	1198.811
GSM1523819	dead	0.336667	65	F	IV	0.066025	high	cluster_2	high	-459.845	-52.9038
GSM1523820	dead	0.79	72	M	III	0.036994	high	cluster_2	low	-477.96	845.5547
GSM1523821	alive	2.37	60	M	IV	-0.02148	low	cluster_1	low	-178.664	1494.5
GSM1523822	alive	2.365556	72	F	I	0.029291	low	cluster_1	low	1304.662	3905.518
GSM1523823	alive	2.363333	25	F	III	0.010791	low	cluster_1	low	619.0193	2945.84
GSM1523824	alive	2.355556	55	M	II	0.012149	low	cluster_2	low	419.9035	1884.345
GSM1523825	dead	0.133333	64	F	III	0.073947	high	cluster_2	high	-217.982	-470.965
GSM1523826	dead	0.09	69	M	III	0.03579	high	cluster_2	low	761.1379	2912.619
GSM1523827	alive	2.334444	68	M	III	0.063342	high	cluster_2	low	1282.607	4217.287
GSM1523828	dead	0.195556	56	F	IV	0.074072	high	cluster_2	high	1247.984	2966.73
GSM1523829	alive	2.327778	76	M	II	0.071616	high	cluster_2	low	32.85506	1053.595
GSM1523830	dead	1.781111	64	M	II	-0.03494	low	cluster_1	low	107.9591	2247.981
GSM1523831	dead	1.03	81	F	IV	0.047022	high	cluster_2	high	-398.068	-1.14297
GSM1523832	dead	0.588889	62	F	III	0.043691	high	cluster_2	high	745.4779	2492.17
GSM1523833	dead	0.295556	72	M	IV	0.01977	low	cluster_2	low	-113.529	971.8
GSM1523834	dead	1.485556	78	M	III	0.054074	high	cluster_2	high	1851.802	4970.963
GSM1523835	alive	2.31	73	M	II	0.126552	high	cluster_2	high	1158.316	2059.979
GSM1523836	alive	2.307778	77	F	III	0.023085	low	cluster_1	low	1028.202	3534.337
GSM1523837	dead	0.894444	84	F	I	0.035632	high	cluster_2	low	-356.04	0.80281

GSM1523838	dead	0.344444	65	M	III	0.038137	high	cluster_1	low	912.206	3238.617
GSM1523839	alive	2.304444	62	M	III	0.028473	low	cluster_2	high	1297.172	3494.46
GSM1523840	dead	0.761111	38	F	II	0.013783	low	cluster_2	high	-285.905	758.1739
GSM1523841	dead	1.402222	77	M	II	0.01992	low	cluster_2	low	755.8485	2418.335
GSM1523842	dead	1.624444	28	F	II	0.057689	high	cluster_2	low	400.6395	2207.407
GSM1523843	dead	0.862222	65	M	IV	0.066725	high	cluster_2	high	1058.314	3020.295
GSM1523844	alive	2.296667	62	M	III	0.093424	high	cluster_2	high	1193.972	1956.258
GSM1523845	dead	0.322222	78	F	IV	0.059496	high	cluster_2	high	1292.927	3564.639
GSM1523846	alive	2.278889	76	M	III	0.06825	high	cluster_2	high	1517.751	3913.139
GSM1523847	alive	2.271111	53	M	II	-0.03674	low	cluster_1	low	161.7086	1722.845
GSM1523848	alive	2.271111	49	F	III	0.058711	high	cluster_2	low	2054.992	5657.606
GSM1523849	dead	0.934444	75	M	III	0.09811	high	cluster_2	high	1950.754	3974.911
GSM1523850	dead	1.213333	40	M	IV	0.111408	high	cluster_2	high	1556.702	3412.29
GSM1523851	alive	2.241111	67	M	I	0.009592	low	cluster_1	low	270.5222	2182.63
GSM1523852	dead	1.033333	49	M	III	0.021677	low	cluster_2	low	366.6066	2548.772
GSM1523853	alive	2.224444	69	M	I	0.021927	low	cluster_2	low	-20.8458	1117.864
GSM1523854	alive	2.224444	59	M	II	0.045911	high	cluster_2	high	964.2654	3266.043
GSM1523855	dead	0.731111	74	M	III	0.005749	low	cluster_2	high	-753.709	-39.2231
GSM1523856	alive	2.206667	49	F	II	0.05852	high	cluster_2	high	809.607	2217.811
GSM1523857	dead	0.481111	56	M	III	0.090469	high	cluster_2	high	1278.297	2531.893
GSM1523858	dead	0.57	63	F	I	0.21058	high	cluster_2	high	2031.259	3135.266
GSM1523859	alive	1.771111	60	M	II	0.016599	low	cluster_2	high	209.5213	1453.171
GSM1523860	dead	1.406667	57	M	I	0.005884	low	cluster_1	low	645.808	2761.723
GSM1523861	alive	2.16	65	M	I	0.028718	low	cluster_2	high	250.7461	2198.447
GSM1523862	dead	0.7	65	M	IV	0.161148	high	cluster_2	high	1703.858	3807.392
GSM1523863	alive	2.152222	43	M	I	0.005471	low	cluster_1	low	317.359	2916.673
GSM1523864	alive	2.132222	46	M	I	0.015762	low	cluster_1	low	-135.002	944.4968
GSM1523865	alive	2.123333	80	M	II	0.047716	high	cluster_2	low	568.0325	1744
GSM1523866	dead	0.145556	56	M	IV	0.078445	high	cluster_2	high	1918.666	4467.729
GSM1523867	dead	1.108889	66	M	III	0.010144	low	cluster_1	low	108.9016	1778.967
GSM1523868	alive	2.108889	29	M	III	0.030653	low	cluster_2	low	1137.477	4041.747
GSM1523869	alive	2.084444	65	M	II	0.031592	low	cluster_2	low	333.5231	2101.856
GSM1523870	alive	2.082222	56	M	II	0.007029	low	cluster_1	low	-118.61	1352.322
GSM1523871	alive	2.078889	53	M	II	0.019336	low	cluster_2	low	275.578	1813.465
GSM1523872	dead	0.501111	74	F	IV	0.070631	high	cluster_2	high	1569.206	4153.384
GSM1523873	dead	0.801111	62	M	IV	0.032846	low	cluster_2	low	641.136	3688.529
GSM1523874	alive	2.07	76	M	I	0.024685	low	cluster_2	low	940.4297	2606.543
GSM1523875	dead	0.282222	64	M	II	0.042902	high	cluster_2	high	-750.501	-516.704
GSM1523876	alive	2.063333	68	M	I	-0.02375	low	cluster_1	low	-167.959	1478.183
GSM1523877	dead	0.436667	88	F	III	0.008132	low	cluster_2	low	-768.009	-707.254
GSM1523878	alive	2.062222	47	F	II	0.020084	low	cluster_1	low	555.8343	3042.161
GSM1523879	alive	2.038889	82	M	IV	0.062752	high	cluster_2	low	-115.937	1268.948
GSM1523880	alive	2.032222	65	M	I	-0.01295	low	cluster_1	low	83.03027	2197.877

GSM1523881	dead	0.496667	49	F	III	0.032508	low	cluster_2	high	-419.965	430.9726
GSM1523882	alive	2.023333	58	F	III	0.022444	low	cluster_1	low	-718.948	992.4591
GSM1523883	dead	1.317778	92	M	II	0.015101	low	cluster_1	low	-242.374	1636.294
GSM1523884	alive	2.014444	68	M	III	0.0657	high	cluster_2	low	1106.693	3145.791
GSM1523885	alive	2.007778	76	M	I	-0.00064	low	cluster_1	low	132.8487	2444.58
GSM1523886	dead	0.186667	78	M	IV	0.043324	high	cluster_2	high	2119.712	4807.29
GSM1523887	alive	2.006667	50	F	I	0.009727	low	cluster_1	low	805.7378	2993.599
GSM1523888	alive	1.996667	59	M	I	0.009255	low	cluster_1	low	809.9565	3762.56
GSM1523889	alive	1.988889	67	F	I	0.01173	low	cluster_1	low	-916.816	-350.695
GSM1523890	alive	1.985556	71	M	III	0.023858	low	cluster_2	low	439.938	2269.79
GSM1523891	alive	1.985556	65	M	I	0.044759	high	cluster_2	high	1805.396	4221.089
GSM1523892	alive	1.982222	48	F	I	0.065226	high	cluster_2	low	753.38	2913.765
GSM1523893	dead	0.67	68	M	I	0.036626	high	cluster_2	low	-283.948	787.4076
GSM1523894	dead	1.192222	70	M	I	-0.02122	low	cluster_1	low	-258.755	554.8874
GSM1523895	alive	1.967778	81	M	I	0.022463	low	cluster_1	high	-648.86	348.9969
GSM1523896	dead	0.934444	53	F	II	0.070598	high	cluster_2	low	940.4167	3576.742
GSM1523897	alive	1.962222	76	F	I	-0.02129	low	cluster_1	high	-1006.78	-650.869
GSM1523898	alive	1.961111	84	F	I	0.014047	low	cluster_1	low	-245.813	756.2788
GSM1523899	alive	1.935556	73	F	II	0.021209	low	cluster_2	low	-453.365	776.3287
GSM1523901	alive	1.915556	69	F	I	0.01701	low	cluster_1	low	377.0784	2378.02
GSM1523903	dead	1.326667	75	M	III	0.058107	high	cluster_2	low	254.8732	795.8578
GSM1523904	alive	1.845556	66	F	IV	0.023226	low	cluster_2	low	496.1264	2396.774
GSM1523905	alive	1.843333	68	M	II	0.043251	high	cluster_2	low	333.0981	1860.042
GSM1523906	alive	1.843333	68	F	I	0.058245	high	cluster_2	low	1289.79	4381.861
GSM1523908	alive	1.835556	69	M	IV	0.022464	low	cluster_2	low	1026.638	3303.357
GSM1523909	alive	1.817778	64	M	II	0.009493	low	cluster_1	low	492.5389	2902.982
GSM1523910	alive	1.811111	63	M	III	-0.03666	low	cluster_1	low	-167.227	1847.659
GSM1523911	dead	0.727778	88	M	IV	-0.00314	low	cluster_1	low	-280.888	997.9352
GSM1523918	dead	0.937778	68	M	III	0.161446	high	cluster_2	high	1645.537	2811.68
GSM1523919	alive	2.803333	64	F	IV	0.091782	high	cluster_2	low	557.5854	2559.888
GSM1523920	dead	0.311111	79	M	II	0.046746	high	cluster_2	low	610.3981	2648.114
GSM1523921	alive	2.776667	67	F	II	0.03734	high	cluster_2	low	562.5388	2306.217
GSM1523922	dead	0.786667	85	F	II	0.038845	high	cluster_2	high	701.4611	2522.321
GSM1523923	dead	0.88	64	M	II	-0.01377	low	cluster_1	low	-76.6304	1332.887
GSM1523924	alive	2.74	58	M	II	0.074897	high	cluster_2	high	1028.519	2933.578
GSM1523925	dead	0.21	53	M	II	0.116623	high	cluster_2	high	35.12907	1283.389
GSM1523926	alive	2.301111	80	F	II	-0.01124	low	cluster_1	low	-941.6	-444.321
GSM1523927	alive	2.692222	76	M	II	0.077784	high	cluster_2	low	680.9593	2211.717
GSM1523928	dead	1.804444	71	F	II	0.047476	high	cluster_2	high	-120.879	860.5023
GSM1523929	alive	2.944444	71	M	III	-0.01331	low	cluster_1	low	183.1165	2348.188
GSM1523930	alive	2.617778	61	F	II	0.055638	high	cluster_2	low	795.6215	2074.59
GSM1523931	alive	2.586667	53	F	II	0.044584	high	cluster_2	low	684.1826	3918.097
GSM1523932	dead	0.422222	33	F	II	0.037264	high	cluster_2	low	-639.325	330.9814

GSM1523933	alive	2.508889	72	M	II	0.01818	low	cluster_2	high	110.7282	1841.176
GSM1523934	dead	0.426667	70	M	IV	0.033516	low	cluster_2	high	-108.532	577.6367
GSM1523935	alive	2.913333	32	F	II	0.086734	high	cluster_2	high	2361.552	4991.987
GSM1523936	dead	0.585556	71	F	II	0.067282	high	cluster_2	high	1233.719	3328.906
GSM1523937	dead	0.655556	65	M	II	0.032631	low	cluster_2	low	645.3408	3218.131
GSM1523938	dead	0.192222	56	M	IV	0.166226	high	cluster_2	high	2246.278	3835.122
GSM1523939	dead	0.374444	53	M	IV	0.162921	high	cluster_2	high	2239.382	4299.047
GSM1523940	dead	0.667778	68	M	IV	0.051532	high	cluster_2	high	-338.664	93.29866
GSM1523941	dead	0.151111	72	F	IV	0.037426	high	cluster_2	high	671.394	3125.57
GSM1523942	alive	2.342222	66	M	IIII	0.035046	high	cluster_2	low	31.26447	1285.806
GSM1523943	dead	0.184444	56	M	IV	0.041297	high	cluster_2	high	129.3324	1761.439
GSM1523944	alive	2.321111	39	F	III	0.030404	low	cluster_2	low	460.0329	3196.495
GSM1523945	dead	0.584444	47	F	IV	0.077147	high	cluster_2	high	1785.622	4077.882
GSM1523946	alive	2.297778	53	F	III	0.019731	low	cluster_2	high	-10.2119	1769.74
GSM1523947	alive	2.281111	61	M	II	-0.02934	low	cluster_1	low	-281.37	1255.48
GSM1523948	dead	0.915556	58	F	IV	0.089415	high	cluster_2	high	1388.482	3037.959
GSM1523949	dead	0.336667	57	F	IV	0.142863	high	cluster_2	high	1815.019	3931.651
GSM1523950	alive	2.273333	67	M	IV	-0.01333	low	cluster_1	low	-1398.11	-1029.9
GSM1523951	dead	1.767778	68	F	IV	0.064677	high	cluster_2	low	1077.71	4159.458
GSM1523952	alive	3.362222	42	F	II	0.032327	low	cluster_2	low	739.8436	3022.788
GSM1523953	alive	2.825556	69	M	III	0.03324	low	cluster_2	low	-1243.77	-1689.13
GSM1523954	alive	3.362222	68	M	II	0.043706	high	cluster_2	low	-1298	-1160.75
GSM1523955	dead	1.145556	56	M	III	0.089331	high	cluster_2	high	242.3001	1527.539
GSM1523956	dead	1.037778	60	M	II	0.038344	high	cluster_2	high	-42.1799	624.0745
GSM1523957	alive	3.354444	68	F	II	-0.00041	low	cluster_2	low	-802.932	-841.222
GSM1523958	alive	3.341111	52	F	II	0.031918	low	cluster_2	low	1104.783	3882.535
GSM1523959	dead	0.581111	52	M	IV	0.017959	low	cluster_2	low	-788.457	-277.124
GSM1523960	alive	3.3	71	F	II	0.052509	high	cluster_2	low	-391.46	-623.894
GSM1523961	dead	0.316667	63	M	II	0.061675	high	cluster_2	low	-550.162	-251.176
GSM1523962	dead	0.671111	62	F	III	0.155467	high	cluster_2	high	1859.7	3861.587
GSM1523963	dead	1.525556	58	F	II	0.04714	high	cluster_2	low	40.0655	1517.048
GSM1523964	alive	3.284444	75	F	II	0.000125	low	cluster_1	low	-347.836	263.0548
GSM1523965	dead	0.382222	56	M	IV	0.026109	low	cluster_2	high	1613.36	4240.877
GSM1523966	dead	1.726667	77	F	IV	0.054651	high	cluster_2	high	72.51233	1553.524
GSM1523967	alive	3.282222	48	M	III	0.022885	low	cluster_2	low	194.994	2371.053
GSM1523968	dead	1.505556	60	M	IV	0.157113	high	cluster_2	high	2029.981	3373.396
GSM1523969	dead	0.596667	56	F	III	0.158729	high	cluster_2	high	1170.192	2424.048
GSM1523970	dead	0.523333	77	F	II	0.021906	low	cluster_2	high	-605.217	-839.177
GSM1523971	dead	0.443333	84	F	III	0.123394	high	cluster_2	high	1468.049	3472.813
GSM1523972	dead	0.216667	37	F	IV	0.055409	high	cluster_2	high	-171.029	995.7875
GSM1523973	alive	2.908889	63	M	III	0.04751	high	cluster_2	high	298.7256	1625.801
GSM1523974	alive	3.274444	55	F	II	0.015197	low	cluster_2	low	-896.483	-507.994
GSM1523975	dead	0.24	44	F	III	0.134703	high	cluster_2	high	1992.27	5290.728

GSM1523976	dead	1.866667	60	F	III	0.028015	low	cluster_2	high	-226.124	627.8542
GSM1523977	dead	0.033333	44	F	II	0.096866	high	cluster_2	high	-344.287	-416.071
GSM1523978	dead	0.541111	57	F	IV	0.022006	low	cluster_1	low	-207.364	1468.342
GSM1523979	dead	0.314444	42	F	III	-0.00426	low	cluster_1	low	384.6787	2108.834
GSM1523980	dead	0.223333	69	M	III	0.071833	high	cluster_2	high	1480.279	4365.197
GSM1523981	alive	3.24	62	M	II	0.072037	high	cluster_2	high	2353.347	5113.443
GSM1523982	dead	0.29	60	M	IV	0.081275	high	cluster_2	high	2751.697	5877.579
GSM1523983	dead	0.336667	55	M	III	0.02879	low	cluster_2	high	497.4974	1946.855
GSM1523984	alive	3.232222	82	F	II	0.057543	high	cluster_2	high	56.48504	169.8139
GSM1523985	dead	2.584444	67	M	III	0.013432	low	cluster_2	low	731.0572	2830.533
GSM1523986	alive	2.893333	70	M	III	0.097794	high	cluster_2	high	1176.674	4078.251
GSM1523987	dead	0.608889	53	M	III	0.072244	high	cluster_2	low	323.2916	2487.563
GSM1523988	dead	0.494444	63	M	III	0.187429	high	cluster_2	high	2098.312	4173.157
GSM1523989	alive	2.89	84	M	IV	0.087906	high	cluster_2	high	1572.097	3802.395
GSM1523990	dead	0.472222	81	M	III	0.131942	high	cluster_2	high	1374.546	2211.272
GSM1523991	alive	3.173333	73	M	II	0.071512	high	cluster_2	high	746.9799	1475.834
GSM1523992	dead	0.671111	74	M	IV	0.091363	high	cluster_2	low	11.80863	632.7545
GSM1523993	dead	2.271111	70	F	III	0.025574	low	cluster_2	low	682.5043	3059.441
GSM1523994	alive	2.878889	72	F	III	0.088404	high	cluster_2	low	414.4439	2152.22
GSM1523995	dead	0.212222	37	F	III	0.011255	low	cluster_2	low	-248.771	184.0509
GSM1523996	dead	1.173333	72	F	II	0.055016	high	cluster_2	high	729.9502	2084.125
GSM1523997	dead	0.84	68	M	IV	0.003047	low	cluster_1	high	-98.7839	828.9007
GSM1523998	dead	0.707778	70	F	III	0.006848	low	cluster_1	low	-835.239	-545.148
GSM1523999	alive	3.128889	56	M	II	0.038785	high	cluster_2	high	-749.918	-606.583
GSM1524000	alive	3.128889	77	M	III	0.04597	high	cluster_2	high	1165.585	3340.891
GSM1524001	alive	3.121111	66	M	II	0.000504	low	cluster_1	high	-257.825	770.5393
GSM1524002	alive	2.871111	80	F	III	-0.0136	low	cluster_1	low	219.0429	3228.583
GSM1524003	alive	2.864444	62	M	III	0.043087	high	cluster_2	low	-817.918	-230.458
GSM1524004	dead	0.197778	70	M	IV	0.024081	low	cluster_2	low	170.1599	1991.573
GSM1524005	dead	0.315556	68	M	IV	0.013722	low	cluster_2	high	-340.755	701.6589
GSM1524006	dead	0.875556	64	M	III	0.163214	high	cluster_2	high	2543.417	4726.827
GSM1524007	dead	0.345556	66	M	IV	0.044816	high	cluster_2	high	584.0216	2397.143
GSM1524008	dead	0.361111	73	M	IV	-0.01223	low	cluster_1	high	-223.453	1265.524
GSM1524009	dead	2.166667	66	F	III	0.103587	high	cluster_2	high	1579.234	3464.721
GSM1524010	dead	0.656667	77	F	III	0.14524	high	cluster_2	low	1581.834	3945.94
GSM1524011	alive	3.006667	64	M	II	0.008628	low	cluster_2	low	-890.18	-602.71
GSM1524012	alive	2.841111	64	M	III	0.005496	low	cluster_2	high	-89.6136	1831.531
GSM1524013	alive	2.996667	66	M	II	0.037437	high	cluster_2	low	107.2448	1538.851
GSM1524014	dead	0.377778	64	F	IV	0.034635	low	cluster_2	high	-276.136	-40.0894
GSM1524015	dead	0.828889	64	M	IV	0.056639	high	cluster_2	high	-543.607	-193.652
GSM1524016	dead	0.926667	69	M	IV	0.016686	low	cluster_2	low	441.1874	2891.906
GSM1524017	alive	2.834444	74	M	III	0.037072	high	cluster_2	low	151.4862	1828.985
GSM1524018	alive	2.825556	36	F	III	0.024724	low	cluster_2	low	-317.625	1098.227

GSM1524019	dead	0.277778	72	M	IV	0.033577	low	cluster_2	low	617.3381	2788.246
GSM1524020	alive	2.81	68	M	III	0.026906	low	cluster_2	high	258.1828	1896.893
GSM1524021	dead	0.431111	59	M	III	0.053051	high	cluster_2	high	1051.771	3214.98
GSM1524022	dead	0.267778	68	M	IV	0.131612	high	cluster_2	high	1781.67	3997.686
GSM1524023	alive	2.794444	62	M	IV	0.028864	low	cluster_2	low	-84.2165	1053.513
GSM1524024	dead	2.981111	65	M	III	0.053746	high	cluster_2	high	758.4414	2048.152
GSM1524025	dead	2.727778	66	F	III	0.005984	low	cluster_1	high	832.6685	2875.056
GSM1524026	alive	2.786667	54	M	III	0.107037	high	cluster_2	high	1692.275	3450.641
GSM1524027	dead	1.99	56	M	III	0.034829	low	cluster_2	low	312.0913	1709.399
GSM1524028	dead	1.59	70	M	III	0.00881	low	cluster_1	low	-226.419	918.0664
GSM1524029	dead	1.036667	65	M	IV	0.164178	high	cluster_2	high	1600.467	3198.762
GSM1524030	dead	1.044444	57	M	III	0.172054	high	cluster_2	high	1938.67	3635.677
GSM1524031	alive	2.733333	78	M	IV	0.028552	low	cluster_2	high	-74.1444	950.5779
GSM1524032	dead	2.573333	65	M	IV	0.005301	low	cluster_2	high	756.4898	2244.652
GSM1524033	alive	2.694444	70	M	IV	0.015792	low	cluster_2	low	460.611	3061.853
GSM1524034	dead	1.468889	65	F	III	0.078289	high	cluster_2	low	685.3938	2912.999
GSM1524035	alive	2.676667	54	M	IV	0.109613	high	cluster_2	high	2092.606	4400.218
GSM1524036	alive	2.664444	77	F	III	0.095064	high	cluster_2	high	497.0216	1201.029
GSM1524037	dead	0.781111	63	M	III	0.122651	high	cluster_2	high	1516.22	3333.63
GSM1524038	dead	0.287778	31	F	IV	-0.02326	low	cluster_1	high	925.0966	3742.923
GSM1524039	dead	0.402222	68	M	III	0.041113	high	cluster_2	high	2073.957	4796.749
GSM1524040	dead	1.205556	65	M	IV	0.02156	low	cluster_2	low	471.0476	2820.283
GSM1524041	alive	2.577778	38	M	IV	0.002865	low	cluster_1	low	856.263	2533.239
GSM1524042	dead	0.361111	77	F	III	0.026314	low	cluster_2	high	604.0313	2927.603
GSM1524043	dead	0.561111	55	M	IV	0.018092	low	cluster_1	low	1010.849	3771.038
GSM1524044	dead	0.762222	68	M	IV	0.073684	high	cluster_2	high	1126.235	2760.293
GSM1524045	alive	2.971111	69	M	II	-0.00495	low	cluster_2	low	-493.324	-50.4006
GSM1524046	alive	2.934444	77	M	II	0.085731	high	cluster_2	high	1397.158	3514.385
GSM1524047	alive	2.328889	51	F	II	0.020412	low	cluster_1	high	0.702024	1619.489
GSM1524048	dead	1.56	75	F	II	0.07511	high	cluster_2	high	1471.473	3175.858
GSM1524049	alive	2.926667	42	F	II	0.091418	high	cluster_2	high	1481.473	3097.296
GSM1524050	dead	1.904444	67	M	II	0.043977	high	cluster_2	high	-478.457	-20.7901
GSM1524051	alive	2.908889	66	M	II	0.032798	low	cluster_2	low	626.6027	2972.962
GSM1524052	dead	0.438889	64	M	II	0.110138	high	cluster_2	high	945.7392	1674.094
GSM1524053	alive	2.897778	41	M	II	0.034446	low	cluster_2	high	504.9291	2083.9
GSM1524054	alive	2.885556	64	F	II	0.002169	low	cluster_2	low	-32.7113	901.1492
GSM1524055	dead	1.264444	52	M	II	0.064089	high	cluster_2	high	1041.42	2493.612
GSM1524056	dead	0.472222	70	M	II	0.040544	high	cluster_2	high	-179.632	1309.682
GSM1524057	dead	0.531111	86	F	IV	0.155962	high	cluster_2	high	1914.506	3120.092
GSM1524058	alive	2.848889	54	M	II	0.059494	high	cluster_2	high	1842.279	4991.705
GSM1524059	alive	2.84	62	M	II	0.057531	high	cluster_2	high	2076.584	5073.794
GSM1524060	alive	2.827778	67	F	II	0.013257	low	cluster_2	high	-529.999	56.64226
GSM1524061	alive	2.82	75	M	II	-0.02501	low	cluster_1	low	-832.526	-445.044

GSM1524062	dead	2.34	70	F	III	0.180585	high	cluster_2	high	1783.944	3463.769
GSM1524068	alive	3.095556	61	F	II	0.05237	high	cluster_2	low	-337.037	690.1393
GSM1524069	dead	2.853333	57	M	II	-0.00455	low	cluster_1	low	-727.043	147.1195
GSM1524070	alive	3.054444	72	F	II	0.062019	high	cluster_2	high	-376.248	303.5912
GSM1524071	alive	3.025556	79	M	III	0.011996	low	cluster_2	low	-122.526	910.0386
GSM1524072	dead	1.431111	76	M	IV	0.068405	high	cluster_2	high	523.2869	2223.051
GSM387789	alive	1.015556	65	M	IV	0.052175	high	cluster_2	high	1191.773	3617.641
GSM387791	alive	0.677778	56	M	IV	0.028591	low	cluster_2	low	291.1138	3206.582
GSM387792	dead	0.391111	63	M	IV	0.040342	high	cluster_2	low	326.3055	1317.485
GSM387794	dead	0.875556	54	F	IV	0.148839	high	cluster_2	high	1437.199	2952.613
GSM387795	alive	0.902222	74	M	III	0.141425	high	cluster_2	high	742.484	1872.197
GSM387796	dead	0.683333	36	M	III	-0.00395	low	cluster_1	low	-1148.69	-215.224
GSM387800	alive	0.233333	77	M	III	0.005105	low	cluster_1	low	-323.202	679.3957
GSM387801	alive	0.671111	50	M	I	0.036188	high	cluster_2	low	-665.141	3.413689
GSM387802	alive	0.506667	53	F	I	0.000341	low	cluster_1	low	-242.516	1955.102
GSM387803	alive	1.09	49	M	IV	-0.03879	low	cluster_1	low	-1637.36	-1074.24
GSM387804	alive	0.422222	46	M	III	0.128728	high	cluster_2	high	1015.248	2619.889
GSM387805	alive	0.363333	52	M	I	-0.01763	low	cluster_1	low	-1222.38	-13.2121
GSM387806	alive	0.657778	74	M	III	0.002315	low	cluster_1	low	554.4629	3180.793
GSM387807	alive	0.164444	33	F	II	0.010989	low	cluster_1	high	-244.281	984.8989
GSM387808	alive	0.195556	53	F	IV	0.050443	high	cluster_2	low	-367.448	83.24081
GSM387809	alive	0.156667	59	M	III	-0.02609	low	cluster_1	high	-278.475	145.6438
GSM387810	alive	0.123333	74	M	I	0.006635	low	cluster_1	low	-123.856	1936.271
GSM387811	dead	0.107778	56	M	IV	0.003073	low	cluster_1	high	123.2622	1189.855
GSM387812	alive	0.37	66	M	III	0.013214	low	cluster_1	low	788.3371	2538.191
GSM387813	alive	0.131111	51	M	I	-0.06801	low	cluster_1	low	-603.177	105.1
GSM387814	dead	0.573333	78	F	IV	0.075032	high	cluster_2	low	1659.535	4525.976
GSM387815	alive	2.097778	60	M	II	0.025269	low	cluster_2	low	-442.555	772.6036
GSM387816	dead	0.055556	60	F	IV	0.055508	high	cluster_2	high	1007.719	3468.756
GSM387817	alive	2.935556	65	F	I	0.018049	low	cluster_2	low	-246.927	660.3799
GSM387818	alive	3.623333	60	M	II	0.005078	low	cluster_2	high	-4.60951	611.7303
GSM387819	dead	0.972222	56	M	IV	0.086287	high	cluster_2	high	1911.818	4095.302
GSM387820	alive	4.33	66	M	IV	-0.00314	low	cluster_1	low	74.40136	1715.689
GSM387821	alive	4.098889	53	M	I	0.055016	high	cluster_2	low	488.3992	1876.071
GSM387823	dead	0.22	44	F	IV	0.086596	high	cluster_2	high	963.4108	2450.108
GSM387824	alive	4.216667	74	M	II	0.117414	high	cluster_2	high	1519.118	3733.919
GSM387825	alive	4.183333	53	M	I	0.072365	high	cluster_2	low	822.5857	2676.861
GSM387826	alive	0.368889	72	F	II	0.11338	high	cluster_2	high	1209.711	3018.032
GSM387827	alive	3.106667	62	M	I	0.083153	high	cluster_2	low	878.7256	2258.904
GSM387828	alive	3.925556	64	M	III	0.004131	low	cluster_1	low	280.6439	2325.855
GSM387829	alive	4.248889	44	M	II	0.063785	high	cluster_2	low	808.2778	3039.207
GSM387830	dead	2.9	42	M	III	0.038804	high	cluster_2	high	923.3539	2993.358
GSM387831	dead	0.345556	65	M	IV	-0.01685	low	cluster_1	low	-478.389	927.3416

GSM387832	alive	4.184444	59	M	III	0.117851	high	cluster_2	high	1744.459	3927.818
GSM387833	dead	0.284444	52	M	III	0.098634	high	cluster_2	high	420.2351	1260.125
GSM387834	dead	1.398889	59	F	II	0.093816	high	cluster_2	high	1465.52	3029.685
GSM387835	dead	0.435556	69	M	III	-0.00127	low	cluster_1	low	1213.168	4307.587
GSM387836	dead	0.658889	46	M	III	0.063874	high	cluster_2	high	680.0076	1963.693
GSM387837	dead	1.25	69	M	III	0.076177	high	cluster_2	high	1029.286	2312.93
GSM387838	dead	1.013333	67	F	III	0.09996	high	cluster_2	high	1836.568	4013.054
GSM387840	alive	4.308889	51	M	I	0.145935	high	cluster_2	high	1040.185	2465.042
GSM387841	dead	0.138889	61	F	IV	0.117921	high	cluster_2	high	2099.536	4863.476
GSM387842	dead	0.571111	66	F	IV	0.073441	high	cluster_2	high	449.7835	2056.225
GSM387843	dead	0.075556	74	M	IV	0.083636	high	cluster_2	high	-110.867	528.9437
GSM387845	dead	0.137778	70	M	IV	0.074667	high	cluster_2	high	870.2497	2569.776
GSM387846	dead	3.143333	64	M	III	0.017317	low	cluster_1	high	575.7756	2269.538
GSM387847	dead	3.684444	70	M	I	0.114601	high	cluster_2	high	1725.346	3657.483
GSM387848	dead	3.314444	81	M	III	0.017626	low	cluster_1	low	1104.183	3714.044
GSM387849	dead	0.38	53	F	IV	0.06598	high	cluster_2	high	1586.995	3589.516
GSM387850	dead	0.215556	28	F	IV	0.114679	high	cluster_2	high	1977.14	4706.342
GSM387851	dead	0.362222	69	M	IV	0.09968	high	cluster_2	high	1327.842	3260.461
GSM387852	dead	0.566667	70	M	IV	0.0935	high	cluster_2	high	1500.099	3006.661
GSM387853	dead	0.553333	70	M	IV	0.144108	high	cluster_2	high	1054.901	2718.525
GSM387855	alive	3.821111	72	F	II	0.017695	low	cluster_2	high	-653.982	-744.377
GSM387856	dead	0.872222	71	M	III	0.021343	low	cluster_1	high	1421.552	3626.576
GSM387857	dead	0.268889	53	M	III	0.100011	high	cluster_2	high	1688.728	3998.868
GSM387858	dead	0.308889	50	M	IV	0.10588	high	cluster_2	high	1438.189	3419.638
GSM387859	dead	0.368889	74	F	III	0.066214	high	cluster_2	high	2089.793	5240.173
GSM387861	dead	0.773333	52	M	III	0.035448	high	cluster_2	low	235.787	1584.975
GSM387862	dead	0.156667	51	F	IV	0.085893	high	cluster_2	high	1749.118	4057.716
GSM387863	alive	1.502222	57	M	I	0.091452	high	cluster_2	high	1892.078	4165.773
GSM387864	dead	0.143333	66	M	III	0.056609	high	cluster_2	high	857.7883	2586.571
GSM387865	dead	0.812222	54	F	IV	0.086901	high	cluster_2	high	805.3429	2100.985
GSM387866	dead	0.445556	70	M	III	0.104148	high	cluster_2	high	1878.533	4280.427
GSM387867	dead	0.76	62	M	II	0.028824	low	cluster_2	low	299.2515	2007.197
GSM387868	alive	2.218889	49	M	II	0.050473	high	cluster_2	high	2106.275	4930.612
GSM387869	dead	0.695556	76	M	III	0.091839	high	cluster_2	high	1030.547	2943.274
GSM387870	dead	0.153333	37	F	IV	0.069686	high	cluster_2	high	977.1295	2823.285
GSM387871	dead	0.434444	56	M	IV	-0.00511	low	cluster_1	low	45.77156	1490.702
GSM387872	alive	2.854444	54	M	I	0.067734	high	cluster_2	high	628.3735	1996.62
GSM387873	alive	3.27	70	F	III	0.0369	high	cluster_2	low	168.4654	2407.447
GSM387874	dead	0.477778	63	M	IV	0.027554	low	cluster_2	high	1260.162	3091.778
GSM387875	alive	2.956667	48	M	I	0.083181	high	cluster_2	high	1216.538	2915.467
GSM387876	dead	0.838889	69	F	II	0.112747	high	cluster_2	high	1832.463	4551.896
GSM387877	alive	2.416667	61	M	III	0.017145	low	cluster_1	low	1304.711	4414.93
GSM387878	alive	0.784444	52	M	II	0.067609	high	cluster_2	high	936.7584	2273.295

GSM387879	dead	1.04	35	M	I	0.005673	low	cluster_1	low	-93.8829	1452.201
GSM387880	alive	2.498889	52	M	I	0.09857	high	cluster_2	high	1510.275	3424.593
GSM387881	dead	0.408889	44	F	IV	0.097236	high	cluster_2	high	962.7758	1973.153
GSM387882	dead	0.545556	69	M	III	0.027902	low	cluster_2	low	337.1081	1920.272
GSM387883	alive	1.416667	77	F	II	0.01247	low	cluster_1	low	263.9592	2281.671
GSM387884	dead	0.328889	45	F	II	0.021208	low	cluster_2	low	-26.1103	1709.25
GSM387885	alive	1.281111	70	M	III	0.05805	high	cluster_2	low	180.1157	2951.608
GSM387886	alive	2.425556	62	M	II	0.007344	low	cluster_1	low	698.0867	2960.149
GSM387887	dead	0.194444	61	M	IV	0.053806	high	cluster_2	low	1174.835	3861.749
GSM387888	alive	1.19	67	M	I	0.089129	high	cluster_2	high	1135.374	2240.059
GSM387889	dead	0.368889	69	M	IV	0.036915	high	cluster_2	high	839.4701	2687.013
GSM387891	alive	2.152222	63	M	I	0.045204	high	cluster_2	low	205.3407	2196.772
GSM387892	dead	0.265556	67	F	IV	0.041272	high	cluster_2	high	827.2628	1566.968
GSM387893	alive	1.765556	78	M	III	0.095947	high	cluster_2	high	922.3619	2505.278
GSM387894	alive	2.218889	77	F	I	-0.04289	low	cluster_1	low	-634.934	760.9339
GSM387895	alive	0.087778	51	M	III	0.00432	low	cluster_1	low	511.2325	2521.131
GSM387896	dead	0.332222	56	M	III	0.029232	low	cluster_2	high	266.5124	1140.775
GSM387897	alive	0.81	61	M	IV	0.053507	high	cluster_2	low	184.9989	2146.86
GSM387898	dead	2.62	57	M	II	0.10474	high	cluster_2	high	1384.354	3200.854
GSM387899	dead	0.392222	70	M	III	0.102557	high	cluster_2	high	1492.037	2906.042
GSM387900	alive	0.198889	72	M	I	0.013629	low	cluster_2	low	-306.507	1383.192
GSM387901	alive	2.762222	30	F	I	-0.03724	low	cluster_1	low	-1182.07	-1189.97
GSM387902	dead	1.985556	65	M	II	0.080913	high	cluster_2	low	570.3185	3111.845
GSM387903	dead	1.374444	46	M	III	0.037195	high	cluster_2	low	-1334.33	-1816.01
GSM387904	alive	2.477778	40	M	II	0.026283	low	cluster_2	low	-228.179	610.5286
GSM387905	dead	0.208889	63	F	IV	0.079658	high	cluster_2	high	1767.655	4199.126
GSM387906	alive	0.433333	70	F	III	0.043502	high	cluster_2	high	1107.187	2522.2
GSM387907	alive	2.117778	69	M	III	0.031619	low	cluster_2	high	-255.544	963.0678
GSM387908	dead	0.608889	63	M	III	0.056102	high	cluster_2	low	823.2405	1997.619
GSM387909	dead	0.961111	82	M	IV	0.10106	high	cluster_2	high	1205.868	2753.093
GSM387910	dead	0.742222	75	F	IV	0.036554	high	cluster_2	high	365.523	1685.758
GSM387911	dead	0.83	72	F	IV	0.09221	high	cluster_2	high	1582.858	4284.182
GSM387912	alive	0.57	59	M	III	0.048027	high	cluster_2	high	359.2286	1856.278
GSM387913	dead	0.234444	66	M	IV	0.000211	low	cluster_1	low	-727.277	74.15952
GSM387914	dead	0.22	69	F	III	0.073722	high	cluster_2	high	476.7254	2213.832
GSM387915	alive	5.261111	58	M	II	0.070695	high	cluster_2	high	1164.918	3531.095
GSM387916	dead	2.123333	64	M	I	0.028614	low	cluster_2	low	-592.053	360.9152
GSM387917	alive	0.378889	60	F	IV	-0.01551	low	cluster_1	low	-290.182	636.0771
GSM387918	alive	5.167778	58	M	I	-0.00276	low	cluster_1	low	69.9513	1323.239
GSM387919	alive	0.347778	71	M	IV	0.055091	high	cluster_2	high	1036.227	3327.143
GSM387920	alive	0.735556	41	F	III	0.018551	low	cluster_2	high	-328.342	534.8402
GSM387922	alive	5.13	48	F	I	-0.03383	low	cluster_1	low	-313.855	1081.816
GSM387924	alive	0.161111	39	M	IV	0.037579	high	cluster_2	low	501.8388	2277.014

GSM387925	dead	4.121111	60	F	II	0.100218	high	cluster_2	high	1346.096	3444.045
GSM387926	alive	0.107778	73	F	IV	0.005967	low	cluster_1	low	-221.526	916.2763
GSM387927	dead	1.313333	63	F	IV	0.017029	low	cluster_1	high	898.6432	2998.227
GSM387928	alive	0.905556	64	M	II	-0.02488	low	cluster_1	low	288.0411	3044.688
GSM387929	dead	0.384444	63	M	IV	0.080991	high	cluster_2	high	1431.143	3076.607
GSM387930	dead	0.448889	66	F	III	-0.04979	low	cluster_1	low	-9.2163	2136.695
GSM387931	alive	4.963333	61	M	I	0.110074	high	cluster_2	high	1889.076	4170.741
GSM387932	dead	0.884444	24	M	IV	-0.02247	low	cluster_1	low	-603.54	235.1518
GSM387933	alive	0.433333	67	M	IV	0.066805	high	cluster_2	low	1160.354	3126.049
GSM387934	alive	4.725556	64	M	III	0.035847	high	cluster_2	low	825.0338	3453.95
GSM387935	dead	0.168889	70	M	IV	0.021251	low	cluster_2	low	-107.782	1512.854
GSM387936	dead	1.087778	51	M	III	0.098435	high	cluster_2	high	2075.395	4159.262
GSM387938	dead	0.213333	56	F	IV	0.035853	high	cluster_2	high	87.06366	1346.844
GSM387939	alive	4.871111	65	F	I	0.000709	low	cluster_1	low	-693.929	856.5576
GSM387940	dead	0.109	77	F	III	0.075273	high	cluster_2	high	1172.508	3334.698
GSM387941	dead	0.655556	61	M	I	-0.00289	low	cluster_1	low	81.77299	2183.752
GSM387943	alive	0.232222	71	M	IV	0.091507	high	cluster_2	high	579.5326	1990.386
GSM387944	alive	2.854444	52	F	III	-0.00657	low	cluster_1	low	-392.082	1322.14
GSM387945	alive	0.22	61	M	III	0.034016	low	cluster_2	high	884.6192	2311.69
GSM387946	dead	0.486667	72	M	II	0.052329	high	cluster_2	high	1007.282	3683.775
GSM387947	dead	0.596667	50	F	IV	0.050118	high	cluster_2	high	1218.329	3594.056
GSM387948	alive	4.582222	46	F	III	0.004633	low	cluster_1	low	395.9832	2067.169
GSM387949	alive	0.086667	55	M	III	-0.01998	low	cluster_1	low	-820.211	240.7263
GSM387950	dead	0.421111	70	F	III	0.095921	high	cluster_2	high	1067.014	3771.458
GSM387951	alive	0.052222	67	F	IV	0.002143	low	cluster_1	low	-976.654	-55.1179
GSM387952	alive	4.454444	65	M	III	-0.04776	low	cluster_1	low	-183.852	1077.937
GSM387953	alive	0.286667	64	F	IV	0.047055	high	cluster_2	high	1137.802	3992.298
GSM387954	dead	0.713333	70	M	III	0.085528	high	cluster_2	high	1220.78	2958.767
GSM387955	dead	0.432222	72	M	III	0.062049	high	cluster_2	high	375.1982	1302.164
GSM387956	alive	0.428889	74	M	III	0.08808	high	cluster_2	low	706.2274	2213.013
GSM387957	dead	0.508889	72	M	III	0.036373	high	cluster_2	high	575.2749	1876.428
GSM387958	alive	2.69	55	F	I	0.000962	low	cluster_2	low	-1832.15	-1977.7
GSM387959	dead	0.108889	56	M	IV	0.027347	low	cluster_2	low	1026.436	3997.281
GSM387960	dead	1.754444	62	F	III	0.135401	high	cluster_2	high	1477.446	2525.474
GSM387961	dead	0.675556	60	M	IV	0.051845	high	cluster_2	low	1052.852	3322.599
GSM387962	dead	0.3	80	M	III	0.049193	high	cluster_2	high	1144.676	3181.799
GSM387964	dead	0.352222	33	M	IV	0.015966	low	cluster_1	low	-137.027	1481.028
GSM387965	alive	1.964444	59	F	II	0.017172	low	cluster_1	low	597.5991	2642.047
GSM387966	alive	1	74	M	I	-0.05566	low	cluster_1	low	-1339.69	-1321.59
GSM387967	alive	4.612222	67	M	III	0.029462	low	cluster_2	high	1113.576	3415.374
GSM387968	dead	0.511111	62	M	III	0.015025	low	cluster_1	low	161.9484	1215.688
GSM387969	alive	3.774444	66	M	I	0.047456	high	cluster_2	low	879.2814	4527.98
GSM387970	dead	0.104444	51	F	III	0.039328	high	cluster_2	high	908.0536	2430.613

GSM387971	dead	0.785556	74	M	III	-0.02191	low	cluster_1	high	17.62836	1814.692
GSM387973	alive	0.244444	61	M	III	0.046798	high	cluster_2	low	605.5003	2184.535
GSM387974	alive	4.47	72	M	II	-0.01359	low	cluster_1	low	549.706	3411.896
GSM387975	dead	0.494444	54	M	III	0.042433	high	cluster_2	low	364.8517	1964.71
GSM387976	alive	4.127778	71	M	II	0.038469	high	cluster_2	high	592.4066	1941.77
GSM387977	dead	1.526667	80	M	III	0.038429	high	cluster_2	low	445.9227	2459.62
GSM387978	dead	1.332222	56	F	II	0.095337	high	cluster_2	high	1154.603	2713.759
GSM387979	dead	0.221111	58	M	IV	0.061103	high	cluster_2	high	299.0676	1353.386
GSM387980	dead	0.145556	56	M	III	0.072542	high	cluster_2	high	644.5589	1484.044
GSM387981	dead	0.821111	77	M	III	0.031304	low	cluster_2	low	249.373	1092.949
GSM387982	alive	2.517778	40	F	II	0.067423	high	cluster_2	low	131.289	1887.316
GSM387983	dead	1.896667	71	F	III	0.092541	high	cluster_2	high	1334.225	3106.929
GSM387984	alive	0.074444	63	M	III	0.003707	low	cluster_2	high	-538.318	-227.507
GSM387985	alive	3.886667	58	M	II	0.007004	low	cluster_1	low	50.34614	1074.866
GSM387986	alive	0.048889	60	M	I	0.033034	low	cluster_2	high	-482.784	44.12074
GSM387987	dead	0.257778	43	F	IV	0.071037	high	cluster_2	low	-389.267	-130.693

Table.5 Clinical information and treatment information for patients in IMvigor210.

id	OS	censOS	Sex	Best Confirmed Overall Response	Binary Response	HP_score
SAM00b9e5c52da9	1.905544	1	M	NE	NA	low
SAM0257bbbbd388	15.6386	1	M	SD	SD/PD	high
SAM025b45c27e05	8.772074	1	M	PD	SD/PD	high
SAM032c642382a7	2.49692	1	F	PD	SD/PD	low
SAM04c589eb3fb3	0.689938	0	M	NE	NA	low
SAM0571f17f4045	4.533881	1	F	SD	SD/PD	low
SAM065890737112	20.07392	0	M	CR	CR/PR	low
SAM0684af734db1	23.26078	0	F	PR	CR/PR	high
SAM075e037d95bc	21.32238	0	M	PR	CR/PR	high
SAM07a93a28f801	12.846	0	M	PR	CR/PR	high
SAM08cce2fa88f2	22.11088	0	F	SD	SD/PD	high
SAM09c84ec0cf34	6.505133	1	M	PD	SD/PD	high
SAM0a0f2bac4b20	1.182752	1	M	PD	SD/PD	low
SAM0a7c2091dd56	2.628337	1	M	PD	SD/PD	high
SAM0bdb3428bd13	2.234086	1	F	PD	SD/PD	high
SAM0ce9c983b20f	6.603696	1	M	PD	SD/PD	low
SAM0d855cff64e6	2.858316	1	F	PD	SD/PD	high
SAM0f956e757453	10.41478	1	M	PD	SD/PD	high
SAM110501d0eedb	4.271047	1	M	PD	SD/PD	high
SAM12502d970c10	7.392197	1	M	SD	SD/PD	high
SAM14938611a2d3	21.42094	0	M	PR	CR/PR	high
SAM14df63a65411	21.42094	0	F	PR	CR/PR	high
SAM166a419a4e5a	5.38809	1	M	PD	SD/PD	high
SAM17c45bf16bb6	2.694045	1	M	NE	NA	high
SAM18039827e1b9	1.478439	1	M	PD	SD/PD	high
SAM181b638b8248	10.87474	1	M	PD	SD/PD	low
SAM187e056d6a2a	2.299795	1	M	PD	SD/PD	high
SAM18a4dabbc557	16.45996	1	M	PD	SD/PD	low
SAM18b9351e265a	15.86858	1	M	PD	SD/PD	low
SAM18bc1078bc15	6.899384	1	M	PD	SD/PD	low
SAM18be5b395318	9.002053	1	M	PD	SD/PD	low
SAM19fec8f3b3bd	9.889117	1	M	PD	SD/PD	high
SAM1a87df750b9d	5.38809	1	M	SD	SD/PD	low
SAM1ab1b28d9f2b	2.102669	1	F	PD	SD/PD	high

SAM1abf01dd4544	16.85421	0	M	PR	CR/PR	high
SAM1ac4e3dee297	5.519507	1	M	PD	SD/PD	high
SAM1bcc62d8290c	18.62834	0	M	NE	NA	high
SAM1c0ecfb3eb63	5.848049	1	F	PD	SD/PD	low
SAM1c8b086175ca	20.76386	1	M	PD	SD/PD	high
SAM1dda30f1c5be	21.25667	0	M	PD	SD/PD	high
SAM1e9c4d1d39ae	12.41889	1	F	PD	SD/PD	low
SAM1f3c93814cb9	1.445585	1	F	NE	NA	high
SAM1f66db567eb5	16.59138	0	M	PR	CR/PR	low
SAM1f83ebd6be9b	3.5154	1	M	PD	SD/PD	high
SAM1fa6bcb7fc48	22.73511	0	M	PD	SD/PD	high
SAM203dcf14f927	19.35113	0	M	CR	CR/PR	low
SAM2070b416069c	8.016427	1	M	SD	SD/PD	high
SAM23095936e611	2.234086	1	F	PD	SD/PD	high
SAM23aa15d4a0b0	5.716632	1	F	PD	SD/PD	high
SAM25510f300d79	20.69815	0	M	CR	CR/PR	high
SAM2570ff4aae6e	22.37372	0	F	PR	CR/PR	low
SAM26104d5adc89	5.38809	1	M	SD	SD/PD	high
SAM2624229effe8	2.562628	1	M	PD	SD/PD	high
SAM27299aed7681	18.85832	0	F	PR	CR/PR	high
SAM28687037e4ff	21.58522	0	M	SD	SD/PD	low
SAM28e6031ac18b	9.232033	1	M	SD	SD/PD	high
SAM297c0301e861	8.082136	1	M	PD	SD/PD	high
SAM29da928587ad	2.595483	1	F	PD	SD/PD	high
SAM2b672f4336c7	15.31006	1	M	SD	SD/PD	high
SAM2bba8cb35e48	10.48049	1	M	PD	SD/PD	high
SAM2c9586161ce6	11.26899	1	M	PD	SD/PD	high
SAM2dc3f04e45e9	10.1848	1	M	PD	SD/PD	low
SAM2dc578e0165f	1.971253	1	M	NE	NA	high
SAM2de7cffb5f72	1.839836	1	M	NE	NA	high
SAM2e7aa8fa0ab3	7.720739	1	M	PD	SD/PD	high
SAM2e9ac0b1b250	22.14374	0	M	CR	CR/PR	low
SAM2eb07dedf07f	20.50103	0	M	PR	CR/PR	low
SAM2f228939632f	7.88501	1	F	PD	SD/PD	high
SAM30b5c6c54cf7	9.264887	1	F	CR	CR/PR	low
SAM30cf07d4874f	11.66324	1	M	SD	SD/PD	high
SAM31291c256373	1.149897	1	M	NE	NA	low
SAM31d9176e11fb	17.11704	0	M	CR	CR/PR	low
SAM31f41dd0d6ca	8.279261	1	F	SD	SD/PD	low
SAM3330c03fdf00	19.18686	0	M	PD	SD/PD	low
SAM34430ef08e5b	18.16838	0	M	PD	SD/PD	high
SAM36851bc8b9ae	3.548255	1	M	SD	SD/PD	high

SAM36a9225b0222	23.8193	0	M	SD	SD/PD	low
SAM36d87392593b	23.68789	0	M	SD	SD/PD	high
SAM3779e979db6b	1.314168	1	M	NE	NA	high
SAM3785587846ce	7.063655	1	F	NE	NA	high
SAM3894ac3956a5	2.102669	0	M	PD	SD/PD	high
SAM39eb94fa504d	17.90554	1	M	PD	SD/PD	high
SAM3a1c9632ff7b	16.03285	0	M	SD	SD/PD	high
SAM3b1066e5801b	15.6386	0	M	SD	SD/PD	high
SAM3b15b4c6311d	21.4538	0	M	CR	CR/PR	low
SAM3cb94b0d5297	5.059548	1	M	PD	SD/PD	high
SAM3e04eb914f3d	17.28131	0	M	PR	CR/PR	low
SAM3e8baff50d7a	6.965092	1	M	SD	SD/PD	high
SAM3ee5dcd894f0	8.706366	1	M	PD	SD/PD	high
SAM3f2033c90438	0.821355	1	M	NE	NA	high
SAM3f446449bf81	0.755647	1	M	NE	NA	low
SAM415f36ad349e	17.97125	1	M	PD	SD/PD	high
SAM4305ab968b90	1.412731	1	M	PD	SD/PD	high
SAM4501e41e4751	1.117043	1	M	PD	SD/PD	low
SAM4581bac493af	20.04107	0	M	NE	NA	low
SAM45c8e6412c66	3.482546	1	M	PD	SD/PD	low
SAM468a9e1dc821	3.876797	1	M	PD	SD/PD	high
SAM47fc46c3d6be	18.7269	0	M	SD	SD/PD	low
SAM4918c524b83a	0.62423	0	M	NE	NA	high
SAM491e341d5a82	1.87269	1	F	NE	NA	high
SAM49d48750e294	9.856263	1	M	PD	SD/PD	high
SAM49f9b2e57aa5	7.457906	1	M	PD	SD/PD	high
SAM4b0175e8db6e	2.069815	1	M	PD	SD/PD	high
SAM4b7ea015fd9e	13.2731	1	F	PD	SD/PD	high
SAM4caabd64e7fd	15.70431	0	M	PR	CR/PR	high
SAM4edbe45817b3	1.051335	1	M	NE	NA	low
SAM5234688806a7	5.880903	1	M	PD	SD/PD	high
SAM52500cabdd36	20.46817	0	F	SD	SD/PD	high
SAM52e3fa3ad574	7.950719	1	M	PD	SD/PD	low
SAM548551ef782c	17.28131	0	M	PR	CR/PR	high
SAM54e58f1b0230	4.36961	1	M	PD	SD/PD	high
SAM553c3c35b847	6.702259	1	F	PD	SD/PD	low
SAM557dde1b9f3e	6.702259	1	M	PD	SD/PD	high
SAM560f23d6a3ad	21.38809	0	M	CR	CR/PR	low
SAM563d6233dfa2	2.135524	1	M	PD	SD/PD	high
SAM568ce160abd9	12.846	1	F	SD	SD/PD	high
SAM572f19794c96	16.82136	0	M	CR	CR/PR	low
SAM5767dd75d142	18.16838	0	M	PR	CR/PR	low

SAM58e7832f4e7d	16.45996	0	M	PR	CR/PR	high
SAM59289ca42c99	1.708419	1	M	NE	NA	low
SAM59b825252c0d	0.525667	1	M	NE	NA	low
SAM59f392864f5d	17.77413	1	M	SD	SD/PD	low
SAM59fda9035d1d	3.74538	1	M	PD	SD/PD	high
SAM5a2347c0498a	4.895277	1	M	SD	SD/PD	high
SAM5b57e47fdcb3	3.843943	1	F	PD	SD/PD	high
SAM5c139c5c1c4f	7.227926	1	M	PD	SD/PD	high
SAM5cc2d9036053	5.683778	1	M	PD	SD/PD	high
SAM5cfa1699bdb7	15.83573	0	M	PD	SD/PD	high
SAM5d1dfd5207f5	10.9076	1	M	PD	SD/PD	high
SAM5d989c86255e	3.449692	1	F	PD	SD/PD	high
SAM5e3bae090b8c	6.735113	1	M	PD	SD/PD	low
SAM5fc9ae0aed1f	0.62423	1	M	NE	NA	low
SAM5fe7a81a39dd	17.08419	1	M	PD	SD/PD	high
SAM5ffd7e4cd794	1.215606	1	M	NE	NA	low
SAM6083aac8db99	20.63244	0	M	PR	CR/PR	low
SAM6157c8f38b72	16.59138	0	M	PR	CR/PR	high
SAM61b9d4d84c64	21.38809	0	M	SD	SD/PD	high
SAM61baf919bb01	15.60575	1	F	SD	SD/PD	low
SAM62fb1388c871	22.50513	0	F	PD	SD/PD	high
SAM63405b04ab2d	6.800821	1	M	PD	SD/PD	high
SAM63b2189c36d7	3.975359	1	M	PD	SD/PD	high
SAM65afda25b920	8.082136	1	M	SD	SD/PD	low
SAM6662f5181f87	19.28542	0	M	PD	SD/PD	low
SAM670649e105b5	22.07803	0	F	CR	CR/PR	high
SAM675a12a09c15	2.89117	1	M	PD	SD/PD	high
SAM6780ed436b55	6.2423	1	F	PD	SD/PD	high
SAM6792d6e98068	6.01232	1	M	PD	SD/PD	high
SAM681e4bf7cf85	23.3922	0	M	PR	CR/PR	high
SAM6964a6d7b967	16.26283	1	M	PD	SD/PD	high
SAM698d8d76b934	0.427105	1	F	NE	NA	high
SAM6cb230f208a8	23.22793	0	M	PR	CR/PR	high
SAM6cbc10abddb0	5.946612	1	M	PD	SD/PD	low
SAM6d2ae0c39b96	22.11088	0	M	PD	SD/PD	high
SAM6dd7ad1d797d	19.28542	1	M	PD	SD/PD	high
SAM6f2a102a99df	19.28542	0	F	CR	CR/PR	low
SAM6ff654a20f98	18.56263	0	M	CR	CR/PR	high
SAM7114d99032ec	10.84189	1	M	PD	SD/PD	high
SAM714285adf612	0.62423	1	M	NE	NA	low
SAM716f54e468f4	21.19097	0	M	SD	SD/PD	high
SAM727c0e92a2a7	18.7269	0	M	PR	CR/PR	low

SAM73663ee4a96e	21.25667	0	M	CR	CR/PR	low
SAM73b653ae20d1	1.806982	1	F	NE	NA	high
SAM75142fcab9df	11.92608	1	F	PD	SD/PD	low
SAM7538ad9ff524	19.12115	0	F	SD	SD/PD	low
SAM753d4bb52dbe	11.36756	1	M	SD	SD/PD	high
SAM75f12d1a55fc	14.75154	0	M	SD	SD/PD	low
SAM76a431ba6ce1	14.12731	1	M	PD	SD/PD	low
SAM771445e92421	19.41684	0	M	CR	CR/PR	low
SAM7746b76437e6	2.825462	1	M	NE	NA	high
SAM7829a341b9f3	3.876797	1	M	PD	SD/PD	high
SAM7893196e0e89	2.661191	1	M	PD	SD/PD	high
SAM7a9093b9c7e9	1.806982	1	M	NE	NA	high
SAM7aa01fc49a80	2.135524	1	M	PD	SD/PD	high
SAM7b40007f4aa4	21.65092	0	F	PR	CR/PR	high
SAM7bff231634e9	10.25051	1	M	SD	SD/PD	low
SAM7c67b05aa109	8.246407	1	F	SD	SD/PD	high
SAM7d2dfba6cd84	12.71458	1	M	SD	SD/PD	high
SAM7d7c54623618	21.22382	0	M	PR	CR/PR	high
SAM7edacb3deb65	18.00411	0	M	NE	NA	high
SAM7ee2b6e4d6b3	1.708419	1	M	NE	NA	high
SAM7f0d9cc7f001	4.632444	1	M	PD	SD/PD	high
SAM7fb6987514a4	22.83368	0	M	PD	SD/PD	low
SAM7fb7a13c096b	1.675565	1	M	PD	SD/PD	high
SAM80c6183220e6	14.75154	0	M	PD	SD/PD	low
SAM81b71522417a	2.135524	1	F	PD	SD/PD	high
SAM822b226466a1	17.28131	0	M	SD	SD/PD	high
SAM8533e5e261d6	12.81314	1	M	PR	CR/PR	high
SAM85e41e7f33f9	0.62423	1	M	NE	NA	low
SAM85f0a3ac1c45	5.848049	1	M	PD	SD/PD	high
SAM87a8e18eb45b	2.759754	1	F	PD	SD/PD	high
SAM8884fe446d20	23.16222	0	M	PR	CR/PR	low
SAM8a1b0e02ee42	20.56674	0	M	CR	CR/PR	high
SAM8a42c0d59187	2.825462	1	M	NE	NA	high
SAM8b4b8b0f9e73	15.67146	0	M	SD	SD/PD	high
SAM8e43e9caf307	0.492813	1	F	NE	NA	high
SAM8e469834acc1	3.712526	1	M	PD	SD/PD	high
SAM8e8ef2368dfa	4.501027	1	M	PD	SD/PD	high
SAM8f2275c36e8c	20.73101	0	M	SD	SD/PD	high
SAM91c47b054ffb	17.01848	1	M	PR	CR/PR	low
SAM9306c5c92444	15.44148	0	M	PD	SD/PD	low
SAM9410b866974a	6.735113	1	M	SD	SD/PD	low
SAM943df5cf15df	2.135524	1	M	PD	SD/PD	high

SAM9448d858692c	2.036961	1	M	NE	NA	low
SAM94859b440b1d	13.33881	1	M	PD	SD/PD	high
SAM9539a4f19ebc	7.950719	1	M	SD	SD/PD	high
SAM957378bd907f	2.234086	1	M	PD	SD/PD	low
SAM95c70496ffb5	0.361396	1	F	NE	NA	high
SAM961d04c42bd9	5.880903	1	M	PD	SD/PD	high
SAM9681450bbc90	4.960986	1	M	NE	NA	low
SAM9725303dce0c	8.903491	0	M	PD	SD/PD	high
SAM978a587b207e	2.102669	1	M	PD	SD/PD	low
SAM97a00e0929fb	12.71458	0	M	PR	CR/PR	high
SAM99a46b9eec27	3.613963	1	M	PD	SD/PD	high
SAM99b1f6a9534e	0.492813	1	F	NE	NA	high
SAM9a2cf3c06fb3	5.650924	1	M	PD	SD/PD	high
SAM9aa6a095a9d6	8.837782	1	M	PD	SD/PD	high
SAM9b9d48b0b02c	2.89117	1	M	NE	NA	high
SAM9cafb905b36a	7.622177	1	M	PD	SD/PD	low
SAM9d2494119c05	1.445585	1	M	PD	SD/PD	low
SAM9daccafc18db	16.45996	0	F	PD	SD/PD	high
SAM9e11ec6bea80	15.40862	1	M	SD	SD/PD	low
SAM9eebdef2858a	0.821355	1	F	NE	NA	high
SAM9fb814c22bdb	19.12115	1	F	SD	SD/PD	low
SAMa0ca029b7afd	0.361396	1	M	NE	NA	high
SAMa1871f491b02	1.741273	1	F	PD	SD/PD	high
SAMa1e62d323e1d	10.57906	1	F	SD	SD/PD	high
SAMa321770ac31c	7.063655	0	M	PD	SD/PD	low
SAMa424c75831b4	3.548255	1	M	PD	SD/PD	high
SAMa535fcdf18a0	17.28131	0	M	PR	CR/PR	high
SAMa90d73f8d891	1.01848	1	M	PD	SD/PD	high
SAMa913c6139ec8	5.125257	1	M	SD	SD/PD	low
SAMa9ca8536d2b1	21.84805	0	M	SD	SD/PD	high
SAMaabf4afe4213	2.628337	1	M	PD	SD/PD	high
SAMaaf505c36f93	20.56674	0	M	PR	CR/PR	high
SAMab8052a03398	13.30595	1	M	SD	SD/PD	low
SAMabc151b01ea3	0.558522	1	F	PD	SD/PD	low
SAMad83c9c53537	14.98152	0	M	PD	SD/PD	high
SAMae02629a97f7	3.252567	1	F	PD	SD/PD	high
SAMae1690469964	21.15811	1	M	SD	SD/PD	high
SAMae4da274eded	4.99384	1	F	PD	SD/PD	high
SAMaec7380f9ab0	20.86242	0	M	PR	CR/PR	high
SAMaf42c1541269	5.519507	0	M	SD	SD/PD	low
SAMaf7578d55754	0.229979	1	F	NE	NA	low
SAMaff272833538	2.135524	1	M	NE	NA	high

SAMb0a83e5fbde9	23.32649	0	M	SD	SD/PD	high
SAMb0d11db9aa79	10.5462	1	F	PD	SD/PD	low
SAMb15ac6e4c4ef	20.00821	0	M	PR	CR/PR	low
SAMb15ad09d6e24	3.876797	1	F	PD	SD/PD	low
SAMb2e4a082541a	4.36961	1	F	PD	SD/PD	low
SAMb2f1d0e54ece	11.40041	1	M	PR	CR/PR	low
SAMb3c02294aba7	22.40657	0	M	PR	CR/PR	high
SAMb419a8fcbfcd	22.73511	0	M	PR	CR/PR	high
SAMb470eb8f04be	20.07392	0	M	CR	CR/PR	high
SAMb4c7a001537d	3.186858	1	M	PD	SD/PD	high
SAMb8070b7937e7	21.02669	1	M	SD	SD/PD	high
SAMb8101c538753	13.40452	1	M	SD	SD/PD	low
SAMb8f13a0525a6	2.694045	1	M	NE	NA	high
SAMb963dda93cfd	2.759754	1	M	PD	SD/PD	low
SAMba1a34b5a060	12.846	1	M	PD	SD/PD	low
SAMba7176afe070	3.449692	0	F	PD	SD/PD	high
SAMbc8dc3a7b54e	14.75154	1	F	PD	SD/PD	high
SAMbcb07ba81cee	2.529774	1	M	PD	SD/PD	low
SAMbcbc7957c264	4.172485	1	M	PD	SD/PD	high
SAMbd8ee73983b8	0.854209	1	M	NE	NA	high
SAMbda79f955628	20.63244	0	F	SD	SD/PD	low
SAMbe25e2c88f3e	2.924025	1	M	NE	NA	low
SAMbe83eae4026e	2.726899	1	F	PD	SD/PD	high
SAMbf1a3ae828e6	20.73101	0	M	CR	CR/PR	low
SAMbf91f27e7f9b	0.591376	1	M	NE	NA	low
SAMbfdffb97c446	6.702259	1	F	SD	SD/PD	high
SAMc0d625a50eb8	13.2731	1	F	SD	SD/PD	high
SAMc0da5d48686d	9.494867	1	M	PD	SD/PD	high
SAMc0ef41aa6c8b	9.560575	1	M	PD	SD/PD	high
SAMc1251c7bfee2	21.61807	0	M	PR	CR/PR	high
SAMc1b27bc16435	24.1807	0	M	PR	CR/PR	high
SAMc2a1820d4e6b	20.73101	0	M	CR	CR/PR	low
SAMc57eadb2d82b	8.016427	1	M	PD	SD/PD	high
SAMc692536a795a	5.650924	1	M	PD	SD/PD	high
SAMc6eff056c89a	1.806982	1	M	PD	SD/PD	low
SAMc919aebc7fdd	20.73101	0	M	PR	CR/PR	low
SAMc97f35a29d16	0.821355	0	M	NE	NA	high
SAMcabb6d58ff55	20.27105	0	F	SD	SD/PD	low
SAMcb132b0cdd2c	2.529774	1	M	PD	SD/PD	low
SAMcc4675f394a1	3.12115	1	M	PD	SD/PD	low
SAMcc7a42d87e9c	17.21561	0	F	PR	CR/PR	high
SAMce39dd79b441	6.275154	1	M	PD	SD/PD	high

SAMcee0fa8c05b4	1.675565	1	M	NE	NA	low
SAMcf018fee2acd	16.22998	1	M	PD	SD/PD	high
SAMd027124354ce	23.52361	0	F	CR	CR/PR	high
SAMd0e47be700b0	0.887064	1	M	NE	NA	high
SAMd135d5867fe3	3.613963	1	M	PD	SD/PD	low
SAMd1bd63734394	1.938398	1	M	PD	SD/PD	low
SAMd215b503f99a	1.708419	0	M	SD	SD/PD	high
SAMd2492b2a31bb	7.852156	1	F	PD	SD/PD	high
SAMd35318127278	21.58522	0	M	CR	CR/PR	high
SAMd3601288319e	23.12936	0	M	CR	CR/PR	low
SAMd3bd67996035	20.82957	0	M	CR	CR/PR	low
SAMd43f8933066b	20.00821	0	M	PR	CR/PR	low
SAMd4c0837b0997	5.38809	1	M	PD	SD/PD	high
SAMd5ab7fbfab4e	18.10267	0	M	NE	NA	low
SAMd636e3461955	10.48049	1	M	PD	SD/PD	high
SAMd697ba701077	21.15811	1	F	SD	SD/PD	high
SAMd7d57ee3a863	20.27105	0	M	CR	CR/PR	low
SAMd86389d0d768	2.201232	1	M	PD	SD/PD	high
SAMd98bac0a070f	2.069815	1	M	PD	SD/PD	high
SAMda4d892fddc8	14.12731	0	M	PD	SD/PD	low
SAMdab9ca8fb5de	0.197125	0	M	PD	SD/PD	high
SAMdad5c29dc105	2.431211	1	M	PD	SD/PD	high
SAMdb3f50c9129c	15.80287	1	M	PD	SD/PD	high
SAMdcae54fcd7fa	6.2423	1	M	SD	SD/PD	high
SAMdee1011782cd	3.909651	1	M	PD	SD/PD	low
SAMdf3e42c8672a	7.326489	1	M	PD	SD/PD	low
SAMe07c4560772d	2.102669	0	F	PD	SD/PD	high
SAMe0c49ea0df5d	4.13963	1	F	SD	SD/PD	high
SAMe1eb5d988760	21.05955	0	M	CR	CR/PR	low
SAMe3210d3632b4	2.168378	1	F	PD	SD/PD	low
SAMe3d4266775a9	13.99589	0	M	PD	SD/PD	high
SAMe41b1e773582	0.854209	1	M	PD	SD/PD	high
SAMe50d15fde368	18.89117	0	F	PR	CR/PR	high
SAMe56c96c51190	9.7577	1	F	PD	SD/PD	low
SAMe5bc41772bc9	6.110883	1	M	PD	SD/PD	low
SAMe712352fb82a	22.11088	0	M	PR	CR/PR	high
SAMe7bcab05402e	7.917864	1	M	SD	SD/PD	high
SAMe7bf6c015192	3.12115	1	M	PD	SD/PD	low
SAMe7e4f7c076a7	1.971253	1	M	PD	SD/PD	low
SAMe9475f77504b	18.66119	0	M	SD	SD/PD	high
SAMe94c30c30616	15.37577	1	M	SD	SD/PD	high
SAMe97af0feefdf	11.10472	1	F	PD	SD/PD	high

SAMe9ae8beb82fa	5.486653	1	M	PD	SD/PD	high
SAMeaa477a5384b	21.58522	0	M	CR	CR/PR	low
SAMeb29625f76a5	3.12115	1	M	PD	SD/PD	high
SAMeb587a68006b	20.69815	0	M	SD	SD/PD	high
SAMee3844cc0b9f	3.219713	1	M	PD	SD/PD	low
SAMef0e3d2415fd	10.1191	1	M	SD	SD/PD	low
SAMeff2ce356ccb	8.082136	1	M	PD	SD/PD	high
SAMf20b827dca51	4.36961	1	M	PD	SD/PD	low
SAMf275eb859a39	24.47639	0	M	PR	CR/PR	low
SAMf28c01545593	6.406571	1	M	SD	SD/PD	high
SAMf2aae1443f67	10.34908	1	F	PD	SD/PD	high
SAMf2ce197162ce	1.577002	1	M	NE	NA	high
SAMf3a9bce50099	2.102669	0	F	PD	SD/PD	high
SAMf82bbdc267c8	3.12115	1	M	PD	SD/PD	high
SAMfb7aec7cb0e2	22.53799	0	M	PR	CR/PR	low
SAMfd947610629d	9.034908	1	M	PD	SD/PD	high
SAMfddc359e862b	1.938398	1	F	NE	NA	high
SAMfed609955db9	20.56674	0	F	PD	SD/PD	high
SAMff41c4e8c08f	2.464066	1	M	NE	NA	high
SAMffa5c7cad0e5	15.54004	1	M	PR	CR/PR	high

Table.6 Gene Signatures enrolled in this study.

Gene Signature names	Genes set
HP-related gene markers	ATP4A，ADAM17，ITGA5，IL1B，HPSE，TRPML1，CD44，ITGB1，PRTG，SMOX，USF1，MMP10，TRAF1，TRAF2，LC3，GKN1，MCOLN1，CTTN，MRCKβ，CD274，CAPZA1，PDIA3，HIF1A，IL17RB，TRAP1，ETS1，TLR5，NR4A2，CLEC4E，LATS2，ARRDC3，ADM，RACK1，，PUMA，NLRC5，CXCR2，CEACAM1，CEACAM3，CEACAM6
HP-related prognostic genes	DOCK4，HLA-DMA，C3，LAG3，MAFB，CXCR6，VNN2，MSN，IFFO1，ELMO1，CSF1R，C1R，KLHL5，RASSF2，MYO5A，CELF2，PDLIM7，PLEKHO1，EMP3，SHANK3，S1PR1，PTPN14，RHOB，DKK3，FBLN5，ENG，CXCL3，CASP1
mRNAsi	DNMT3B，PFAS，XRCC5，HAUS6，TET1，IGF2BP1，PLAA，TEX10，MSH6，DLGAP5，SKIV2L2，SOHLH2，RRAS2，PAICS，CPSF3，LIN28B，IPO5，BMPR1A，ZNF788，ASCC3，FANCB，HMGA2，TRIM24，ORC1，HDAC2，HESX1，INHBE，MIS18A，DCUN1D5，MRPL3，CENPH，MYCN，HAUS1，GDF3，TBCE，RIOK2，BCKDHB，RAD1，NREP，ADH5，PLRG1，ROR1，RAB3B，DIAPH3，GNL2，FGF2，NMNAT2，KIF20A，CENPI，DDX1，XXYLT1，GPR176，BBS9，C14orf166，BOD1，CDC123，SNRPD3，FAM118B，DPH3，EIF2B3，RPF2，APLP1，DACT1，PDHB，C14orf119，DTD1，SAMM50，CCL26，MED20，UTP6，RARS2，ARMCX2，RARS，MTHFD2，DHX15，HTR7，MTHFD1L，ARMC9，XPOT，IARS，HDX，ACTRT3，ERCC2，TBC1D16，GARS，KIF7，UBE2K，SLC25A3，ICMT，UGGT2，ATP11C，SLC24A1，EIF2AK4，GPX8，ALX1，OSTC，TRPC4，HAS2，FZD2，TRNT1，MMADHC，SNX8，CDH6，HAT1，SEC11A，DIMT1，TM2D2，FST，GBE1
Activated_CD4_T_cell	AIM2，BIRC3，BRIP1，CCL20，CCL4，CCL5，CCNB1，CCR7，DUSP2，ESCO2，ETS1，EXO1，EXOC6，IARS，ITK，KIF11，KNTC1，NUF2，PRC1，PSAT1，RGS1，RTKN2，SAMSN1，SELL，TRAT1
Activated_CD8_T_cell	ADRM1，AHSA1，C1GALT1C1，CCT6B，CD37，CD3D，CD3E，CD3G，CD69，CD8A，CETN3，CSE1L，GEMIN6，GNLY，GPT2，GZMA，GZMH，GZMK，IL2RB，LCK，MPZL1，NKG7，PIK3IP1，PTRH2，TIMM13，ZAP70
Regulatory_T_cell	CCL3L1，CD72，CLEC5A，FOXP3，ITGA4，L1CAM，LIPA，LRP1，LRRC42，MARCO，MMP12，MNDA，MRC1，MS4A6A，PELO，PLEK，PRSS23，PTGIR，ST8SIA4，STAB1
Type_17_T_helper_cell	IL17A，IL17RA，C2CD4A，C2CD4B，CA2，CCDC65，CEACAM3，IL17C，IL17F，IL17RC，IL17RE，IL23A，ILDR1，LONRF3，SH2D6，TNIP2，ABCA1，ABCB1，ADAMTS12，ANK1，ANKRD22，B3GALT2，CAMTA1，CCR9，CD40，GPR44，IFT80
Type_2_T_helper_cell	ASB2，CSRP2，DAPK1，DLC1，DNAJC12，DUSP6，GNAI1，LAMP3，NRP2，OSBPL1A，PDE4B，PHLDA1，PLA2G4A，RAB27B，RBMS3，RNF125，TMPRSS3，GATA3，BIRC5，CDC25C，CDC7，CENPF，CXCR6，DHFR，EVI5，GSTA4，HELLS，IL26，LAIR2
Activated_dendritic_cell	ABCD1，C1QC，CAPG，CCL3L3，CD207，CD302，ATP5B，ATP5L，ATP6V1A，BCL2L1，C1QB，SNURF，SPCS3，CCNA1，CEACAM8，NOS2，SRA1，TNFRSF6B，TREM1，TREML1，RHOA，SLC25A37，TNFSF14，TREML4，VNN2，XPO6，CLEC4C，TNFAIP2，UBD，ACTR3，RAB1A，SLA，HLA-DQA2，SIGLEC5，SLAMF9
Immature_dendritic_cell	ACADM，AHCYL1，ALDH1A2，ALDH3A2，ALDH9A1，ALOX15，AMT，ARL1，ATIC，ATP5A1，CAPZA1，LILRA5，RDX，RRAGD，TACSTD2，INPP5F，RAB38，PLAU，CSF3R，SLC18A2，AMPD2，CLTB，C1orf162

BMDC	CCR2，CD14，CD2，CD86，CXCR4，FCGR2A，FCGR2B，FCGR3A，FERMT3，GPSM3，IL18BP，IL4R，ITGAL，ITGAM，PARVG，PSAP，PTGER2，PTGES2，S100A8，S100A9，CD40，CD45，CD14，CD206，CD1c，CD11c，CD80，CD86
Plasmacytoid_dendritic_cell	CBX6，DAB2，DDX17，HIGD1A，IDH3A，IL3RA，MAGED1，NUCB2，OFD1，OGT，PDIA4，SERTAD2，SIRPA，TMED2，ENG，FCAR，IGF1，ITGA2B，GABARAP，GPX1，KRT23，PROK2，RALB，RETNLB，RNF141，SEC14L1，SEPX1，EMP3，CD300LF，ABTB1，KLHL21，PHRF1
Macrophage	AIF1，CCL1，CCL14，CCL23，CCL26，CD300LB，CNR1，CNR2，EIF1，EIF4A1，FPR1，FPR2，FRAT2，GPR27，GPR77，RNASE2，MS4A2，BASP1，IGSF6，HK3，VNN1，FES，NPL，FZD2，FAM198B，HNMT，SLC15A3，CD4，TXNDC3，FRMD4A，CRYBB1，HRH1，WNT5B
Check-point	IDO1，LAG3，CTLA4，TNFRSF9，ICOS，CD80，PDCD1LG2，TIGIT，CD70，TNFSF9，ICOSLG，KIR3DL1，CD86，PDCD1，LAIR1，TNFRSF8，TNFSF15，TNFRSF14，IDO2，CD276，CD40，TNFRSF4，TNFSF14，HHLA2，CD244，CD274，HAVCR2，CD27，BTLA，LGALS9，TMIGD2，CD28，CD48，TNFRSF25，CD40LG，ADORA2A，VTCN1，CD160，CD44，TNFSF18，TNFRSF18，BTNL2，C10orf54，CD200R1，TNFSF4，CD200，NRP1
REACTOME_TNF_SIGNALING	ADAM17，BAG4，BIRC2，BIRC3，CASP8，CFLAR，CHUK，CLIP3，CYLD，FADD，IKBKB，IKBKG，MADD，MAP3K7，NSMAF，OTUD7B，OTULIN，RACK1，RBCK1，RIPK1，RNF31，RPS27A，SHARPIN，SMPD2，SMPD3，SPPL2A，SPPL2B，TAB1，TAB2，TAB3，TAX1BP1，TNF，TNFAIP3，TNFRSF1A，TRADD，TRAF1，TRAF2，UBA52，UBB，UBC，USP2，USP21，USP4，XIAP
REACTOME_REGULATION_OF_IFNA_SIGNALING	IFNA1，IFNA10，IFNA13，IFNA14，IFNA16，IFNA17，IFNA2，IFNA21，IFNA4，IFNA5，IFNA6，IFNA7，IFNA8，IFNAR1，IFNAR2，IFNB1，JAK1，PTPN1，PTPN11，PTPN6，SOCS1，SOCS3，STAT1，STAT2，TYK2，USP18
BIOCARTA_IL17_PATHWAY	CD2，CD247，CD34，CD3D，CD3E，CD3G，CD4，CD58，CD8A，CSF3，CXCL8，IL17A，IL3，IL6，KITLG
CD8_T_effector	CD8A，CXCL10，CXCL9，GZMA，GZMB，IFNG，PRF1，TBX21
Antigen_processing_machinery	B2M，HLA-A，HLA-B，HLA-C，TAP1，TAP2
EMT1	CLDN3，CLDN7，CLDN4，CDH1，VIM，TWIST1，ZEB1，ZEB2
EMT2	AXL，FAP，LOXL2，ROR2，TAGLN，TWIST2，WNT5A
EMT3	FOXF1，GATA6，SOX9，TWIST1，ZEB1，ZEB2
Pan_F_TBRS	ACTA2，ACTG2，ADAM12，ADAM19，CNN1，COL4A1，CTGF，CTPS1，FAM101B，FSTL3，HSPB1，IGFBP3，PXDC1，SEMA7A，SH3PXD2A，TAGLN，TGFBI，TNS1，TPM1
Angiogenesis	CDH5，SOX17，SOX18，TEK
Fanconi_anemia	APITD1，ATR，ATRIP，BLM，BRCA1，BRCA2，BRIP1，C17orf70，C19orf40，EME1，EME2，ERCC1，ERCC4，FAN1，FANCA，FANCB，FANCC，FANCD2，FANCE，FANCF，FANCG，FANCI，FANCL，FANCM，HES1，MLH1，MUS81，PALB2，PMS2，POLH，POLI，POLK，POLN，RAD51，RAD51C，REV1，REV3L，RMI1，RMI2，RPA1，RPA2，RPA3，RPA4，SLX4，STRA13，TELO2，TOP3A，TOP3B，UBE2T，USP1，WDR48
Nucleotide_excision_repair	CCNH，CDK7，CETN2，CUL4A，CUL4B，DDB1，DDB2，ERCC1，ERCC2，ERCC3，ERCC4，ERCC5，ERCC6，ERCC8，GTF2H1，GTF2H2，GTF2H3，GTF2H4，GTF2H5，LIG1，MNAT1，PCNA，POLD1，POLD2，POLD3，POLD4，POLE，POLE2，POLE3，POLE4，RAD23A，RAD23B，RBX1，RFC1，RFC2，RFC3，RFC4，RFC5，RPA1，RPA2，RPA3，RPA4，XPA，XPC
DNA_damage_repair	ALKBH2，ALKBH3，APEX1，APEX2，APLF，ATM，ATR，ATRIP，BLM，BRCA1，BRCA2，BRIP1，CCNH，CDK7，CETN2，CHAF1A，CHEK1，CHEK2，CLK2，DCLRE1C，DDB1，DDB2，

	DUT，ENDOV，ERCC1，ERCC2，ERCC3，ERCC4，ERCC5，ERCC6，ERCC8，FAN1，FANCA，FANCB，FANCC，FANCD2，FANCE，FANCF，FANCG，FANCL，FANCM，GTF2H1，GTF2H2，GTF2H3，GTF2H4，GTF2H5，H2AFX，HLTF，HUS1，LIG1，LIG3，LIG4，MBD4，MDC1，MGMT，MLH1，MLH3，MMS19，MNAT1，MPG，MSH2，MSH3，MSH4，MSH5，MSH6，MUTYH，NEIL1，NEIL2，NEIL3，NHEJ1，NTHL1，NUDT1，OGG1，PALB2，PARP1，PARP2，PARP3，PCNA，PER1，PMS1，PMS2，PNKP，POLB，POLD1，POLE，POLG，POLH，POLL，POLM，POLQ，PRKDC，RAD1，RAD17，RAD18，RAD23A，RAD23B，RAD51C，RAD9A，RECQL4，RECQL5，RIF1，RNF168，RNF4，RNF8，RPA1，RPA2，RPA3，RPA4，RRM2B，SETMAR，SHPRH，SMUG1，TDP1，TDP2，TOPBP1，TP53，TREX1，UBE2A，UBE2B，UBE2N，UBE2V2，UNG，UVSSA，WRN，XAB2，XPA，XPC，XRCC1，XRCC4，XRCC5，XRCC6
Homologous_recombination	BLM，BRCA2，EME1，MRE11A，MUS81，NBN，POLD1，POLD2，POLD3，POLD4，RAD50，RAD51，RAD51B，RAD51C，RAD51D，RAD52，RAD54B，RAD54L，RPA1，RPA2，RPA3，RPA4，SHFM1，SSBP1，TOP3A，TOP3B，XRCC2，XRCC3
Mismatch_repair	EXO1，LIG1，MLH1，MLH3，MSH2，MSH3，MSH6，PCNA，PMS2，POLD1，POLD2，POLD3，POLD4，RFC1，RFC2，RFC3，RFC4
WNT_target	EFNB3，MYC，TCF12，VEGFA
Cell_cycle_regulators	ATM，CCND1，CCNE1，CDKN1A，CDKN2A，E2F3，FBXW7，MDM2，RB1，TP53
