# Supplementary material for: Integrated analysis of Helicobacter pylori-related prognostic gene modification patterns in the tumour microenvironment of gastric cancer
Source: Front Surg. 2022 Sep 30;9:964203. doi: 10.3389/fsurg.2022.964203 (PMC9561901; doi:10.3389/fsurg.2022.964203)
Supplement: Supplementary file 2 [file DataSheet1.docx]

# Integrated analysis of Helicobacter pylori-related prognostic gene modification patterns in the tumour microenvironment of gastric cancer

Kaitian Zheng^1#^, Ye Wang^1#^, Jiancheng Wang^1^, Congjun Wang^1^, Junqiang Chen^2*^

1. Guangxi Medical University, No.6, Shuangyong Road, Qingxiu District, Nanning City, Guangxi Zhuang Autonomous Region, China.
2. Department of Gastrointestinal Gland Surgery, The First Affiliated Hospital of Guangxi Medical University, Nanning City, Guangxi Zhuang Autonomous Region, 530021, China.

# Ye Wang and Kaitian Zheng contributed equally to this work.

*** Correspondence:**Junqiang Chen

chenjunqiang@gxmu.edu.cn

**Fig. S1:** Data preprocessing steps.

**Fig. S2:** The expression pattern and correlation analysis of HP-related prognostic genes.

**Fig. S3:** External data sets verify the application value of prognostic risk models.

**Fig. S4:** Basic mode and mixture coefficients of Non-negative matrix factorization (NMF).

**Fig. S5:** The construction of HPscore and the association among HPscore.

**Fig. S1:** Data preprocessing steps. (A) ROC curves to compare the diagnostic accuracy in GSE61433, GSE60642, and 27411 data sets. (B) Overall survival of the gastric cancer data sets. (C) Removal of batch effect. (D) Principal component analysis of combined data sets before and after batch effect correction.


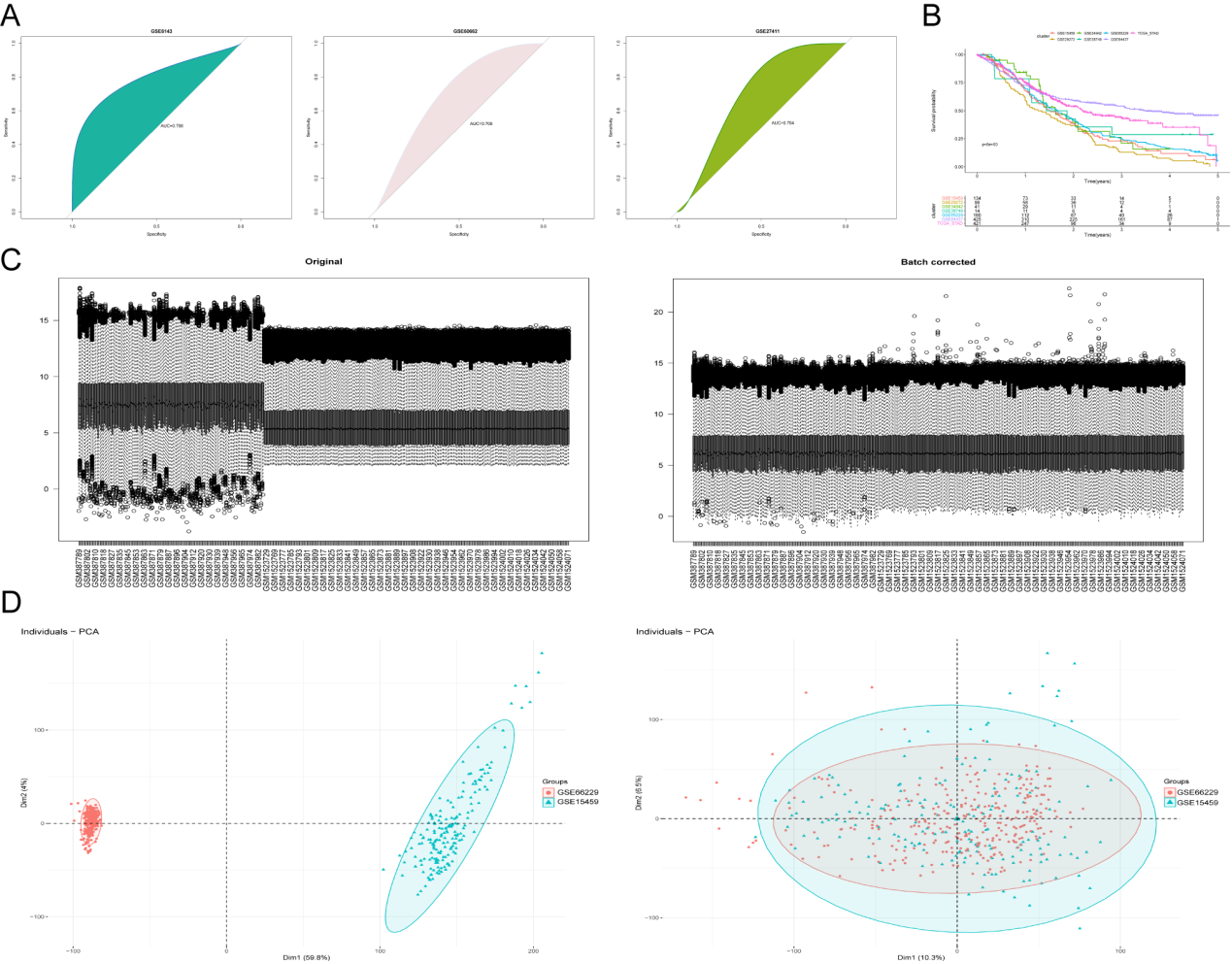


**Fig. S2:** The expression pattern and correlation analysis of HP-related prognostic genes. (A) Upset diagram displaying the overlap of differential gene expression profiles. (B) Chart of the expression correlation between 28 HP-related prognostic genes.


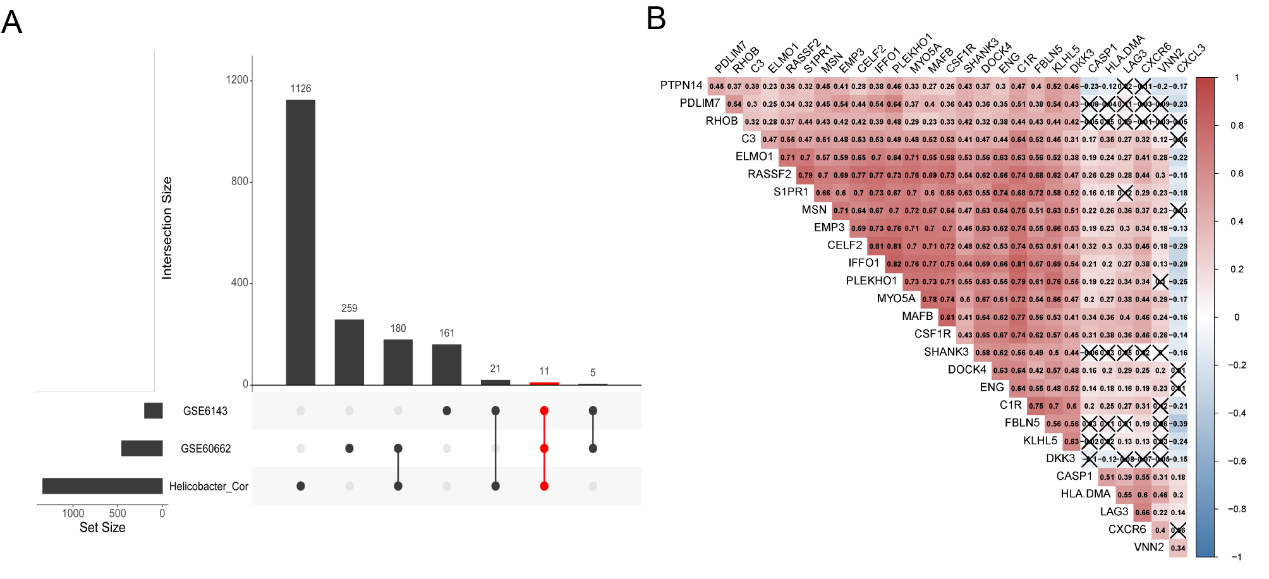


**Fig. S3:** External data sets verify the application value of prognostic risk models. (A) Heatmap, the risk score, and patients’ survival status principal component analysis (PCA) shows the clustering effect of the risk model in the external datasets (GSE29272, GSE84437). (B) Box plot results show that HP-related gene expression is significantly different in the high-risk and low-risk groups.


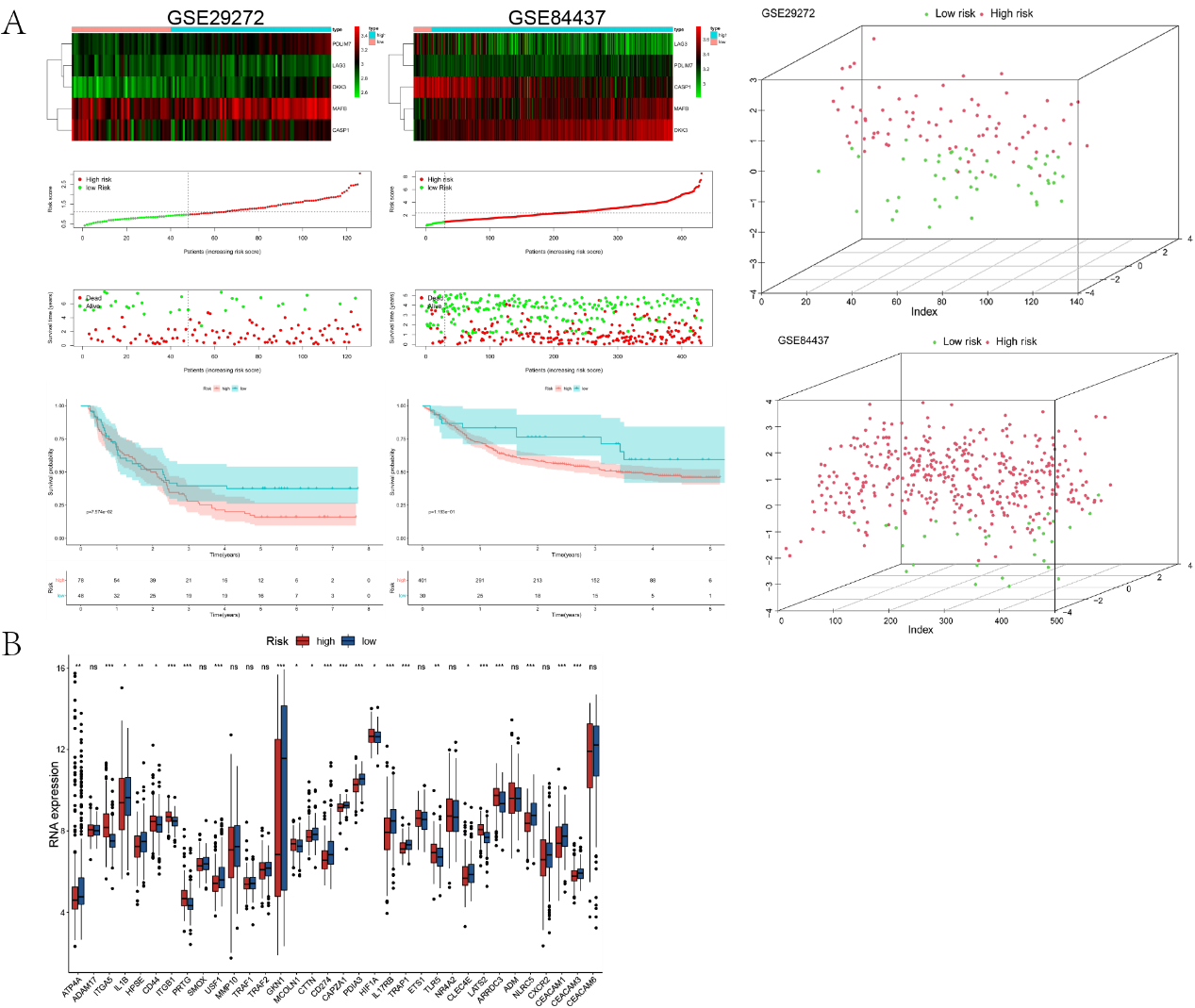


**Fig. S4:** (A) and (B) Basic mode and mixture coefficients of Non-negative matrix factorization (NMF). (C) Expression of different genes in gastric cancer and adjacent in TCGA-STAD.


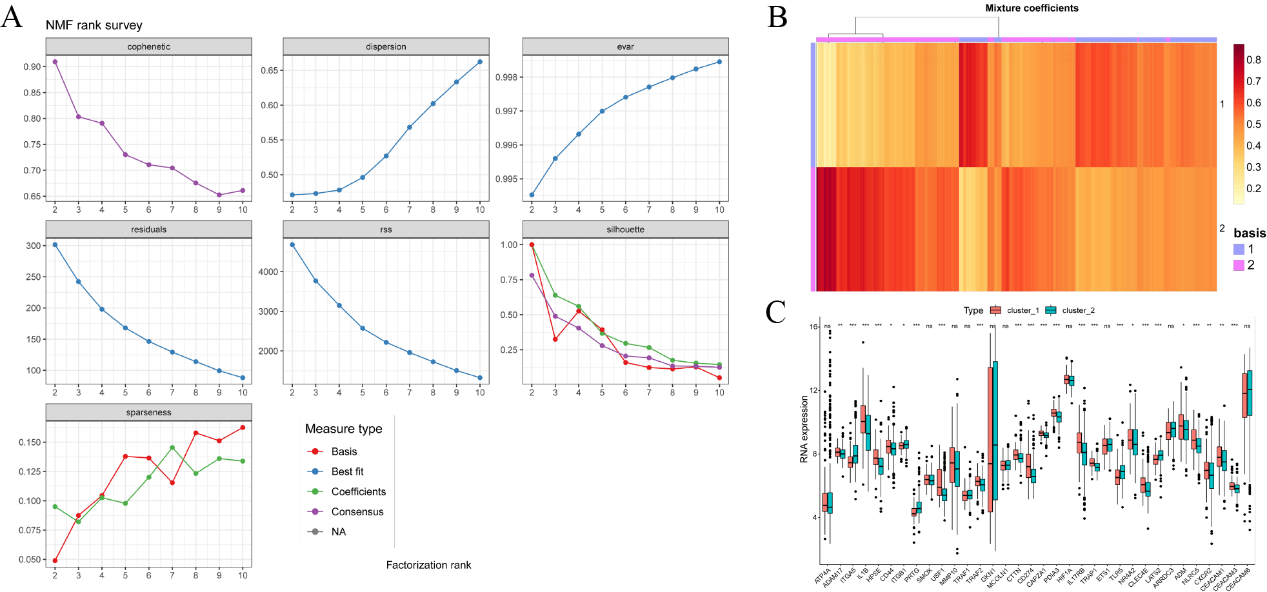


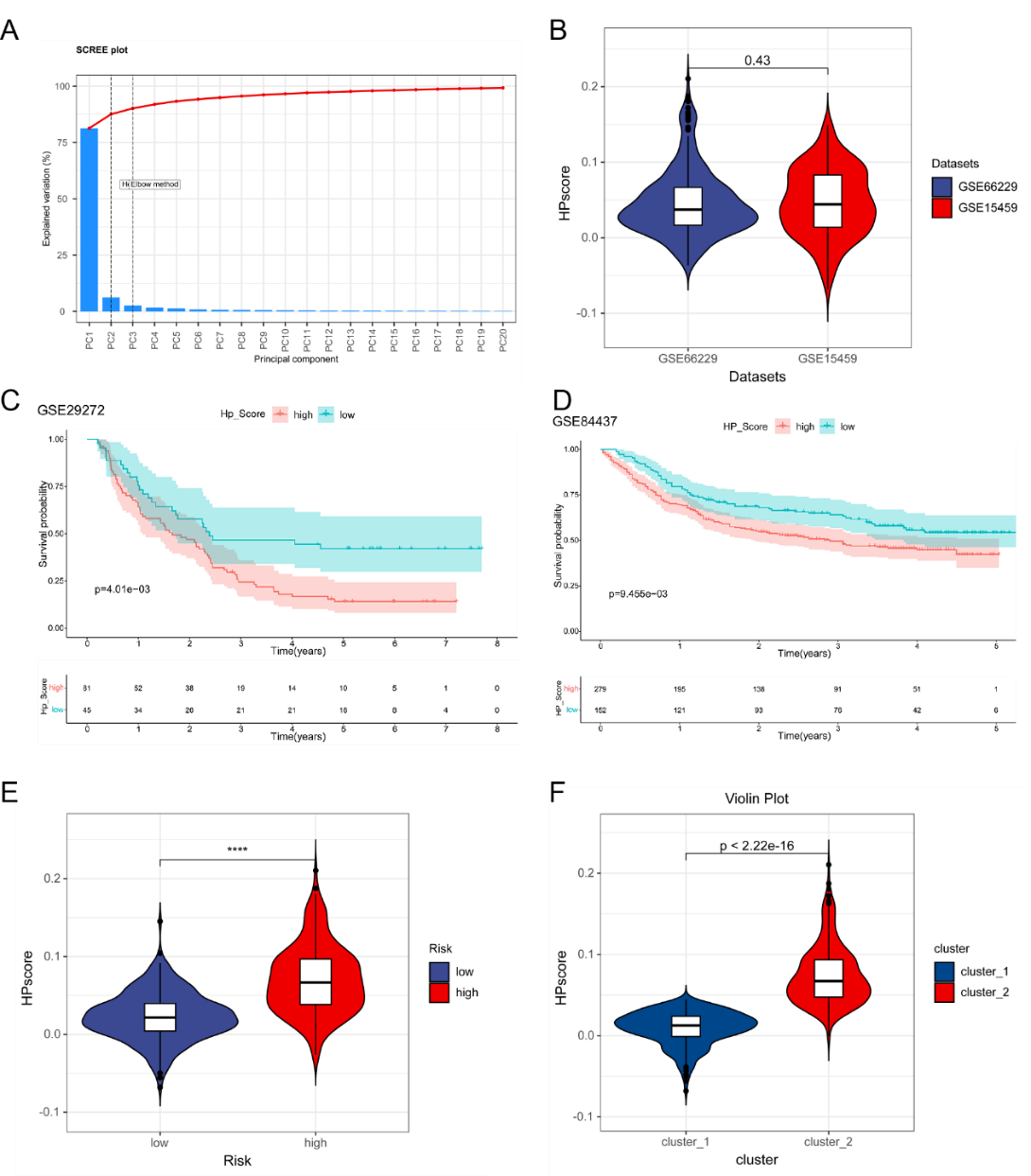
**Fig. S5:** The construction of HPscore and the association among HPscore, Age, and Gender. (A) Scree plot based on the principal component analysis. (B) Difference of HPscores with internal datasets (GSE66229, GSE15459). (C) and (D) Overall survival analysis verified HPscore system in external datasets (GSE29272 and GSE84437). (E) and (F) The HPscore difference analysis in the risk groups and cluster groups.
